# Is oral consumption of dates (*Phoenix dactylifera L.* fruit) in the peripartum period effective and safe integrative care to facilitate childbirth and improve perinatal outcomes: a comprehensive revised systematic review and dose-response meta-analysis

**DOI:** 10.1186/s12884-023-06196-y

**Published:** 2024-01-02

**Authors:** Zahra Salajegheh, Morteza Nasiri, Mohammad Imanipour, Mina Zamanifard, Omid Sadeghi, Mohammad Ghasemi Dehcheshmeh, Masoomeh Asadi

**Affiliations:** 1 Department of Medical-Surgical Nursing, School of Nursing and Midwifery, Kerman University of Medical Sciences, Kerman, Iran; 2 Department of Anesthesiology, School of Allied Medical Sciences, Tehran University of Medical Sciences, Tehran, Iran; 3 Department of Medical-Surgical Nursing, School of Nursing, Aja University of Medical Sciences, Tehran, Iran; 4 Department of Pediatric Nursing, School of Nursing and Midwifery, Jahrom University of Medical Sciences, Jahrom, Iran; 5 Nutrition and Food Security Research Center, Department of Community Nutrition, School of Nutrition and Food Science, Isfahan University of Medical Sciences, Isfahan, Iran; 6 Department of Immunology, School of Medicine, Ahvaz Jundishapur University of Medical Sciences, Ahvaz, Iran; 7 Religion, Health and Technology Studies Center, Abadan University of Medical Sciences, P.O. Box 6313833177, Abadan, Iran

**Keywords:** Date fruit, Maternal-child nursing, Perinatal care, Peripartum, *Phoenix dactiylifera*, Systematic review

## Abstract

**Background:**

Recent reviews have reported inconclusive results regarding the usefulness of consuming dates (*Phoenix dactylifera L.* fruit) in the peripartum period. Hence, this updated systematic review with meta-analysis sought to investigate the efficacy and safety of this integrated intervention in facilitating childbirth and improving perinatal outcomes.

**Methods:**

Eight data sources were searched comprehensively from their inception until April 30, 2023. Parallel-group randomized and non-randomized controlled trials published in any language were included if conducted during peripartum (i.e., third trimester of pregnancy, late pregnancy, labor, or postpartum) to assess standard care plus oral consumption of dates versus standard care alone or combined with other alternative interventions. The Cochrane Collaboration’s Risk of Bias (RoB) assessment tools and the Grading of Recommendations Assessment, Development, and Evaluation (GRADE) were employed to evaluate the potential RoB and the overall quality of the evidence, respectively. Sufficient data were pooled by a random-effect approach utilizing Stata software.

**Results:**

Of 2,460 records in the initial search, 48 studies reported in 55 publications were included. Data were insufficient for meta-analysis regarding fetal, neonatal, or infant outcomes; nonetheless, most outcomes were not substantially different between dates consumer and standard care groups. However, meta-analyses revealed that dates consumption in late pregnancy significantly shortened the length of gestation and labor, except for the second labor stage; declined the need for labor induction; accelerated spontaneity of delivery; raised cervical dilatation (CD) upon admission, Bishop score, and frequency of spontaneous vaginal delivery. The dates intake in labor also significantly reduced labor duration, except for the third labor stage, and increased CD two hours post-intervention. Moreover, the intervention during postpartum significantly boosted the breast milk quantity and reduced post-delivery hemorrhage. Likewise, dates supplementation in the third trimester of pregnancy significantly increased maternal hemoglobin levels. The overall evidence quality was also unacceptable, and RoB was high in most studies. Furthermore, the intervention’s safety was recorded only in four trials.

**Conclusion:**

More well-designed investigations are required to robustly support consuming dates during peripartum as effective and safe integrated care.

**Trial registration:**

PROSPERO Registration No: CRD42023399626

**Supplementary Information:**

The online version contains supplementary material available at 10.1186/s12884-023-06196-y.

## Introduction

Despite the considerable scientific efforts invested in exploring effective and safe methods for childbirth progress, induction of labor (IOL) has been widely utilized when this progress is inappropriate [1]. The prevalence of IOL varies from area to area, yet this intervention is conducted in about 20% and 25% of all births in developing and developed countries, respectively [2]. Although IOL is a crucial life-saving practice that potentially prevents perinatal complications, it is not always successful [3]. Based on a recent cross-sectional study, three out of four parturients who had IOL achieved a vaginal delivery [4]. The pooled prevalence of unsuccessful IOL was also reported to be 23.58% in a systematic review; however, the magnitude of this condition depends on induction guidelines and maternal factors [5]. In addition to the risk of failed IOL, this practice could be associated with some undesired outcomes, such as longer labor stages and excessive uterine contractions, which may raise the risk of uterine rupture, postpartum hemorrhage (PPH), and birth asphyxia, as well as the need for cesarean section (C/S) and instrumental births [6]. Furthermore, IOL might lead to a substantial economic burden and inconvenience for parturients due to their restricted mobility and continuous fetal heart rate monitoring [7]. Likewise, misusing oxytocin and prostaglandins usually administered for IOL can result in adverse perinatal outcomes [8]. Thus, using safe integrative caring interventions to facilitate childbirth and improve perinatal outcomes is valuable in maternal-neonatal health nursing.

Herbal products have been one of the most used complementary methods for facilitating labor progress in many traditions because they are safer, lower-cost, and easier to access than pharmaceutical drugs [9, 10]. Many self-prescribed medicinal plants and herbs have been used globally during peripartum for safe delivery and fetus well-being, such as evening primrose, raspberry, castor bean, fennel, saffron, pennyroyal, sisymbrium, peganum, dill, chasteberry, and chamomile [11–16]. However, *Phoenix dactylifera L* (*P. dactylifera*), generally known as date palm, has attracted researchers’ interest more seriously over the past years, especially in the Middle East and Islamic Traditional Medicine [17–19].

The consumption of date palm fruit (DPF), commonly named dates, is a typical behavior among women from the Middle East during the final month of gestation [20]. In different traditional medicines, DPF is also highly recommended to be consumed by parturients and breastfeeding mothers [21]. Likewise, based on Islamic narrations and verses of the holy Quran (the leading Islamic religious book), eating DPF is favorably proposed in late pregnancy and labor for safe childbirth and promoting maternal and neonatal health [22, 23]. In the holy Quran, *P. dactylifera* was highly glorified, and its heavenly fruit was presented as a beneficial diet to the Virgin Mary when she gave birth to Prophet Issa (peace be upon him). According to hadiths, God would not have recommended DPF to Mary if it was an inappropriate food source [24]. The DPF has substantial fructose, glucose, tannins, serotonin, linoleic and linolenic fatty acids, calcium, iron, potassium, magnesium, estrogen, progesterone, potuchsin hormone, and oxytocin-like agents. These ingredients all cause satisfactory childbirth and perinatal outcomes, including but not limited to strengthening maternal energy, stimulating the uterine muscle contractions, accelerating the spontaneity of labor and uterine involution process, declining labor pain, facilitating placental abruption, increasing parturients’ hemoglobin (Hb) levels and controlling their blood pressure, reducing PPH, and boosting the mother’s breast milk production [21, 25–27].

Despite the scientific rationale behind the beneficial effects of eating DPF in peripartum, some trials do not robustly support this practice. It was reported that maternal cervical dilatation (CD); delivery mode; and/or the score of neonatal appearance, pulse, grimace, activity, and respiration (APGAR) did not significantly change between parturients who ingested DPF in late pregnancy or labor and those who only received routine obstetric and nursing care [28–30]. Besides, no substantial differences were reported in the length of labor stages between the dates consumption and control groups [28, 29, 31–37]. Further, the efficacy of using DPF on labor bleeding or PPH was similar to standard care [28, 29, 38, 39]. Also, there was no significant increase in maternal’ Hb levels after daily consumption of DPF [28, 40–42].

In addition to trials, recent systematic reviews or meta-analyses have reported contradicting findings on the usefulness of consuming DPF in late pregnancy or labor [43–47]. Previous studies mainly limited the publication’s searches regarding databases, languages, or locations; thus, they have missed several related trials. Additionally, the last corresponding systematic review with meta-analysis screened publications up to August 2019 [43]; however, some relevant studies have been published since then. Therefore, by performing a comprehensive search in different appropriate data sources, this updated systematic review aimed to summarize and statistically pool the results of all available non-randomized and randomized controlled trials (RCTs) published in any language regarding the effects of oral intake of DPF in the peripartum period on childbirth progress and perinatal outcomes.

## Methods

This review observed the last guideline of Preferred Reporting Items for Systematic Review and Meta-Analyses (PRISMA) (Supplementary Table 1) [48]. The formal ethical assessment was obtained from Abadan University of Medical Sciences, Abadan, Iran (No. IR.ABADANUMS.REC.1401.164). Additionally, the protocol was documented in the International Prospective Register of Systematic Reviews (PROSPERO, No. CRD42023399626).

### Eligibility criteria

The trials published in peer-reviewed journals in any language were eligible if they had the criteria presented in Table 1. Limitations were not considered in the inclusion criteria concerning women’s parity, gravidity, and gestational age, as well as intervention frequency, duration, and time. Moreover, if eligible articles were multiple reports of one trial with analysis of different intended study outcomes, all were retained; instead, the results were incorporated in the meta-analysis once to ignore overlapping participants.

**Table 1:** Inclusion criteria for considering trials on the effects of oral consumption of dates in the peripartum period on childbirth and perinatal outcomes

**Items**		**Criteria**
Participants		Women aged 18-45 years who were in the third trimester of pregnancy (28-40 weeks of gestation), late pregnancy (32-42 weeks of gestation), intrapartum (the onset of labor to delivery of the neonate and the placenta), or postpartum (immediately after the placenta delivery to five months post-delivery) without a history of high-risk pregnancy, serious perinatal problems, or severe post-delivery complications
Intervention		Administration of standard interventions plus oral consumption of dates fruit in any forms (i.e., pure, syrup, juice, extract, essence, or decoction) during peripartum (i.e., third trimester of pregnancy, late pregnancy, labor, or postpartum)
Comparison		Administration of standard interventions alone or combined with alternative interventions during peripartum (i.e., third trimester of pregnancy, late pregnancy, labor, or postpartum)
Outcomes	Primary	1- Maternal indices: a) labor progress represented by the duration of different labor stages, cervical dilatation, Bishop score, type of labor onset (i.e., spontaneous or with augmentation and induction), delivery mode (i.e., spontaneous/instrumental vaginal delivery, C/S delivery), and uterine contractions; b) gestation length; c) labor pain severity; d) breast milk production; e) labor or postpartum bleeding rate; and f) maternal hemoglobin levels
	Secondary	2- Fetal, neonatal, or infant indices: a) fetal heart rate; b) fetal presentation status; c) presence of meconium liquor staining; d) neonatal APGAR score; e) neonatal birth weight; f) neonatal admission rate to the intensive care unit; and g) infant weight gain 3- Adverse effects of intervention with dates consumption
Study design		Parallel-group randomized and non-randomized controlled trials

*Abbreviations*: *APGAR score* Appearance, pulse, grimace, activity, and respiration, *C/S* Cesarean section.

The studies were excluded if they: 1) followed a one-group pre-test/post-test approach, 2) had insufficient data on the intervention method, 3) were a non-human study, thesis, dissertation, book chapter, review, or conference proceeding, 4) conducted intervention in the first or second trimester of gestation, 5) recruited participants aged less than 18 or more than 45 years, 6) included women with a history of high-risk pregnancy, serious antenatal problems, or severe post-delivery complications, 7) administered dates’ products in combination with other fruit-based or herbal-based remedies, or 8) used either DPF or other carbohydrate sources based on women demands, and the number of dates consumers was unclear.

### Search characteristics

A comprehensive search was accomplished in three international databases (i.e., Cochrane Library, Scopus, and Web of Science Core Collection) and two search engines (i.e., PubMed and Google Scholar). Besides, a search was performed in the Scientific Content Database of the Islamic World Science Citation Center (ISC) to find further non-English publications (e.g., Persian, Arabic, or Indonesian). Also, the Iranian Registry of Clinical Trials (IRCT) and the International Clinical Trials Registry Platform (ICTRP) were screened for register entries of trials. Moreover, the references of pertinent publications were hand-searched for extra related articles.

The search strategy consisted of different vocabularies and synonyms of *P. dactylifera* merged with related medical subject headings (MeSH) and keywords. The search syntax for each data source is available in Supplementary Table 2. First, data sources were searched during February 2023. Then, a complementary search was conducted in April 2023 to obtain new articles. The publication date was not limited to ensure all relevant trials were included. Two independent investigators (ZS, MN) performed the search, and any uncertainty or dispute between them was fixed through consensus adjudication.

### Studies selection and data management

First, all retrieved records were transferred to Endnote software. Then, duplications were dismissed, and the remaining records were screened for eligibility based on titles, abstracts, and keywords. In the next step, the full text of potential eligible publications was inspected to prove their eligibility. Finally, a data extraction form was utilized to document the main details of each included trial, including authors’ names, publication date and language, study design and country of origin, participants’ characteristics, sample size, intervention protocol, control conditions, and findings (i.e., means and standard deviations [SDs] or number and percentage of intended outcomes in addition to any reported adverse effects). Besides, we extracted dates’ administration frequency, dosage, and duration for dose-response analysis. If a study did not report the consumed numbers or weights of DPF, the data were estimated based on the administration dosage presented in a similar included research; otherwise, 10-11 pieces of DPF were considered as ~ 100 grams [49]. Also, we calculated the difference between the first and last consumption times for an indefinite administration duration.

In addition to extracting the characteristics mentioned above, the quality of each trial was addressed by utilizing the Cochrane Collaboration’s Risk of Bias (RoB) assessment tools. To this end, the RoB in Non-randomized Studies-of Interventions (ROBINS-I) and the RoB2 tools were used for non-RCTs and RCTs, respectively [50, 51]. Further, criteria suggested by the Grading of Recommendations Assessment, Development, and Evaluation Working Group (GRADE) were employed to judge the overall evidence quality [52].

Screening of the retrieved records and extracting the data from the included studies were performed independently by two researchers (MI, MZ). If an article contained insufficient information regarding the implemented interventions or findings, the principal author was contacted via E-mail to get the missing data. Any disagreement among the researchers was settled by in-depth discussion within the research team.

### Data analysis

If at least three studies documented the same outcomes, their data were pooled through the meta-analysis. A random-effects model was utilized to compute the risk ratio (RR) or weighted mean difference (WMD) with a corresponding 95% confidence interval (CI). The I-squared statistic (*I*^*2*^) and Cochran’s *Q* test were applied to show between-study heterogeneity and the degree of inconsistency [53]. Since the pooled effect sizes (ESs) were less than ten, a contour-enhanced funnel plot was not drawn for publication bias; instead, Egger’s and Begg’s tests were executed [54]. If substantial publication bias was found, the trim-and-fill technique was used. Also, to estimate the standard administration dosage and duration of DPF to bring maximum results, the non-linear dose-response analysis was applied by fractional polynomial modeling. Furthermore, other supplementary investigations (i.e., sensitivity, subgroup, or meta-regression) were employed where applicable. The statistical analyses were run using Stata, version 11.2 (Stata Corp., College Station, TX, USA). A *P* < 0.05 was supposed to be significant.

## Results

### Studies screening and selection

The study identification and selection details are visualized in Supplementary Fig. 1. After screening 2,460 identified records, 33 were excluded based on full-text evaluation (Supplementary Table 3). Finally, 55 publications were considered eligible for this review. Of these, three articles represented overlapping populations; each reported a different intended outcome [55–57]. Two sets of publications also had such a condition [58–61]. Additionally, three articles had an identical registry code and were separate reports of a single trial [62–64]. Similarly, two other articles were redundant publications [65, 66]. Accordingly, a total of 48 studies, documented in 55 articles, were included in the current review.

### Description of the included publications

The main characteristics of the included articles are summarized in Table 2. They were published in Indonesian (*n*= 25), English (*n*= 21), or Persian (n= 9) from 2007 to March 2023. The studies were performed in Indonesia (*n*= 30), Iran (*n*= 9), Egypt (*n*= 2), Saudi Arabia (*n*= 2), Pakistan (*n*= 2), Jordan (*n*= 1), Malaysia (*n*= 1), and Thailand (*n*= 1). Fifteen trials used random allocation, and the remaining 33 studies followed a non-randomized design. Sample sizes of studies ranged from 10 to 105 per group.

**Table 2:** Summary of the 48 included trials on the effects of oral consumption of dates in the peripartum period on childbirth and perinatal outcomes

**Authors, publication date (country)**	**Study design**	**Participants**			**Interventions**				**Outcomes**^**††**^** (measurement times)**	**Findings**^†^^**†**^^†^	**Overall RoB**
		**Parity (P), Gravidity (G)**	**Gestational age at recruitment or delivery (week)**	**Sample size; maternal age (years, M±SD)**	**Intervention time (frequency, duration)**	**Comparison arm**	**Experimental arm (dates consumption)**				
							**Consumed form (dates’ ripening stage**^**†**^**, variety)**	**Consumed dosage → Total**			
Choirunissa et al., 2021 (Indonesia) [67]	2-arm non-RCT	n.r	n.r	E: 16; n.r C: 16; n.r	The 3^rd^ trimester of pregnancy (daily, 14 d)	Iron supplementation (i.e., ferrous sulfate tablet)	Pure (Tamer, n.r)	7 dates/d (~80 gr/d) **→** 14 d, 98 dates, ~1,120 gr	Hb level (T0: baseline, T1: end of intervention time)	Sig ↑ at T1	Serious ^a^
Dahlan & Ardhi, 2021 (Indonesia) [68]	2-arm non-RCT	n.r	n.r	E: 15; n.r C: 15; n.r	The 3^rd^ trimester of pregnancy (daily, n.r)	Iron supplementation (i.e., ferrous sulfate tablet)	Pure (Tamer, Egyp)	n.r	Hb level (T0: baseline, T1: end of intervention time)	Sig ↑ at T1	Serious ^a^
Fauziah & Maulany, 2021 (Indonesia) [69]	2-arm non-RCT	n.r	n.r	E: 11; n.r C: 11; n.r	The 3^rd^ trimester of pregnancy (daily, 10 d)	Iron supplementation (i.e., 60 mg of ferrous sulfate and 0.400 mg of folic acid)	Pure (Tamer Tunisia)	~7 dates/d (75 gr/d) **→** 10 d, ~70 dates, 750 gr	Hb level (T0: baseline, T1: end of intervention time)	Sig ↑ at T1	Serious ^a^
Manan et al., 2021 (Indonesia) [70]	2-arm non-RCT	n.r	n.r	E: 11; n.r C: 11; n.r	The 3^rd^ trimester of pregnancy (daily, 7 d)	Iron supplementation (i.e., ferrous sulfate tablet)	Pure (Tamer, Ajwa)	7 dates/d (~80 gr/d) in the morning **→** 7 d, 49 dates, ~560 gr	Hb level (T0: baseline, T1: end of intervention time)	Sig ↑ at T1	Serious ^a^
Murtiyarini et al., 2021 (Indonesia) [71]	2-arm non-RCT	P: 1-5	28-39	E: 30; n.r C: 30; n.r	The 3^rd^ trimester of pregnancy (daily, 7 d)	Iron supplementation (i.e., ferrous fumarate tablet)	Pure (Tamer, Sukari)	3 dates/d (~40 gr/d) **→** 7 d, 21 dates, ~840 gr	Hb level (T0: baseline, T1: end of intervention time)	Sig ↑ at T1	Serious ^a^
Ma’mum et al., 2020 (Indonesia) [72]	2-arm non-RCT	G: primigravida& multigravida	n.r	E: 10; 28.40±n.r C: 10; 27.40±n.r	The 3^rd^ trimester of pregnancy (daily, 10 d)	Iron supplementation (i.e., ferrous sulfate tablet)	Juice (n.r)	n.r	Hb level (T0: baseline, T1: end of intervention time)	Sig ↑ at T1	Serious ^a^
Sugita & Kuswati, 2020 (Indonesia) [41]	2-arm non-RCT	G: primigravida& multigravida	n.r	E: 15; n.r C: 15; n.r	The 3^rd^ trimester of pregnancy (daily, 14 d)	Iron supplementation (i.e., ferrous sulfate tablet)	Pure (Tamer, n.r)	7 dates/d (~80 gr/d) **→** 14 d, 98 dates, ~1,120 gr	Hb level (T0: baseline, T1: end of intervention time)	N/S	Serious ^a^
Yuviska & Yuliasari, 2020 (Indonesia) [73]	2-arm non-RCT	n.r	n.r	E: 20; n.r C: 20; n.r	The 3^rd^ trimester of pregnancy (daily, 7 d)	Iron supplementation (i.e., ferrous sulfate tablet)	Juice (n.r)	3 tablespoons/d (~15 mL/d), before meal **→** 7 d, 21 tablespoons, ~105 mL	Hb level (T0: baseline, T1: end of intervention time)	Sig ↑ at T1	Serious ^a^
Azizah et al., 2023 (Indonesia) [32]	2-arm non-RCT	G: 1	36-37	E: 17; n.r C: 16; n.r	Late pregnancy (daily, from 36-37 w of gestation until the onset of labor)	Standard care	Pure (n.r, Ajwa)	7 dates/d (~80 gr/d) **→** ~1-4 w, ~49-196 dates, ~560-2,240 gr	Prolonged duration of the 1^st^, 2^nd^, and 3^rd^ labor stages; the need for labor induction; adequateness of uterine contractions	N/S	Serious ^a^
Wahyuni et al., 2023 (Indonesia) [74]	2-arm non-RCT	G: 1	>38	E: 20; n.r C: 20; n.r	Late pregnancy (daily, from 38-39 w of gestation until delivery)	Standard care	Pure (n.r)	7 dates/d (~80 gr/d) **→** ~3-4 w, ~147-196 dates, ~1,680-2,240 gr	The smoothness of breast milk production (1^st^ d post-delivery)	Sig ↑	Moderate ^a^
Hiba et al., 2022 (Pakistan) [75]	2-arm RCT	P: 0 G: 1	>35	E: 70; 23.8±5.9 C: 70; 25.6±5.2	Late pregnancy (daily, from 35-36 w of gestation until the onset of labor)	Standard care^1^	Pure (based on parturients’ desire)	7 dates/d (80 gr/d) **→** ~2-4 w, ~98-196 dates, ~1,120-2,240 gr	CD (upon admission); duration of the labor’s latent phase; duration of the 2^nd^ and 3^rd^ labor stages; spontaneous onset of labor; the need for labor induction and augmentation	Sig ↓	Low ^b^
									Duration of the 1^st^ labor stage; delivery mode	N/S	
Hipni et al., 2022 [60]; Megawati et al., 2022 (Indonesia) [61]	2-arm non-RCT	G: primigravida& multigravida	37	E: 30; n.r C: 30; n.r	Late pregnancy (daily, from 37 w of gestation), labor’s active phase (once)	Standard care	Juice (Rutab, n.r)	n.r **→** ~4 w	Duration of the labor’s active phase; duration of the 2^nd^ labor stage	Sig ↓	Serious ^a^
Iqbal et al., 2022 (Pakistan) [76]	2-arm RCT	G: 1	37-38	E: 55; n.r C: 55; n.r	Late pregnancy (daily, from 37-38 w of gestation until the onset of labor)	Standard care^1^	Pure (n.r)	6 dates/d (~70 gr/d), twice daily with 3 dates at each time (intervals: n.r) **→** ~3-4 w, ~126-168 dates, ~1,470-1,960 gr	Spontaneous onset of labor	Sig ↑	Some concern^b^
									The need for labor induction	Sig ↓	
									Good APGAR score (5 min)	N/S	
Sandhi &Dewi, 2022 (Indonesia) [77]	2-arm non-RCT	n.r	37-40	E: 16; n.r C: 16; n.r	Late pregnancy (daily, from 37-40 w of gestation until the onset of labor)	Standard care	Pure (n.r, Ajwa)	7 dates/d (~80 gr/d) **→** 2 w, ~98 dates, ~1,120 gr	Duration of the 1^st^ and 2^nd^ labor stages; total duration of labor	Sig ↓	Serious^a^
									Duration of the 3^rd^ labor stage	N/S	
Andriani, 2021 (Indonesia) [78]	2-arm non-RCT	P: <4 G: primigravida& multigravida	≥36	E: 30; 26.3±4.0 C: 30; 25.9±3.7	Late pregnancy (daily, from 36-38 w of gestation until delivery)	Standard care	Pure (n.r, Tunisia)	3 dates/d (~40 gr/d) in the morning **→** ~1-4 w, ~21-84 dates, ~280-1,120 gr	Bishop score	Sig ↑	Moderate^a^
									Duration of the 1^st^ labor stage	Sig ↓	
Astari & Dewi, 2019 (Indonesia) [79]	2-arm non-RCT	P: primipara, multipara	37-38	E: 15; n.r C: 15; n.r	Late pregnancy (daily, from 37-38 w of gestation until delivery)	Standard care	Pure (n.r, Sukari)	3 dates/d (~40 gr/d) in the morning **→** ~1-4 w, ~21-84 dates, ~280-1,120 gr	Duration of the 1^st^ labor stage	Sig ↓	Serious^a^
Kuswati & Handayani, 2019 (Indonesia) [39]	2-arm non-RCT	P: 0-2 G: 1-3	n.r	E: 30; 27.9±3.7 C: 30; 26.9±4.0	Late pregnancy (daily, from 37 w of gestation until delivery)	Standard care	Pure (n.r)	7-9 dates/d (100 gr/d) **→** ~1-5 w, ~49-315 dates, ~700-3,500 gr	The total duration of labor	Sig ↓	Moderate ^a^
									The need for labor induction; labor bleeding rate; delivery mood	N/S	
Astutti et al., 2018 (Indonesia) [80]	2-arm non-RCT	G: 1	37	E: 15; n.r C: 15; n.r	Late pregnancy (daily, from 37 w of gestation until delivery)	Standard care	Juice (n.r)	n.r **→** ~1-4 w	Duration of the 1^st^ labor stage	Sig ↓	Serious ^a^
Kordi et al., 2017 [57], 2014 [55], 2013 (Iran) [56]	2-arm RCT	P: 0 G: 1	37-38	E: 105; 23.5±3.6 (91; 23.5±3.6) C: 105; 23.5±3.7 (91; 23.5±3.7)	Late pregnancy (daily, from 37-38 w of gestation until the onset of labor pain)	Standard care^1^	Pure (Rutab, Bam Mazafati/Iran)	6-7 dates/d (70-75 gr/d) **→** ~3-4 w (19.0±3.5 d), 115.0±20.8 dates, 1,470-2,100 gr	CD (upon admission); Bishop score; spontaneous onset of labor	Sig ↑	High ^b^
									Duration of the labor’s active phase; duration of 2^nd^ and 3^rd^ labor stages; the need for labor induction; gestation length	Sig ↓	
									Delivery mode; neonatal birth weight	N/S	
Razali et al., 2017 (Malaysia) [28]	2-arm RCT	P: 0 G: 1	36	E: 77; 27.7±0.3 C: 77; 28.3±0.4	Late pregnancy (daily, from 36 w of gestation until the labor’s active phase)	Standard care^1^	Pure (Tamer, n.r)	7 dates/d (80 gr/d) **→** ~1-4 w, ~49-196 dates, ~560-2,240 gr	The need for labor augmentation	Sig ↓	Low ^b^
									CD (upon admission); duration of the labor’s latent and active phases; duration of the 2^nd^ and 3^rd^ labor stages; spontaneous onset of labor; gestation length; the need for labor induction; delivery mode; delivery bleeding rate; maternal Hb level (before and after delivery); APGAR score (5 min); admission rate to NICU; neonatal birth weight	N/S	
Rahayu et al., 2016 (Indonesia) [81]	2-arm non-RCT	n.r	37	E: 36/ n.r C: 36/ n.r	Late pregnancy (daily, from 37 w of gestation until the onset of labor)	Standard care	Pure (Tamer, n.r)	9 dates/d (~90 gr/d), thrice daily with 3 dates at each time (intervals: 8 h) **→** ~1-4 w, ~49-252 dates, ~630-2,520 gr	Duration of the 1^st^, 2^nd^, and 3^rd^ labor stages; PPH rate	Sig ↓	Serious ^a^
Suroso & Paryono, 2016 (Indonesia) [82]	2-arm non-RCT	P: 1	34	E: 15; n.r C: 15; n.r	Late pregnancy (daily, from 34 w of gestation until the onset of labor)	Standard care	Juice (n.r)	n.r **→** ~3-8 w	Duration of the 1^st^ labor stage; bleeding rate of the 1^st^ labor stage	Sig ↓	Serious ^a^
Karimian et al., 2015; Yousefy Jadidi et al., 2015 (Iran) [66]	2-arm RCT	P: 0	38	E: 52; 23.0±3.1 C: 54; 24.2±3.6	Late pregnancy (daily, from 38 w of gestation until the onset of labor pain)	Standard care^1^	Pure (Rutab, Bam Mazafati/Iran)	7 dates/d (~ 80 gr/d**) →** ~1-3 w, ~49-147 dates, ~560-1,680 gr	CD (upon admission); Bishop score; spontaneous onset of labor	Sig ↑	High^b^
									Duration of the labor’s active phase; the need for labor induction; gestation length	Sig ↓	
									Delivery mode; APGAR score (1 and 5 min); neonatal birth weight	N/S	
Al-Kuran et al., 2011 (Jordan) [31]	2-arm non-RCT	P: 0 G: 1	36	E: 69; n.r C: 45; n.r	Late pregnancy (daily, from 36 w of gestation until the onset of labor pain)	Standard care^1^	Pure (Tamer, n.r)	6 dates/d (60-67 gr/d) **→** 4 w, 168 dates, ~1,680-1,876 gr	CD (upon admission); spontaneous onset of labor	Sig ↑	Moderate^a^
									Duration of the labor’s latent phase; the need for labor induction/augmentation	Sig ↓	
									Duration of the labor’s active phase; duration of the 2^nd^ and 3^rd^ labor stages; gestation length; delivery mode	N/S	
Sohrabi et al., 2022, a [58]; Sohrabi et al., 2022, b (Iran) [59]	3-arm RCT	P: 0	37-42	E: 60; 23.8±5.9 C: 60; 25.6±5.2	Labor’s active phase (each 30-60 min, from a CD of 4 cm until a CD of 10 cm)	Standard care + placebo syrup^2^ (3 Saccharin tablets blended with 150 mL water)	Syrup (Tamer, Bam Mazafati/Iran)	6 dates (50 gr) mixed with 150 mL water **→** ~3-6 times, ~166.7±34.6 min, 124.4±32.9 mL with a maximum of 150 mL	Duration of the labor’s active phase; duration of the 2^nd^ and 3^rd^ labor stages	Sig ↓	High^b^
									The pain of different labor stages (T0: baseline-CD of 4 cm; T1: end of the labor’s active phase-CD of 10 cm; T2: end of the 2^nd^ labor stage; T3: end of the 3^rd^ labor stage)	Sig ↓ at T1, T2, T3	
Firdausi & Mukhlis, 2021 (Indonesia) [83]	2-arm non-RCT	P: multipara	n.r	E: 17; n.r C: 17; n.r	1^st^ labor stage (n.r)	Standard care	Pure (n.r, Sukari)	7 dates (~80 gr)	Duration of the 1^st^, 2^nd,^ and 3^rd^ labor stages; the total duration of labor	Sig ↓	Serious ^a^
Triananinsi et al., 2021 (Indonesia) [84]	2-arm non-RCT	G: 1	n.r	E: 20; n.r C: 20; n.r	1^st^ labor stage (once, after CD of 4 cm)	Standard care ^+^ drinking tea (600 mL)	Juice (produced by CV Amal Mulia Sejahtera/Indonesia)	6 tablespoons (88.8 mL)	Reduction in the duration of the 1^st^ labor stage (i.e., the smoothness of 1^st^ labor stage)	Sig ↑	Serious ^a^
Zaher et al., 2021 (Egypt) [34]	2-arm non-RCT	P: 0	37-40	E: 46; n.r C: 46; n.r	1^st^ labor stage (once, CD of 4 cm or less)	Standard care^1^	Pure (n.r)	7 dates (~80 gr)	CD (T0: baseline-upon admission; T1-T3: 2, 4, 6 h after baseline); uterine contractions frequency and intensity (T0: baseline-upon admission; T1-T3: 2, 4, 6 h after baseline); fetal head descent status (T0: baseline-upon admission; T1-T3: 2, 4, 6 h after baseline)	Sig ↑ at T1, T2, T3	Serious^a^
									Duration of the 1^s^, 2^nd^, and 3^rd^ labor stages	N/S	
Addini et al., 2020 (Indonesia) [33]	2-arm non-RCT	n.r	n.r	E: 16; n.r C: 16; n.r	1^st^ labor stage (once, n.r)	Standard care	Pure (n.r)	~10 dates (100 gr)	Duration of the 2^nd^ labor stage	N/S	Serious ^a^
Pongoh et al., 2020 (Indonesia) [36]	2-arm non-RCT	P: primipara, multipara	n.r	E: 16; n.r C: 16; n.r	Labor’s active phase (n.r)	Standard care^1^	Juice (n.r)	~10 dates (100 gr) blended with 200 mL water	The normal duration of the 1^st^ labor stage	N/S	Serious ^a^
Mutiah, 2019 (Indonesia) [35]	2-arm non-RCT	P: 1	n.r	E: 17; n.r C: 17; n.r	1^st^ labor stage (n.r)	Standard care	Juice (n.r)	n.r	The total duration of labor	N/S	Serious ^a^
Taavoni et al., 2019 [64]; Fathi & Amraei, 2019 [62]; Fathi et al., 2018 (Iran) [63]	3-arm RCT	P: 0	38-42	E: 32; 24.0±3.3 (40; 25.4±4.5) C: 32; 24.0±2.9 (40; 24.9±4.2)	Labor’s active phase (each 30-60 min, from a CD of 4 cm until a CD of 8 cm)	Standard care^3^	Syrup (Tamer, n.r)	6 dates (~50 gr) blended with 150 mL water **→** ~2-3 times, ~97.4±27.3 min	CD (T0: baseline-CD of 4 cm; T1-T2: 2 and 4 h after baseline)	Sig ↓ at T1, T2	High ^b^
									Duration of the labor’s active phase	Sig ↓	
									The pain of the labor’s active phase (T0: baseline-CD of 4 cm; T1-T5: 30, 60, 90, 120, 150 min after baseline)	Sig ↓ at T2-T5	
Ahmed et al., 2018 (Saudi Arabia) [29]	3-arm RCT	P: multipara G: 1	n.r	E: 32; n.r C: 31; n.r	Labor’s active phase (once, immediately before the onset of labor)	Standard care^1^	Pure (Rutab, Rotana/Saudi Arabia)	7 dates (~80 gr) followed by 250 mL of drinking water	Duration of the 1^st^ and 3^rd^ labor stages; meconium- or blood-stained liquor	Sig ↓	Some concern^b^
									APGAR score (5 min); normal fetal heart rate	Sig ↑	
									CD (T0: baseline-upon admission; T1-T4: 1, 2, 3, 4 h after baseline); duration of the 2^nd^ labor stage; delivery mode; labor bleeding rate; uterine contractions (frequency, intensity, regularity); APGAR score (1 min); normal fetal presentation	N/S	
									Labor pain severity	n.r	
Al-Dossari et al., 2017 (Saudi Arabia) [30]	2-arm non-RCT	P: 0 G: 1-5	≥37	E: 27; 22.9±4.0 C: 27; 24.8±5.1	1^st^ labor stage (once, before CD of 6 cm)	Standard care^4^	Pure (n.r)	7 dates (~80 gr) followed by 300 mL of drinking water	Duration of the 2^nd^ and 3^rd^ labor stages	Sig ↓	Moderate ^a^
									Duration of the 1^st^ labor stage; the total duration of labor; delivery mode; APGAR score (1 and 5 min)	N/S	
Jayanti, 2014 (Indonesia) [37]	2-arm non-RCT	G: 1	n.r	E: 10; n.r C: 10; n.r	1^st^ labor stage (n.r)	Standard care ^+^ drinking sugar water	Juice (n.r)	n.r	Duration of the labor’s active phase	N/S	Serious ^a^
Kordi et al., 2010 (Iran) [85]	3-arm RCT	P: 0	37-42	E: 30; 20.9±2.2 C: 30; 21.2±2.7	Labor’s active phase and 2^nd^ labor stage (twice: first, CD of 4 cm; second, CD of 4 cm until delivery with 30 min intervals)	Standard care ^+^ placebo syrup (10 Saccharin tablets blended with 200 mL water)	Syrup (n.r)	First: 92 gr of dates’ honey combined with 140 mL hot water; second: 40 gr of dates’ honey blended with 600 mL hot water, 60 mL at each 30 min **→** ~4 times, ~90.3±351.0 min, minimum 132 gr of dates’ honey mixed with 740 mL hot water	CD (the labor’s active phase-80 min after initiation of intervention)	Sig ↑	Low ^b^
									Duration of the labor’s active phase; duration of the 2^nd^ labor stage	Sig ↓	
									Delivery mode	N/S	
Niknami et al., 2023 (Iran) [86]	2-arm RCT	P: 0-4 G: 1-5	37-42	E: 48; 26.8±5.3 C: 45; 27.4±5.7	Postpartum (once, 2 h post-delivery)	Standard care^2^	Pure (Tamer, Bam Mazafati/Iran)	~10 dates (100 gr), within a maximum time of 2 h	PPH rate (on the 1^st^ 24 h post-delivery)	Sig ↓	Low ^b^
Syarif, 2022 (Indonesia) [87]	2-arm non-RCT	P: primipara, multipara	n.r	E: 15; n.r C: 15; n.r	Postpartum (daily, n.r)	Standard care	Juice (n.r)	n.r	The smoothness of breast milk production (end of intervention time)	Sig ↑	Serious ^a^
Agustina et al., 2021 (Indonesia) [88]	2-arm non-RCT	n.r	n.r	E: 15; n.r C: 15; n.r	Postpartum (daily, n.r)	Standard care	n.r	n.r	Breast milk quantity (T0: baseline; T1: end of intervention time)	Sig ↑	Serious ^a^
Modepeng et al., 2021 (Thailand) [49]	2-arm RCT	G: 1-3	n.r	E: 25; 26.4±5.3 C: 23; 25.4±5.2	Postpartum (daily, 28 d, starting at 30-90 d post-delivery)	Standard care	Pure (Tamer, Deglet Nour/ Tunisia)	10 dates/d (100 gr/d) **→** 4 w, 280 dates, 2,800 gr	Breast milk quantity (T0: baseline; T1: 2^nd^ w of intervention; T2: 4^th^ w of intervention)	Sig ↑ (changes T0-T1, T0-T2, T1-T2)	Some concern ^b^
									Infant weight gain (T0: baseline; T1: 4^th^ w of intervention)	N/S	
Ramadhani & Akbar, 2021 (Indonesia) [89]	2-arm non-RCT	n.r	n.r	E: 15; n.r C: 15; n.r	Postpartum (daily, 10 d, starting at 3-40 d post-delivery)	Standard care	Juice (n.r)	n.r	Breast milk quantity (T0: baseline; T1: 5^th^ d of intervention; T2: 10^th^ d of intervention)	Sig ↑ at T0-2	Serious ^a^
Prianti & Eryanti, 2020 (Indonesia) [90]	2-arm non-RCT	n.r	n.r	E: 15; n.r C: 15; n.r	Postpartum (daily, n.r)	Standard care	Juice (n.r)	1 cup/d in the morning (before or after meals)	The smoothness of breast milk production (end of intervention time)	Sig ↑	Serious ^a^
Aminah & Purwaningsih, 2019 (Indonesia) [91]	2-arm non-RCT	n.r	n.r	E: 16; n.r C: 16; n.r	Postpartum (daily, 7 d, starting at 1-40 d post-delivery)	Standard care ^+^ drinking Katuk leaves extract	Pure (n.r)	8 dates/d (100 gr/d) **→** 7 d, 56 dates, 700 gr	The smoothness of breast milk production (T0: baseline; T1: end of intervention time)	Sig ↑ at T1 ^††††^	Serious ^a^
Putriningtyas & Hidana, 2016 (Indonesia) [92]	2-arm non-RCT	n.r	37-42	E: 28; n.r C: 28; n.r	Postpartum (daily, 28 d, starting at 1-150 d post-delivery)	Standard care^1^^+^ drinking sweetened condensed milk	Juice (n.r)	45 mL/d **→** 28 d, 1,260 mL	Infant weight gain (end of intervention time)	Sig ↑	Moderate ^a^
Yadegari et al., 2016 (Iran) [38]	2-arm RCT	P: 0 G: 1	37-42	E: 45; 22.6±3.8 C: 45; 22.9±3.9	Postpartum (daily, 10 d, starting at 2 h post-delivery)	Standard care^5^	Pure (Rutab, Bam Mazafati/Iran)	First: 10 dates (100 gr) at 2 h post-delivery; second: ~9-10 dates/d (100 gr/d) from 2^nd^ to 10^th^ d post-delivery, during breakfast and within a maximum time of 2 h **→** 10 d, ~90-100 dates, 1,000 gr	PPH rate (from the 2^nd^ d until 10^th^ d post-delivery)	Sig ↓	High ^b^
									PPH rate (on the 1^st^ d post-delivery); PPH duration (during 10 d post-delivery)	N/S	
Sakka et al., 2014 (Egypt) [93]	3-arm RCT	P: primipara, multipara	37-40	E: 25; 24.8±3.9 C: 25; 25.2±5.1	Postpartum (daily, 3 d, starting at 1 d post-delivery)	Standard care	Pure (n.r)	30 dates/d (~300 gr/d), thrice daily with 10 dates at each time (intervals: n.r) **→** 3 d, 90 dates, ~900 gr	Breast milk quantity (the 3^rd^ d post-delivery), infant weight gain (T0: baseline; T1-T2: 3^rd^ and 7^th^ d of intervention)	Sig ↑	Some concern ^b^
									Infant weight gain (14^th^ d of intervention)	N/S	
Mojahed et al., 2012 (Iran) [94]	2-arm RCT	G: <5	37-42	E: 44; 26.7±4.9 C: 51; 24.6±5.1	Postpartum (once, immediately after placenta delivery)	Standard care^6^	Pure (Rutab, Bam Mazafati/Iran)	~10 dates (100 gr) followed by drinking hot water for a maximum of 10 min	PPH rate (during 2 h post-delivery)	Sig ↓	High ^b^
Khadem et al., 2007 (Iran) [95]	2-arm RCT	P: <5	38-42	E: 31; 24.4±3.9 C: 31; 25.0±4.8	Postpartum (once, immediately after placenta delivery)	Standard care^6^	Pure (Tamer, Deglet Nour)	~4-5 dates (50 gr)	PPH rate (end of 1 h post-delivery; during 3 h post-delivery)	Sig ↓	Some concern ^b^
									PPH rate (end of 2 h and 3 h post-delivery)	N/S	

*Abbreviations*: ~ Estimated, ↑ Higher, more, severe, or longer, ↓ Less or shorter, *APGAR score* Appearance, pulse, grimace, activity, and respiration, *C* Comparison arm, *CD* Cervical dilation, *C/S* Cesarean section delivery, *cm* Centimeters, *d* Day(s), *E* Experimental arm, *gr* Grams, *h* Hour(s), *Hb* Hemoglobin, *M* Mean, *min* Minute(s), *NICU* Neonatal intensive care unit, *N/S* Not significant, *n.r* Not reported, *PPH* Postpartum haemorrhage, *RCT* Randomized controlled trial, *RoB* Risk of bias, *SD* Standard deviation, *Sig.* Significantly, *W* Week(s)

Note 1: Studies are ordered considering the dates consumption time and their publication date

Note 2: The experimental group received routine care in all studies except the study of Zaher et al. (2021) [34, 36]

^†^Ripening stages of dates include: 1) Hababou (Hababouk): whitish-cream color develops within four weeks after pollination; 2) Kimri: greenish color with hard texture; 3) Khalal: yellowish color; 4) Bisir: color becomes yellow, purplish to reddish; 5) Rutab: fruit becomes more soften and sweeter; and 6) Tamer: dark brown color with a soft texture and highest sweetness[96]

^††^The outcomes were measured with vaginal examinations, Bishop scoring system (i.e., rating of five components including cervical dilatation, effacement, position, consistency, and fetal station), labor partograph, a 0-10 pain rating scale, haemometer, weighting postpartum blood pad, pictorial blood loss assessment chart (PBLAC), or observation.

^†††^Outcomes in the experimental arm compared to the comparison arm, except in the study of Aminah & Purwaningsih (2019) [91]

^††††^In the comparison arm compared to the experimental arm

^a^The Cochrane’s Risk of Bias (RoB) in Non-randomized Studies-of Interventions (ROBINS-I) tool: 1) Low: the study is considered to be at low RoB for all domains; 2) Moderate: the study is judged to be at low or moderate RoB for all domains; and 3) Serious: the study is assumed to be at serious RoB in at least one domain, but not at critical RoB in any domain.

^b^The revised Cochrane’s RoB tool for randomized trials (RoB 2): 1) Low: the study is believed to be at low RoB for all domains; 2) Some concern: the study is deemed to raise some concerns in at least one domain, but not to be at high RoB for any domain; and 3) High: the study is judged to be at high RoB in at least one domain, or have some concerns for multiple domains in a way that substantially lowers confidence

^1^Women were requested to abstain from dates consumption

^2^Women were excluded if they consumed dates during the study

^3^Women were permitted to drink routine non-sweet liquids (i.e., water or sugar-free tea)

^4^Infusion of intravenous lactate ringer from admission until the end of the fourth labor stage

^5^Women were allowed to consume dates less than 50 gr/d at the same time as the experimental arm

^6^Infusion of 10 units of intramuscular oxytocin or 20 units of oxytocin in 1000 mL 5% dextrose in water (with the normal saline solution) immediately after delivery.

Forty-three trials used a two-arm design; the remaining five had three arms. We extracted the data from the standard intervention and dates consumption groups for three three-arm studies that considered an extra group of alternative interventions, including saffron-honey syrup [58, 59], honey syrup [62–64], and fenugreek herbal tea [93]. The remaining two three-arm trials had the following groups: 1) DPF consumption alone, DPF consumption followed by drinking water, and control (standard care) [29], and 2) DPF syrup, placebo syrup (Saccharin tablets blended with water), and control (standard care) [85]. For these two trials, we compared the groups of DPF consumption with drinking water and DPF syrup to those of control and placebo syrup to be more consistent with other included studies conducted intervention during labor.

Thirty-six studies applied a multiple-time intervention for either 7-14 days during the third trimester of pregnancy (*n*= 8), 1-8 weeks in the late pregnancy (*n*= 15), 3-28 days in postpartum (*n*= 9), 90.3-166.7 minutes during labor (*n*= 3), or four weeks in the late pregnancy in combination with one-time intervention in childbirth (*n*= 1). Also, eight studies administered one intervention session during either labor (*n*= 5) or postpartum (*n*= 3). The remaining four studies conducted intervention during delivery but did not report the consumption frequency [35–37, 83]. The dates’ administration dosage was approximately 3-10 pieces/day (4-315 pieces in total), 40-100 grams/day (50-3,500 grams in total), 15 or 45 mL/day (88.8-1,260 mL in total). Concerning the consumed dates’ ripening stage and form, 14 trials used Tamer in either pure form (*n*= 12) or syrup (*n*= 2), and six administered Rutab in either pure form (*n*= 5) or juice (*n*= 1). Additionally, 27 studies used DPF in pure form (*n*= 14), juice (*n*= 12), or syrup (*n*= 1), but their ripening stage was unspecified. The remaining study did not report the ripening stage and form [88]. The dates’ varieties were reported in 22 publications; the most used was Bam Mazafati (*n*= 6), pursued by Sukari (*n*= 3) and Ajwa (*n*= 3).

### Pooled analyses of the study outcomes

#### Gestation length

**Fig. 1:**
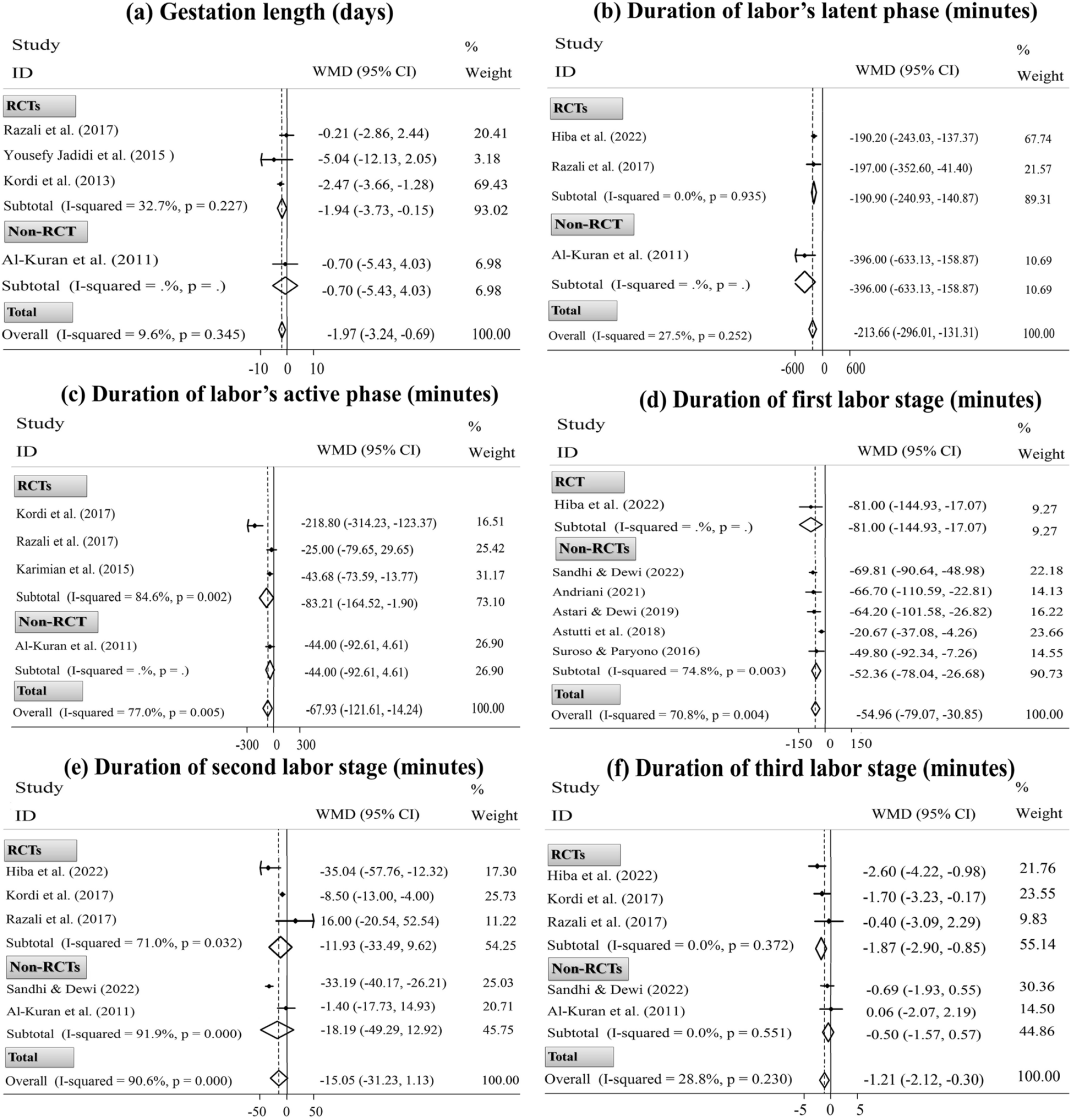
Forest plots for the effects of oral consumption of dates in late pregnancy on the duration of gestation (**a**), labor’s latent phase (**b**), labor’s active phase (**c**), the first labor stage (**d**), the second labor stage (**e**), and the third labor stage (f); stratified by study design (randomized controlled trial [RCT] vs. non-randomized controlled trial [non-RCT])

Four trials measured gestation duration following eating DPF in pure form during late pregnancy [28, 31, 56, 66]. Based on the meta-analysis, dates consumption had a low but significant effect on reducing gestation duration compared to the standard care (three RCTs and one non-RCT, WMD= ˗1.97 days; 95% CI [˗3.24 to ˗0.69 days];* P*= 0.003). After excluding only non-RCT [31], the overall estimation stayed significant for the remaining three RCTs (Fig. 1: a). Nevertheless, sensitivity analysis revealed the dependency of the overall pooled ES on the study by Kordi *et al.* [56] (WMD= ˗0.78 days; 95% CI [˗2.98 to 1.41 days]) (Supplementary Fig. 2: a).

#### Duration of different stages of labor

Twenty-eight publications addressed the labor duration in either the latent phase (*n*= 3), active phase (*n*= 11), the first stage (*n*= 14), the second stage (*n*= 16), or the third stage (*n*= 12), as well as in total (*n*= 5) [28–37, 39, 57, 59–63, 65, 75, 77–85]. Two publications had the same participants [62, 63]; hence, we excluded one with a smaller sample size from the meta-analysis [62]. Besides, the meta-analysis did not include eight other studies because they reported only categorical data (*n*= 6) [32, 33, 36, 37, 81, 84] or did not report the SDs (*n*= 2) [60, 61]. Alao, meta-analysis was not performed on two non-RCTs that reported the effectiveness of dates consumption during late pregnancy in reducing total labor duration [39, 77], considering the insufficient ESs. Hence, 17 trials were suitable for meta-analysis of labor duration.

The pooled analysis revealed that the consumption of PDF in late pregnancy compared to the standard care significantly reduced labor duration in the latent phase (two RCTs and one non-RCT, WMD= ˗213.66 min; 95% CI [˗296.01 to ˗131.31 min];* P*< 0.001), the active phase (three RCTs and one non-RCT, WMD= ˗67.93 min; 95% CI [˗121.61 to ˗14.24 min];* P*= 0.013), the first stage (one RCT and five non-RCTs, WMD= ˗54.96 min; 95% CI [˗79.07 to ˗30.85 min];* P*< 0.001), and the third stage (three RCTs and two non-RCTs, WMD= ˗1.21 min; 95% CI [˗2.12 to ˗0.30 min];* P*= 0.009); however, the intervention had a non-significant impact on reducing the duration of the second labor stage (three RCTs and two non-RCTs, WMD= ˗15.05 min; 95% CI [˗31.23 to 1.13 min];* P*= 0.068). After excluding only non-RCT [31], the primary findings on the duration of latent and active phases did not change. Similarly, the efficacy of the intervention was similar in the first stage duration, excluding only RCT [75]. Concerning the second stage duration, the finding was not dependent on the study design. However, RCTs [28, 57, 75] substantially affected the overall pooled ES of the third stage duration (Fig. 1: b-f). Likewise, after excluding the trial by Rezali *et al.* [28], sensitivity analysis altered the non-significant result of the primary meta-analysis obtained for the second labor stage duration to significant (four ESs, WMD= ˗18.97 min; 95% CI [˗35.99 to ˗1.94 min]). Also, the overall pooled ES of the active phase duration depended on the study by Karimian *et al.* [65] (WMD= ˗86.42 min; 95% CI [˗177.66 to 4.81 min]). Similarly, the result of the third labor stage duration depended on the study by Kordi *et al.* [57] (WMD= ˗1.03 min; 95% CI [˗2.21 to 0.14 min]) (Supplementary Fig. 2: b-f).

**Fig. 2:**
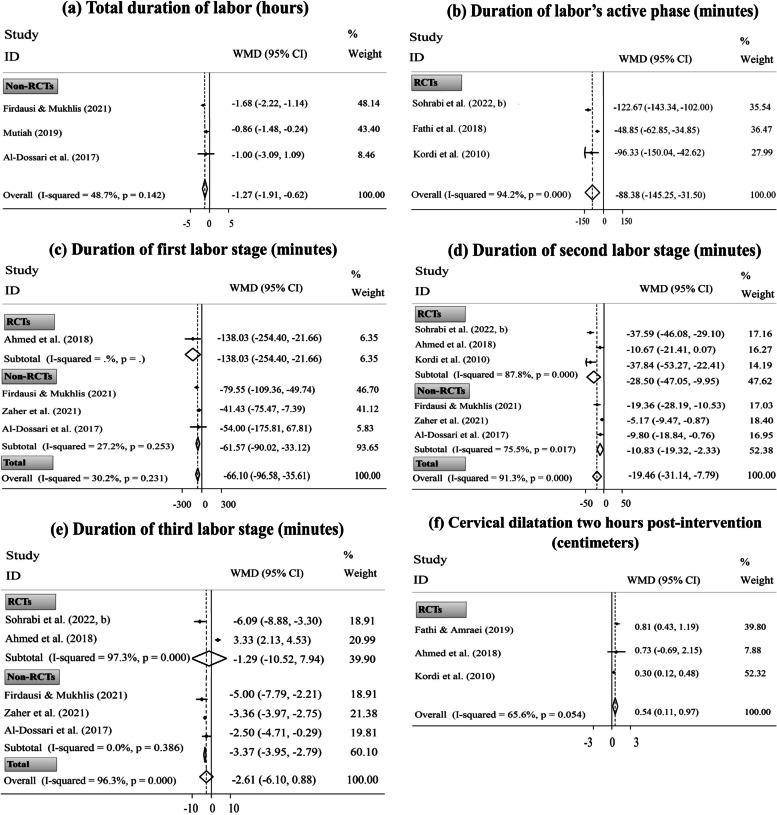
Forest plots for the effects of oral consumption of dates in labor on the duration of total labor (**a**), labor’s active phase (**b**), the first labor stage (**c**), the second labor stage (**d**), and the third labor stage (**e**); and cervical dilatation two hours post-intervention (**f**); stratified by study design (randomized controlled trial [RCT] vs. non-randomized controlled trial [non-RCT])

The meta-analysis also indicated that the consuming DPF in labor compared to the control conditions significantly reduced the total duration of labor (three non-RCTs, WMD= ˗1.27 hours; 95% CI [˗1.91 to ˗0.62 hours];* P*< 0.001) and labor duration in the active phase (three RCTs, WMD= ˗88.38 min; 95% CI [˗145.25 to ˗31.50 min];* P*= 0.002), the first stage (one RCT and three non-RCTs, WMD= ˗66.10 min; 95% CI [˗96.58 to ˗35.61 min];* P*< 0.001), and the second stage (three RCTs and three non-RCTs, WMD= ˗19.46 min; 95% CI [˗31.14 to ˗7.79 min];* P*= 0.001); nevertheless, the intervention had a non-significant influence on reducing the length of the third labor stage (two RCTs and three non-RCTs, WMD= ˗2.61 min; 95% CI [˗6.10 to 0.88 min];* P*= 0.068). After excluding only RCT [29], the intervention effect on the first stage duration remained substantial. Also, the overall pooled ES of the second stage duration was not dependent on the study design. However, the overall pooled ES of the third stage duration was substantially affected by RCTs because analysis of data based on non-RCTs altered the non-significant impact of the intervention to significant (Fig. 2: a-e). Similarly, after excluding the trial by Ahmed *et al.* [29], the intervention substantially affected the reduction of the third labor stage (four ESs, WMD= ˗3.83 min; 95% CI [˗5.10 to ˗2.57 min]). Yet, sensitivity analysis did not reveal the dependency of the overall pooled ESs obtained for other outcomes related to labor duration in a singular study (Supplementary Fig. 3: a-e).

#### Bishop score and CD

Fourteen publications reported the efficacy of consuming DPF on either Bishop score [65, 78], CD [28, 29, 31, 34, 56, 57, 62, 63, 75, 85], or both [55, 66]. Out of these, three collections of publications had similar participants; hence, their data were included in the meta-analysis once [55, 56, 62, 63, 65, 66].

**Fig. 3:**
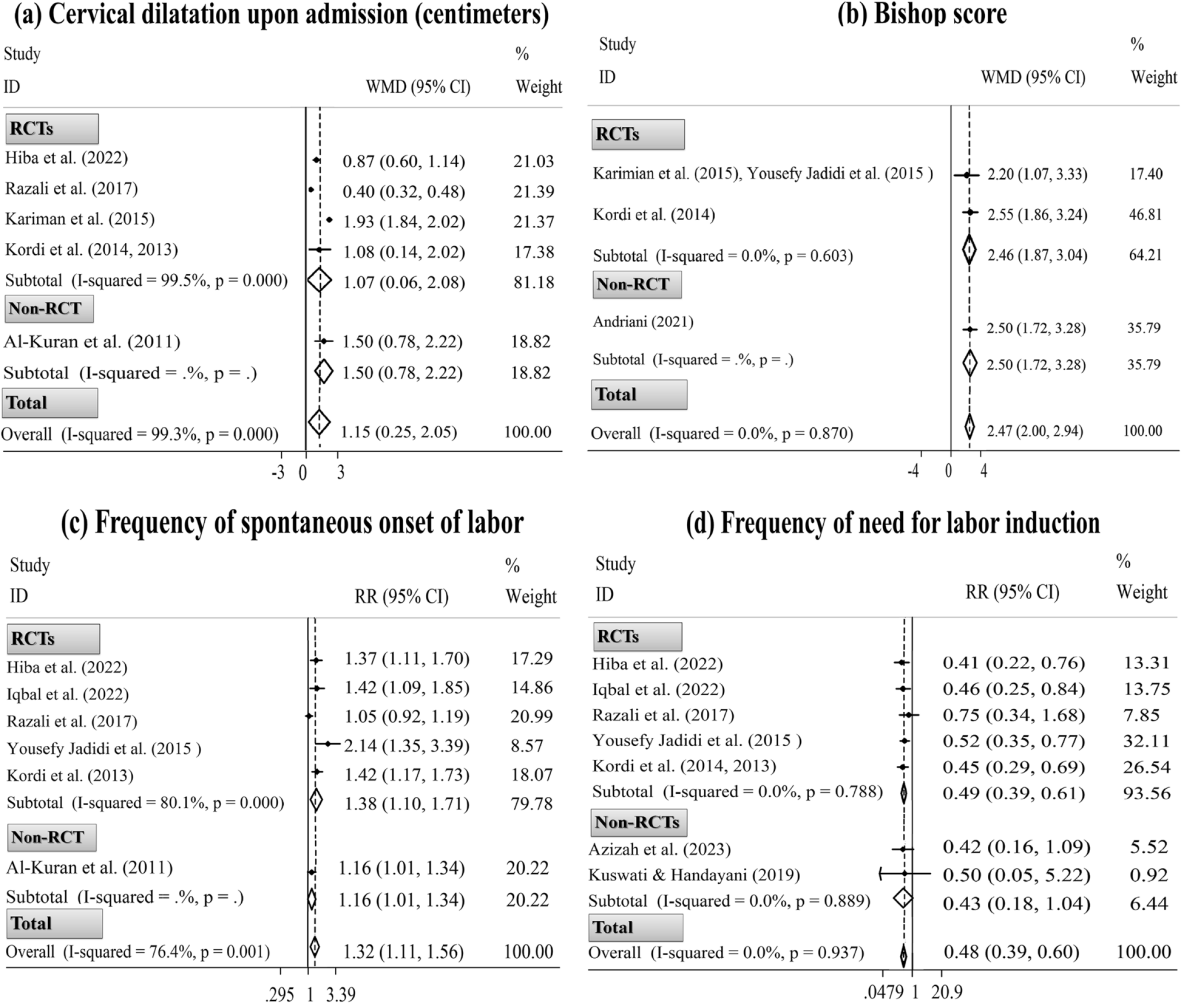
Forest plots for the effects of oral consumption of dates in late pregnancy on cervical dilatation upon admission (**a**); Bishop score (**b**); frequency of spontaneous onset of labor (**c**); and frequency of need for labor induction (**d**); stratified by study design (randomized controlled trial [RCT] vs. non-randomized controlled trial [non-RCT])

**Fig. 4:**
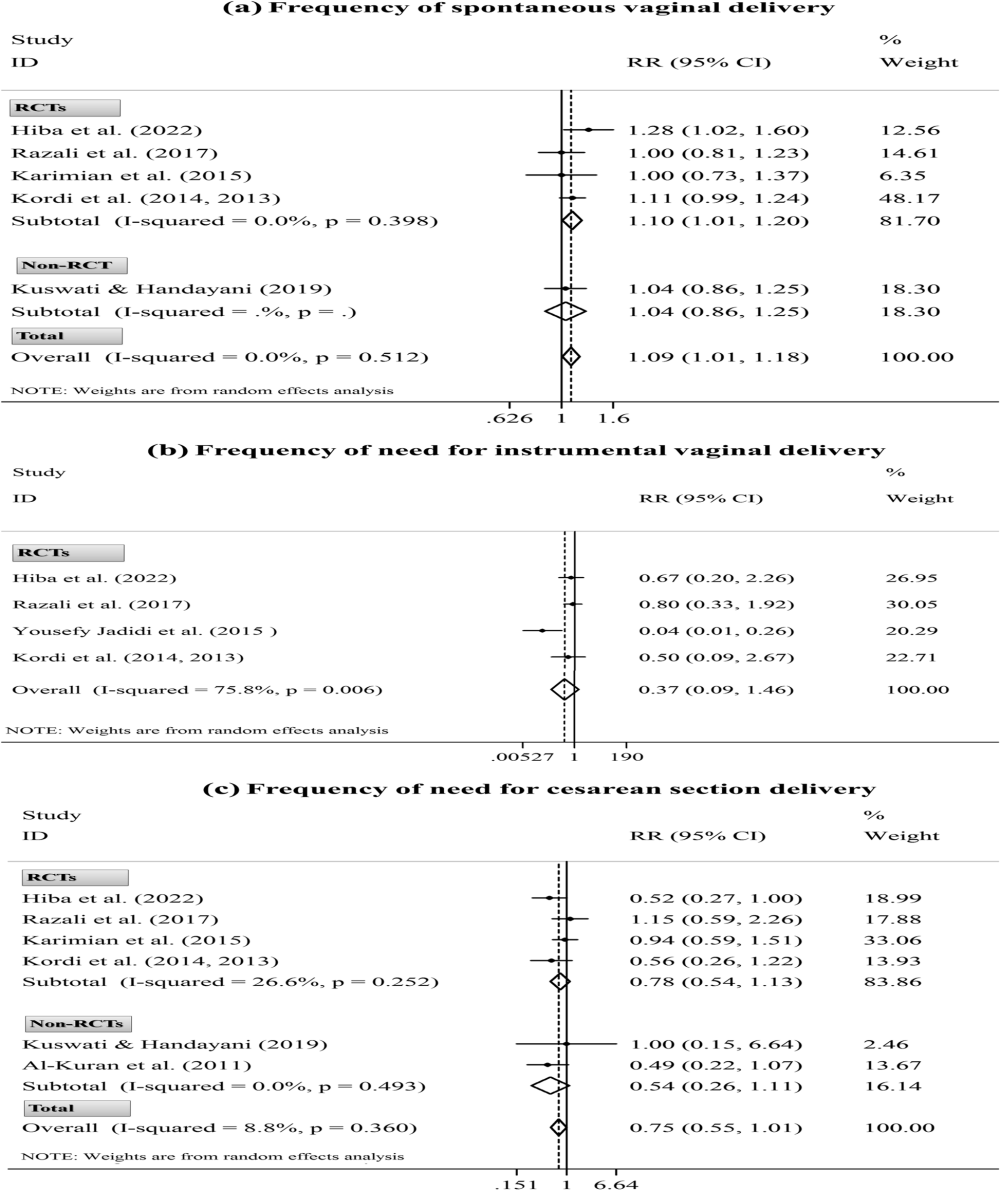
Forest plots for the effects of oral consumption of dates in late pregnancy on the frequency of spontaneous vaginal delivery (**a**), need for instrumental vaginal delivery (**b**), and need for cesarean section delivery (**c**); stratified by study design (randomized controlled trial [RCT] vs. non-randomized controlled trial [non-RCT])

Based on the pooled analysis, the CD significantly improved approximately two hours after the beginning of intervention in women who consumed DPF during labor compared with those in the standard/alternative care group (three RCTs, WMD= 0.54 cm; 95% CI [0.11 to 0.97 cm];* P*= 0.014) (Fig. 2: f). Moreover, eating DPF during late pregnancy, in comparison with the standard care, could significantly increase the CD upon admission (four RCTs and one non-RCT, WMD= 1.15 cm; 95% CI [0.25 to 2.05 cm];* P*= 0.012) and Bishop score (two RCTs and one non-RCT, WMD= 2.47; 95% CI [2.00, 2.94];* P*< 0.001). After excluding only non-RCT, the primary findings on the CD upon admission [31] and Bishop score [78] did not change (Fig. 3: a, b). Sensitivity analysis also did not reveal the dependency of the overall pooled ESs on a particular study (Supplementary Fig. 3: f & Fig. 4: a, b).

#### Status of labor onset

Ten publications reported the type of labor onset following consuming DPF in pure form during late pregnancy [28, 31, 32, 39, 55–57, 66, 75, 76]. Three of these were conducted on the same participants. Hence, one with a smaller sample size was excluded from the meta-analysis [57], and the remaining two publications were considered in the meta-analysis once [55, 56]. Three out of ten studies reported the significant effect of intervention in reducing the need for labor augmentation [28, 31, 75]. However, these studies were impossible to pool in the meta-analysis because one study reported the frequency of labor augmentation in combination with IOL [31].

The pooled analysis showed the significant effects of ingesting DPF in late pregnancy compared to the control conditions on increasing the frequency of spontaneous onset of labor (five RCTs and one non-RCT, RR= 1.32; 95% CI [1.11, 1.56];* P*= 0.001) and reducing the frequency of need for IOL (five RCTs and two non-RCTs, RR= 0.48; 95% CI [0.39, 0.60]; *P*< 0.001). Excluding only non-RCT [39] could not change the primary finding of spontaneous labor occurrence. Yet, the overall pooled ES of the need for IOL was substantially influenced by RCTs (Fig. 3: c, d). Based on the sensitivity analysis, the ESs of spontaneous onset of labor and IOL did not rely on an individual study (Supplementary Fig. 4: c, d).

#### Delivery mode

Two studies reported a non-significant effect of dates consumption during labor on delivery mode [30, 85]. Considering the insufficient ESs, these trials were not pooled in the meta-analysis. On the other hand, eight publications reported delivery mode after consuming DPF in late pregnancy [28, 31, 39, 55, 56, 65, 66, 75]. Out of these, two collections of publications had overlapping populations; hence, their data were included in the meta-analysis once [55, 56, 65, 66].

Pooled analysis disclosed that parturients who had consumed DPF in late pregnancy had a significantly more spontaneous vaginal delivery (four RCTs and one non-RCT, RR= 1.09; 95% CI [1.01, 1.18];* P*= 0.032); however, they had a non-significant lesser need for instrumental vaginal delivery (four RCTs, RR= 0.37; 95% CI [0.09, 1.46]; *P*= 0.154) and the C/S delivery (four RCTs and two non-RCTs, RR= 0.75; 95% CI [0.55, 1.01]; *P*= 0.054). After excluding only non-RCT [39], the primary finding on spontaneous vaginal delivery remained significant. Also, the study design could not affect the overall pooled ES of the C/S delivery (Fig. 4). Though, sensitivity analysis altered the non-significant effect of the intervention on C/S delivery to significant after excluding the studies by Rezali *et al.* [28] (five ESs, RR= 0.68; 95% CI [0.50, 0.93]) and Karimian *et al.* [65] (five ESs, RR= 0.66; 95% CI [0.46, 0.94]). Also, the overall ES of spontaneous vaginal delivery depended on the study by Hiba *et al.* [75] (four ESs, RR= 1.06; 95% CI [0.97, 1.15]) (Supplementary Fig. 5).

#### Breast milk production

Eight trials addressed the intervention efficacy in breast milk production, 1-90 days after delivery. Of these, four non-RCTs conducted in Indonesia evaluated the smoothness of breast milk production after the daily intake of DPF either in postpartum [87, 90, 91] or late pregnancy [74]. The remaining four trials (i.e., two RCTs and two non-RCTs) reported the breast milk quantity following the daily consumption of DPF during postpartum [49, 88, 89, 93].

**Fig. 5:**
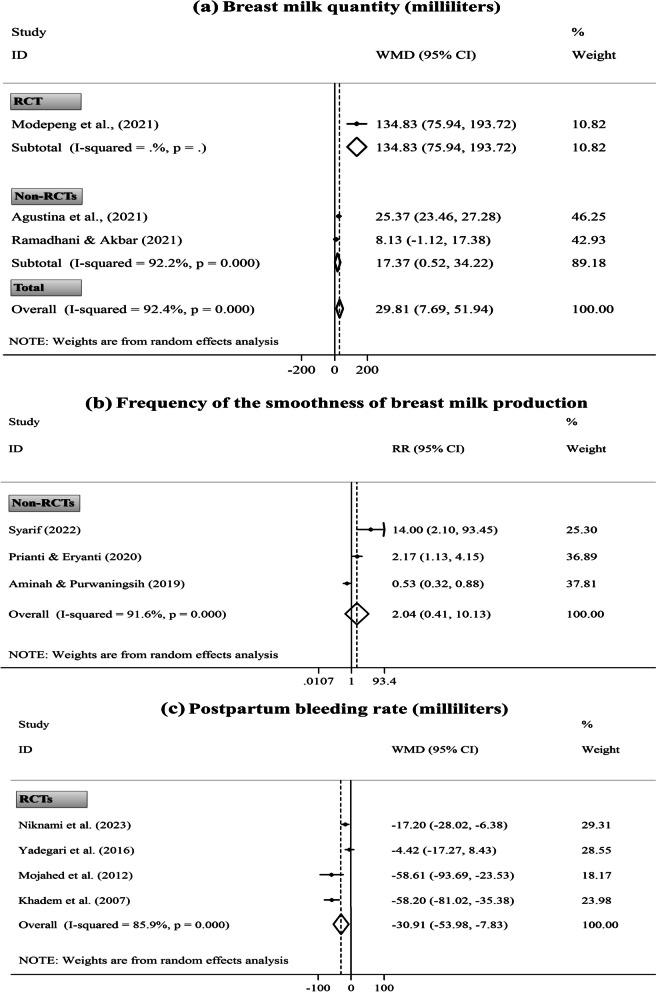
Forest plots for the effects of oral consumption of dates in postpartum on changes in breast milk quantity from baseline to post-intervention (**a**); the frequency of smoothness of breast milk production (**b**); and first-day postpartum bleeding rate (**c**); stratified by study design (randomized controlled trial [RCT] vs. non-randomized controlled trial [non-RCT])

Two of the eight studies were not incorporated in the meta-analysis due to methodological inconsistency with other studies [74, 93]. The pooled analysis showed the significant effects of the daily consumption of DPF during postpartum compared to the standard care on increasing changes in breast milk quantity from baseline to post-intervention (one RCT and two non-RCTs, WMD= 29.81 mL; 95% CI [7.69 to 51.94 mL];* P*= 0.008). However, intervention efficiency in boosting the smoothness of breast milk production was non-significant (three non-RCTs, RR= 2.04; 95% CI [0.41, 10.13];* P*= 0.381). After excluding only RCT [49], the primary finding of milk quantity did not change (Fig. 5: a, b). Also, excluding one study [91], which conducted an alternative intervention in the comparison group (i.e., drinking Katuk leaves extract), did not modify the primary finding of smoothness of milk production. Similarly, the sensitivity analysis did not show the dependency of the overall pooled ES obtained for the smoothness of milk production in a particular trial. However, the finding of breast milk quantity depended on the studies by Agustina *et al.* [88] (two ESs, WMD= 68.00 mL; 95% CI [˗55.97 to 191.98 mL]) and Ramadhani and Akbar [89] (two ESs, WMD= 75.98 mL; 95% CI [˗30.98 to 182.94 mL]) (Supplementary Fig. 6: a, b).

#### Bleeding rate

Four trials reported the labor bleeding rate after consuming DPF in late pregnancy [28, 39, 82] or labor’s active phase [29]. Since only two studies reported quantitative information [29, 82], data were unsuitable for the meta-analysis. However, none of these studies showed the potential effect of intervention in reducing the labor bleeding rate, except for one non-RCT [82].

Five trials also evaluated the postpartum bleeding rate following eating DPF in the postpartum [38, 86, 94, 95] or late pregnancy [81]. Of these, one non-RCT, which conducted an intervention in late pregnancy, was not incorporated in the meta-analysis [81]. Accordingly, ESs of the other four trials reported the bleeding rate during the first day after natural childbirth were pooled through the meta-analysis. The finding indicated the significant effect of eating DPF in pure form nearly after placenta delivery compared to the standard care (e.g., oxytocin injection) on reducing postpartum bleeding rate (four RCTs, WMD= ˗30.91 mL; 95% CI [˗53.98 to ˗7.83 mL]; *P*= 0.009) (Fig. 5: c). However, the sensitivity analysis exhibited the dependency of the overall pooled ES on the study by Niknami *et al.* [86] (three ESs, WMD= ˗38.75 mL; 95% CI [˗80.45 to 2.94 mL]) (Supplementary Fig. 6: c).

#### Maternal Hg levels

Eight non-RCTs in Indonesia evaluated Hb levels before and after the daily intake of DPF in pure or juice forms for 7-14 days in the third trimester of pregnancy [41, 67–73]. Additionally, one RCT showed no significant effect of the intervention in late pregnancy on maternal Hb status before and after delivery [28]; however, this was not incorporated in the meta-analysis, considering methodological inconsistency with the abovementioned studies.

**Fig. 6:**
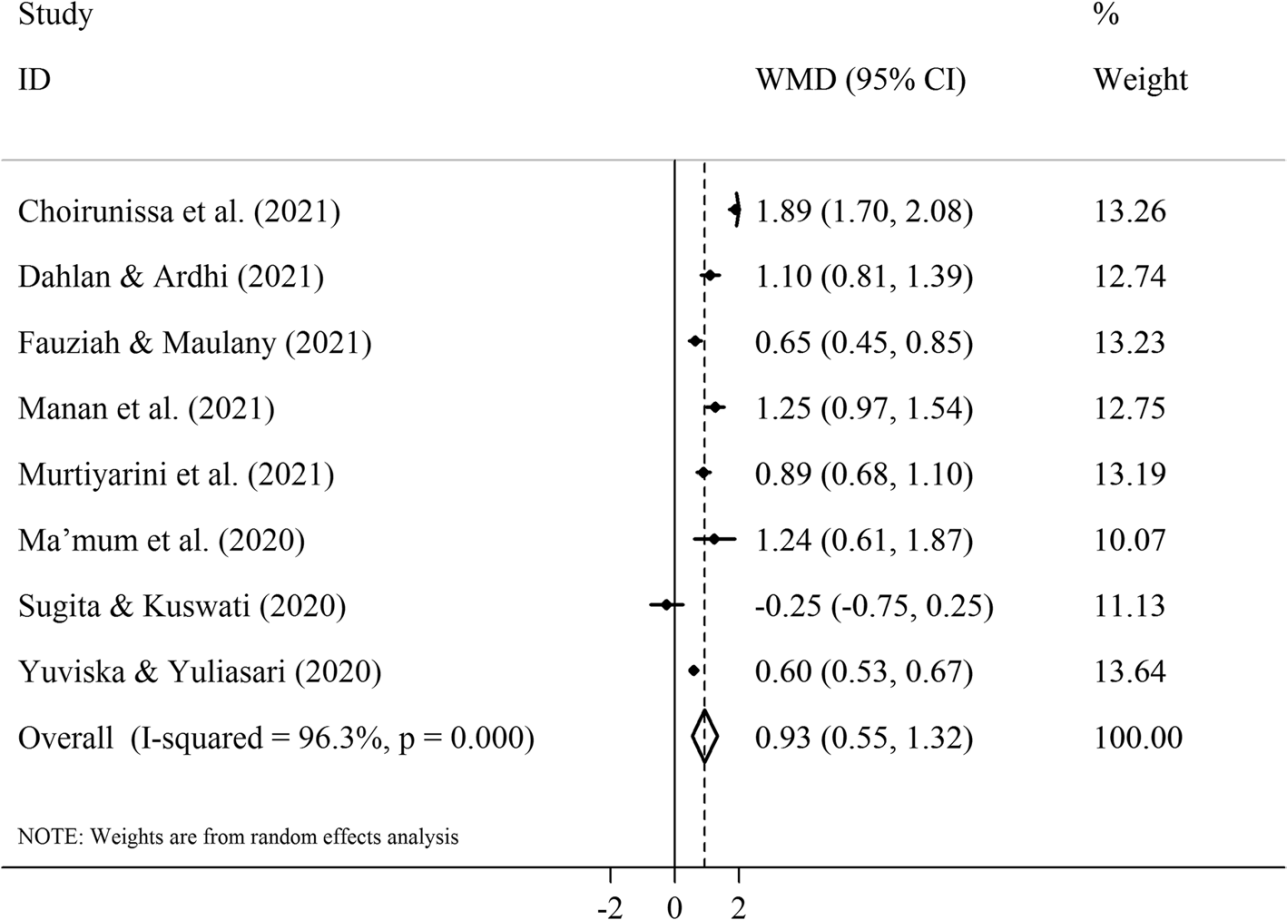
Forest plot for the effect of oral consumption of dates in the third trimester of pregnancy on changes in maternal hemoglobin levels (gr/dl) from baseline to post-intervention; all studies had a non-randomized design

According to the pooled analysis, supplementation with DPF and iron tablets in the third trimester of pregnancy led to more an increase in changes of Hb levels from baseline to post-intervention than consumption of iron tablets alone among parturients with mild/moderate pregnancy-related anemia (eight non-RCTs, WMD= 0.93 gr/dl; 95% CI [0.55 to 1.32 gr/dl];* P*< 0.001) (Fig. 6). The sensitivity analysis did not reveal the dependence of the overall pooled ES on an individual study (Supplementary Fig. 7).

#### Labor pain severity

Four publications evaluated the benefit of consuming DPF during the labor’s active phase on alleviating pain severity induced in the labor’s active phase alone [29, 62, 64] or labor’s active phase in addition to the second and third labor stages [58]. Two publications had the same participants [62, 64], and one did not report quantitative pain values and between-group differences [29]. Hence, performing a meta-analysis was impossible due to insufficient ESs. However, two RCTs showed a more significant reduction of labor pain after drinking DPF syrup during labor than the control conditions [58, 64].

#### Uterine contractions

Three studies evaluated the indices of uterine contractions (i.e., frequency, intensity, or regularity) after eating DPF in either late pregnancy [32] or labor [29, 34]. Two studies found no significant between-group differences [29, 32]. In contrast, the remaining one reported a significantly higher frequency and intensity of uterine contractions in women who consumed seven pieces of DPF in the first labor stage compared with those who only received standard care [34]. Running a meta-analysis was impossible due to data inconsistency.

#### Fetal, neonatal, or infant indices

Eight studies documented the fetal, neonatal, or infant outcomes, including the APGAR score (*n*= 5), neonatal birth weight (*n*= 3), infant weight gain (*n*= 3), fetal presentation (*n*= 2), fetal heart rate (*n*= 1), admission rate to neonatal intensive care ward (*n*= 1), and presence of meconium- or blood-stained liquor (*n*= 1) [28–30, 34, 56, 65, 76, 92]. Only two of five studies that measured the APGAR score displayed quantitative data [29, 65]. Similarly, two out of three studies reported quantitative values for birth weight [56, 65]. Also, we observed a methodological inconsistency among three studies on infant weight gain [49, 92, 93]. Hence, we could not pool the related data through meta-analysis. Nonetheless, most outcomes were not significantly different between dates consumer and standard care groups.

#### Adverse effects

Of four RCTs that addressed the adverse effects of intervention with consuming DPF, no side effects have been reported [28, 38, 55, 85].

### Subgroup and meta-regression analyses

There was no between-study heterogeneity for Bishop score (*I*^*2*^= 0.0%, Fig. 3: b), frequency of need for IOL (*I*^*2*^= 0.0%, Fig. 3: d), and frequency of spontaneous vaginal delivery (*I*^*2*^= 0.0%, Fig. 4: a). Also, between-study heterogeneity was low-to-moderate for gestation length (*I*^*2*^= 9.6%, Fig. 1: a), duration of labor’s latent phase (*I*^*2*^= 27.5%, Fig. 1: b), duration of third labor stage (intervention time: late pregnancy, *I*^*2*^= 28.8%, Fig. 1: f), total duration of labor (*I*^*2*^= 48.7%, Fig. 2: a), first labor stage duration (*I*^*2*^= 27.2%, Fig. 2: c), and frequency of need for C/S delivery (*I*^*2*^= 8.8%, Fig. 4: c). Nonetheless, high heterogeneity was discovered between studies for other study outcomes.

Based on the subgroup analysis, the study design might be a source of heterogeneity for the third labor stage duration (intervention time: labor, Fig. 2: e). In addition, this analysis suggested that the observed heterogeneity in the study outcomes could be due to differences in other variables, including the women’s gestational age at recruitment and their parity; the study’s country of origin, publication language, and methodological quality; the number of study arms; the comparison condition; and the dates’ administration form, ripening stage, and variety. Also, based on the subgroup results, some of the above variables were significantly associated with more changes in the study outcomes (Supplementary Table 4).

In addition to subgroup analysis, meta-regression was performed for continuous variables, including dates’ administration duration and dosage, as well as the study’s publication date and total sample size. According to the meta-regression, none of the mentioned variables was a heterogeneity source and was considerably associated with differences in the study outcomes, except for dates’ administration dosage, which had a significant association with changes in the length of the first and second labor stages (Supplementary Table 5).

### Dose-response analysis

Performing the dose-response analysis was suitable for labor duration, CD upon admission, and maternal Hb level. According to the results of this analysis, a significant inverse association was found between the changes in the duration of the second labor stage and the total consumption dosage of DPF when the intervention was performed during labor (*P-nonlinearity: consumed number*= 0.013, *consumed weight*= 0.016) (Supplementary Fig. 8). However, the link between the dates’ administration dosage and/or duration and differences in the length of labor in the first, second, and third stages was not dose-dependent when the intervention was accomplished in late pregnancy (Supplementary Figs. 9-11). Such a finding was also revealed for the third labor stage duration when the intervention was conducted during labor (Supplementary Fig. 12). Also, the relation between the consumption dosage and duration of DPF and the differences in the CD upon admission and maternal Hb level was not dose-dependent (Supplementary Figs. 13 and 14).

### Publication bias

Based on the results of Egger’s test, an asymmetry was disclosed for the pooled ESs of spontaneous onset of labor (*P*= 0.003) and the smoothness of breast milk production (*P*= 0.026). Nonetheless, applying the trim-and-fill technique could not modify the ESs of these outcomes, implying that publication bias did not influence the obtained results. Also, no publication bias was seen for other study outcomes according to Egger’s and Begg’s tests (Supplementary Table 6).

### The evidence quality and risk of bias

According to the Cochrane RoB2 tool, four RCTs had a low RoB for all criteria [28, 75, 85, 86]. However, other RCTs had unacceptable methodological quality, primarily due to concerns arising from the randomization process and high or unclear RoB in selecting the reported result (Supplementary Figs. 15, 16 & Supplementary Table 7). In addition, the overall quality of most non-RCTs was low based on the ROBINS-I, mainly due to high RoB in confounding, high or unclear RoB in the classification of interventions, and unclear RoB in the selection of the reported result (Supplementary Figs. 17, 18 & Supplementary Table 8). Likewise, the evidence quality was low or moderate in most outcomes based on the GRADE method. The leading reasons for diminishing the evidence rate were serious RoB and inconsistency (Supplementary Table 9).

## Discussion

Maternal health needs continuous effort as it could substantially affect society and the family’s health [97]. Commonly, parturients have used self-prescribed herbal remedies during peripartum, especially in low-income and upper-middle-income regions; however, the safety and effectiveness of this complementary intervention are still challenging [11]. Despite the widespread utilization of DPF as a natural supplement for its beneficial properties during peripartum, some potential drawbacks are reported regarding this caring approach [98]. Also, review studies supporting the effects of oral consumption of DPF on facilitating childbirth were mostly narrative or systematic, making an evidence-based conclusion impossible [23, 46, 47, 99–102]. Furthermore, previous meta-analyses have reported contradictory results regarding the potential effects of administrating DPF on perinatal outcomes [43–45]. Hence, the routine use of this practice in childbirth and perinatal care has remained questionable. Therefore, believing the highest rank of systematic reviews on the clinical evidence hierarchy [103], we conducted this updated systematic review with meta-analysis to augment the previous reviews regarding the safety of oral intake of DPF during the peripartum period and the efficacy of this integrated intervention in facilitating childbirth and improving perinatal outcomes.

Based on meta-analysis findings, administering DPF in postpartum increased breast milk production and reduced PPH more than routine interventions. Likewise, supplementation with DPF in the third trimester of gestation raised the parturients’ Hb level. A literature review showed no meta-analyses evaluating the efficacy of eating DPF on these outcomes. Nevertheless, the findings substantiated the earlier related systematic reviews. Two recent systematic reviews showed that DPF could increase the smoothness of breastfeeding and breastfeeding adequacy in postpartum mothers [104, 105]. Besides, in a systematic review of three RCTs, favorable evidence was documented for the usefulness of DPF in decreasing PPH [106]. Likewise, two reviews showed an effect of giving DPF on raising Hb levels in parturients with mild anemia [107, 108].

Based on the present meta-analysis, parturients with low-risk gestation consuming DPF in late pregnancy had significantly shorter latent and active phases and the first and third labor stages; nevertheless, they had a non-significant trend toward shortened the second labor stage. On the other hand, the administration of DPF during labor significantly reduced the labor length in the first and second stages and the active phase, while it had a non-significant impact on shortening the third labor stage. Based on the sensitivity analysis results, the non-significant findings could be due to the dependency of the overall estimate on an individual study. In other words, ignoring the RCT of Rezali *et al.* [28] changed the non-significant effect of consuming DPF during late pregnancy on declining the second labor stage length to significant. Similarly, excluding the RCT of Ahmed *et al.* [29], we found the intervention efficacy during labor concerning shortening the third labor stage duration.

The findings mentioned above updated the available systematic reviews that support the effect of DPF on minimizing childbirth duration [46, 47, 100]. However, previous meta-analyses have reported controversial results regarding labor duration. In a meta-analysis of three studies (i.e., one RCT, one quasi-RCT, and one non-RCT) published in English between 2011-2017, Sagi-Dain and Sagi indicated that women consuming DPF in late pregnancy had a significantly shorter latent phase (two ESs, MD= ˗275.56 min, *P*= 0.005) and the second labor stage (two ESs, MD= ˗7.66 min, *P*= 0.0005); however, they experienced no significant decline in time of active phase (three ESs, MD= ˗86.43 min, *P=* 0.06) and the third labor stage (three ESs, MD= ˗0.98 min, *P*= 0.09) [45]. The findings of the mentioned study are inconsistent with our results, except for shortening the latent phase duration. On the other, in a meta-analysis of five studies (i.e., four RCTs and one quasi-RCT) published in English and Persian until 2018, Bagherzadeh Karimi *et al.* demonstrated the significantly reducing effect of oral supplementation with DPF in late pregnancy and labor on the duration of active phase (three ESs, MD= ˗109.30 min, *P*= 0.01); nevertheless, the intervention had non-significant effects on shortening the first labor stage (two ESs, MD= ˗76.16 min, *P*= 0.22) and the second labor stage (four ESs, MD= ˗6.41 min, *P*= 0.44), as well as it did not reduce the third labor stage (three ESs, MD= 0.39 min, *P*= 0.82) [43]. The study described above evaluated the intervention efficacy during both late pregnancy and labor, which might lead to bias because their subgroup analyses based on the administration time during late pregnancy changed the significant effect of the intervention on reducing the active phase duration to non-significant (two ESs, MD= ˗125.39 min, *P*= 0.15). In contrast, it changed the non-impact of intervention on lowering the third stage duration to significant (two ESs, MD= ˗1.42 min, *P*= 0.03), which is consistent with the finding of the present study. In another meta-analysis of eight publications in Persian and English, Nasiri *et al.*, as the first attempt, showed that consuming DPF significantly shortened the first labor stage duration (five ESs, MD= ˗65.24 min, *P=* 0.009); yet, its effects were non-significant on diminishing the length of second labor stage (four ESs, MD= ˗11.27 min, *P=* 0.193) and the third labor stage (three ESs, MD= ˗0.98 min, *P=* 0.089) [44]. The study described above combined the data of studies that performed interventions during late pregnancy and labor; hence, it is impossible to compare this study’s findings with ours.

The discrepancies between the findings of the meta-analyses mentioned above and the current meta-analysis on the labor duration could be attributed to the number and design of included trials and different study objectives. In the present review, we obtained 15 RCTs and 38 non-RCTs published in English, Indonesian, and Persian until April 2023, using a comprehensive search of different data sources. Hence, more ESs were pooled for each study outcome compared to previous meta-analyses. Also, we analyzed data based on the intervention time as a leading confounding parameter. However, as mentioned earlier, only one of the previous meta-analyses considered the intervention time as a criterion for including studies [45], one another performed a subgroup analysis based on intervention time [43], and the remaining one overlooked intervention time as a variable for inclusion or subgroup analysis [44].

Based on the present meta-analysis, women consuming DPF in late pregnancy were experienced a significantly lower gestation length, admitted with a substantially higher CD, had a considerably higher Bishop score and spontaneous onset of labor, and encountered a significantly lower rate of IOL. Likewise, the intervention significantly increased the frequency of spontaneous vaginal delivery, while it did not significantly reduce the need for instrumental vaginal delivery and C/S. However, based on the sensitivity analysis, the non-significant effect of the intervention on the frequency of C/S changed to significant after excluding two trials (i.e., Rezali *et al.* [28] and Karimian *et al.* [65]). Similar to our findings, a meta-analysis showed that consuming DPF in late pregnancy significantly increased CD upon admission (three ESs, MD= 1.10 cm, *P=* 0.02) and decreased the need for IOL and/or augmentation (three ESs, RR= 0.60, *P=* 0.002); however, it had a non-significant impact on lowering the C/S rate (three ESs, RR= 0.70, *P=* 0.20) [45]. Another meta-analysis also demonstrated that DPF had a considerable effect on the progress of the Bishop score (two ESs, MD= 2.45, *P<* 0.00001*)*, yet it had a non-significant reducing effect regarding the C/S frequency (three ESs, RR= 0.80, *P=* 0.23) [43]. Besides, a meta-analysis showed that eating DPF significantly shortened gestation length (four ESs, MD= ˗0.30 days, *P<* 0.001) and increased CD on admission (five ESs, MD= 1.03 cm, *P=* 0.022) [44]. However, a meta-analysis of herbal drugs regarding the spontaneous onset of labor reported the non-significant effect of eating DPF in late pregnancy, using subgroup analysis (three ESs, RR= 1.05, *P=* 0.45) [14]. We pooled data from six studies on the spontaneous onset of labor; hence, the observed difference could be due to a higher number of pooled ESs in the current study.

### Implications for clinical practice and research

This meta-analysis revealed the usefulness of consuming DPF orally in the third trimester of pregnancy, late pregnancy, labor, or postpartum for the parturients or breastfeeding mothers. Our study suggests that using DPF had significant small-to-moderate effects on the reduction in the need for IOL and post-delivery bleeding; while at the same time improving the spontaneity of labor, the occurrence of spontaneous vaginal delivery, CD, Bishop score, breast milk volume, and maternal Hb levels. Also, the findings indicated that consuming DPF could significantly shorten gestation and labor, especially by the 213-minute shortening of the latent phase when considered in late pregnancy. Moreover, it can non-significantly decrease the frequency of instrumental vaginal delivery and C/S, as well as boost the smoothness of breast milk production. Accordingly, since consuming DPF is an easy, low-cost, non-pharmacological complementary intervention and DPF is readily obtainable in most regions and easily transportable, these effects are noteworthy in maternal-neonatal health nursing, especially in situations where little care might be available. However, there are some concerns about reaching a reliable conclusion on using this herbal remedy alone or in combination with other routine interventions for improving perinatal care.

The first concern is a lack of well-designed trials on the subject. Out of 48 studies, only four RCTs were deemed of excellent methodological quality. The subgroup analyses also indicated that the intervention was more efficacious in low-quality studies regarding increasing the CD upon admission and spontaneous onset of labor, as well as reducing the labor duration in the third and second stages. Moreover, the quality of evidence varied from low to moderate for most outcomes based on the GRADE approach. Accordingly, future trials with improved methodological quality should be conducted and reported rigorously based on the accepted guidelines. Since observing the effects of the intervention on fetal, neonatal, or infant indices as well as uterine contractions and labor pain severity was impossible using meta-analysis due to the restricted number of related trials, further investigations regarding these outcomes are deserved. Also, given the non-significant effects of the intervention on reducing the labor duration in the second and third stages following administrating DPF during late pregnancy and labor, respectively, as well as declining the frequency of instrumental vaginal and C/S deliveries, and boosting the smoothness of breast milk production, further studies on these outcomes are warranted. Moreover, since the included studies were conducted in Asia or the Middle East countries, where DPF is a staple in the daily regime, performing related trials in populations that do not regularly consume large amounts of DPF would provide more reliable information about how consumption of DPF can affect the study outcomes.

The second concern is the lack of a standard for dates’ administration dosage, duration, form, time, and cultivar to provide maximum results. Most manuscripts did not mention the dates’ variety and ripening stage. However, the chemical composites of different cultivars of dates could differ depending on climate, planting site, tree age, and fruit growth approach [21]. Also, the sweetness and ingredients of DPF in the Tamer and Rutab ripening stages are substantially different [96]. On the other hand, the included studies mainly examined the effectiveness of consuming 6-7 pieces of DPF per day (60-80 grams/day) in pure form during the last month of pregnancy. Previous meta-analyses suggested the consumption of DPF for 1-4 weeks during the late pregnancy, beginning from 36-38 weeks of pregnancy, as an intriguing option [43, 45]. Concerning the optimal intervention time, we found no significant impacts of the intervention on reducing the duration of the second labor stage following administrating DPF in late pregnancy. In contrast, the intervention during labor significantly reduced this stage’s length. Such conflicting findings were also observed for the duration of the third labor stage. Also, according to the subgroup analyses, we revealed that the intervention was more efficacious in the third labor stage duration (intervention time: late pregnancy), gestation length, CD upon admission, and spontaneous onset of labor in the studies conducted on women with a gestational age of more than or equal to 37 weeks, which might be due to more substantial number of included trials recruited women with this condition. Additionally, we found contradictory findings about the dates’ administration form. The intervention in late pregnancy remarkably declined the duration of the first labor stage in the investigations that administered DPF in pure form. However, the length of the first labor stage was more reduced after consuming DPF during labor in studies that used the pure form of DPF followed by drinking water. Also, the length of the second labor stage decreased more seriously when the juice form of DPF was administered during labor, whereas eating DPF in pure form might significantly increase maternal Hb levels. Regarding the dates’ administration variety, we found that gestation length was better reduced when Bam Mazafati was consumed.

Based on the dose-response estimations, the optimal amount and number of DPF to observe its maximum impact on lowering the length of the second labor stage were 200-700 grams and six pieces when administered during labor. However, we found no precise dosage or optimal duration of DPF for its impacts on other study outcomes. These findings could be due to the limited pooled ESs and low variations in the administration dosages and durations of DPF used in the included studies. Therefore, to reach evidence-based conclusions, further studies should evaluate the effects of oral intake of DPF during different periods, especially late pregnancy and labor, using different administration dosages, durations, forms, and varieties. Also, it is of merit to examine the impacts of consuming DPF at crucial periods, such as during prolonged labor and post-term pregnancies (i.e., beginning at 40 weeks of gestation), as well as among parturients with term pre-labor rupture of membranes. Additionally, it is suggested to evaluate the potential effect of DPF consumption before 37 weeks of gestation to induce preterm labor.

Another point of concern is the scarcity of safety data. Out of the 48 included studies, only four evaluated the adverse effects of the intervention. Although eating DPF was reported to be safe and free of side effects for the mother or the infant, the safety of this practice, especially in late pregnancy, is still questionable. In a review of the effectiveness of different botanical parts of dates, the authors declared treatment safety in obstetrics [109]. Nevertheless, the daily intake of DPF by parturients might be relatively troublesome, particularly in those with pre-gestational or gestational diabetes, due to the high amounts of sugar in DPF [45]. Besides, consuming more than two pieces of DPF at a time is assumed to substantially increase parturients’ blood glucose levels [98]. However, some studies documented that clients with diabetes can use DPF surely as it does not lead to immediate and meaningful changes in blood glucose; it may even benefit glycaemic and lipid control [110, 111]. Considering the controversial reports on adverse effects of DPF, it must be consumed in a specified dosage and duration and under the supervision of a high-qualified therapeutic team (e.g., maternity nurses, obstetricians, midwives, and dieticians). Also, forthcoming trials must measure laboratory parameters to ensure using DPF as a safe integrative care in the peripartum period.

### Strengths and novelty

The present review is the first dose-response meta-analysis performed to estimate the standard dosage and duration of DPF that must be administered to achieve ultimate results. Additionally, we investigated the effects of consuming DPF for the first time through a meta-analysis approach regarding the need for assisted vaginal delivery, frequency of spontaneous vaginal delivery, PPH, breast milk quantity and the smoothness of milk production, and maternal Hg level. Also, we used the last version of the Cochrane RoB tool for appraising the RCTs’ methodological quality; however, two previous meta-analyses utilized the old version of the Cochrane RoB assessment tool [43, 45], and one employed the Jadad scale [44]. Besides, we applied GRADE to assess the evidence quality, whereas only one previous meta-analysis used this approach regarding three included studies [45]. Unlike previous meta-analyses that combined the findings of studies with different designs (i.e., RCT, quasi-RCT, and non-RCT), we stratified data for RCTs and non-RCTs. Likewise, we categorized and analyzed data based on the consumption time of DPF, which was dismissed in two previous meta-analyses [43, 44]. Moreover, we summarized and statistically pooled the findings of all available studies published in any language, employing a broad search in diverse data sources.

### Limitations

First, in some outcomes, high heterogeneity was found in the pooled analyses, and the evidence quality was low or moderate, which could limit evidence-based conclusions. Second, only four studies recorded the adverse effects of intervention; therefore, the available data need to be expanded to reach valid conclusions about the safety of consuming DPF in peripartum. Third, some studies did not report the consumption dosages and durations of DPF. Although we requested further information from the studies’ authors, no reply was acquired in some cases; hence, estimations were made based on the researchers’ consensus. Fourth, performing a dose-response analysis was impossible in all issues because of the restricted number of included studies and the anonymous information on intervention dosages and durations. Fifth, given the limited pooled ESs, performing the meta-regression and subgroup analyses was sometimes impossible. Finally, due to the limited number of studies, we could not estimate the pooled effects of the intervention on fetal, neonatal, or infant indices, as well as uterine contractions and labor pain severity.

## Conclusion

This meta-analysis showed the benefits of eating DPF by parturients or breastfeeding mothers in the third trimester of pregnancy, late pregnancy, labor, or postpartum. The findings indicated that the administration of DPF could potentially shorten the duration of gestation and childbirth, decline the need for IOL, accelerate the spontaneity of delivery, raise CD and Bishop score, augment the frequency of spontaneous vaginal delivery, boost the breast milk quantity, reduce PPH, and improve maternal Hb levels. However, the intervention had non-significant but favorable impacts regarding the frequency of instrumental vaginal delivery, C/S, and the smoothness of breast milk production. Additionally, this study revealed a paucity of trials documenting the adverse effects of intervention with DPF and a scarcity of high-quality studies on the issue. Accordingly, to confirm the impacts of consuming DPF on childbirth and perinatal outcomes, additional studies with enhanced methodological quality are required to meticulously assess the adverse effects of intervention and estimate the safety laboratory indices. Moreover, exploring the exact dosage of DPF and the optimal intervention duration to obtain the maximum beneficial impacts on the study outcomes is of merit.

### Supplementary Information

**Additional file 1:** **Supplementary Fig. 1.** PRISMA 2020 flow diagram for the process of studies screening and selection. **Supplementary Fig. 2.** Sensitivity analysis for the effects of oral consumption of dates in late pregnancy on the duration of gestation (a), labor’s latent phase (b), labor’s active phase (c), the first labor stage (d), the second labor stage (e), and the third labor stage (f). **Supplementary Fig. 3.** Sensitivity analysis for the effects of oral consumption of dates in labor on the duration of total labor (a), labor’s active phase (b), the first labor stage (c), the second labor stage (d), and the third labor stage (e); and cervical dilatation approximately two hours after the beginning of intervention (f). **Supplementary Fig. 4.** Sensitivity analysis for the effects of oral consumption of dates in late pregnancy on cervical dilatation upon admission (a); Bishop score (b); frequency of spontaneous onset of labor (c); and frequency of need for labor induction (d). **Supplementary Fig. 5.** Sensitivity analysis for the effects of oral consumption of dates in late pregnancy on the frequency of spontaneous vaginal delivery (a), need for instrumental vaginal delivery (b), and need for cesarean section delivery (c). **Supplementary Fig. 6.** Sensitivity analysis for the effects of oral consumption of dates in postpartum on changes in breast milk quantity from baseline to post-intervention (a); the frequency of smoothness of breast milk production (b); and first-day postpartum bleeding rate (c). **Supplementary Fig. 7.** Sensitivity analysis for the effect of oral consumption of dates in the third trimester of pregnancy on changes in maternal hemoglobin levels (gr/dl) from baseline to post-intervention. **Supplementary Fig. 8.** Dose-response analysis for the association between the total administration dosage of dates and changes in the duration of the second labor stage (minute, intervention time: labor). **Supplementary Fig. 9.** Dose-response analysis for the association between the administration duration of dates and changes in the length of the first labor stage (minute, intervention time: late pregnancy). **Supplementary Fig. 10.** Dose-response analysis for the association between the administration dosage and duration of dates and changes in the length of the second labor stage (minute, intervention time: late pregnancy). **Supplementary Fig. 11.** Dose-response analysis for the association between the administration dosage and duration of dates and changes in the length of the third labor stage (minute, intervention time: late pregnancy). **Supplementary Fig. 12.** Dose-response analysis for the association between the administration dosage of dates and changes in the length of the third labor stage (minute, intervention time: labor). **Supplementary Fig. 13.** Dose-response analysis for the association between the administration dosage and duration of dates and changes in the cervical dilation upon admission (centimeters, intervention time: late pregnancy). **Supplementary Fig. 14.** Dose-response analysis for the association between the administration dosage and duration of dates and changes in the maternal hemoglobin levels (gr/dl, intervention time: the third trimester of pregnancy).**Supplementary Fig. 15.** Summary of the authors’ judgments about the risk of bias domains across the 15 included randomized controlled trials (RCTs). **Supplementary Fig. 16.** Summary of the authors’ judgments about the risk of bias domains within the 15 included randomized controlled trials (RCTs). **Supplementary Fig. 17.** Summary of the authors’ judgments about the risk of bias domains across the 33 included non-randomized controlled trials (non-RCTs). **Supplementary Fig. 18.** Summary of the authors’ judgments about the risk of bias domains within the 33 included non-randomized controlled trials (non-RCTs). **Supplementary Table 1.** PRISMA 2020 checklist for reporting systematic reviews and meta-analyses. **Supplementary Table 2.** Search characteristics in selected data-sources for the effects of oral consumption of dates in the peripartum period on childbirth and perinatal outcomes. **Supplementary Table 3.** Studies excluded after assessing their eligibility by full-text review (*n*= 33). **Supplementary Table 4.** Subgroup analyses for the effects of oral consumption of dates in the peripartum period on childbirth and perinatal outcomes. **Supplementary Table 5.** Meta-regression for the effects of oral consumption of dates in the peripartum period on childbirth and perinatal outcomes. **Supplementary Table 6.** Publication bias for the effects of oral consumption of dates in the peripartum period on childbirth and perinatal outcomes. **Supplementary Table 7.** Assessment of the risk of bias of the 15 included randomized controlled trials (RCTs) regarding the effects of oral consumption of dates in the peripartum period on childbirth and perinatal outcomes. **Supplementary Table 8.** Assessment of the risk of bias of the 33 included non-randomized controlled trials (non-RCTs) regarding the effects of oral consumption of dates in the peripartum period on childbirth and perinatal outcomes. **Supplementary Table 9.** GRADE evidence profile: the effects of oral consumption of dates in the peripartum period on childbirth and perinatal outcomes.

## Data Availability

Data and materials are available by contacting the corresponding author.

## Abbreviations

APGAR: Appearance, pulse, grimace, activity, and respiration
CD: Cervical dilatation
CI: Confidence interval
C/S: Cesarean section
DPF: Date palm fruit
ES: Effect sizes
GRADE: Grading of Recommendations Assessment, Development, and Evaluation Working Group
Hb: Hemoglobin
I^2^: I-squared statistic
ICTRP: International Clinical Trials Registry Platform
IOL: Induction of labor
IRCT: Iranian Registry of Clinical Trials
ISC: Scientific Content Database of the Islamic World Science Citation Center
MeSH: Medical subject headings
PPH: Postpartum hemorrhage
PROSPERO: International Prospective Register of Systematic Reviews
PRISMA: Preferred Reporting Items for Systematic Review and Meta-Analyses
RCTs: Randomized controlled trials
RoB: Risk of bias
ROBINS-I: Risk of bias in Non-randomized Studies-of Interventions
RR: Risk ratio
SD: Standard deviation
WMD: Weighted mean difference
