# Supplementary material for: Is oral consumption of dates (Phoenix dactylifera L. fruit) in the peripartum period effective and safe integrative care to facilitate childbirth and improve perinatal outcomes: a comprehensive revised systematic review and dose-response meta-analysis
Source: BMC Pregnancy Childbirth. 2024 Jan 2;24:12. doi: 10.1186/s12884-023-06196-y (PMC10759543; doi:10.1186/s12884-023-06196-y)
Supplement: Supplementary file 1 — Additional file 1: Supplementary Fig. 1. PRISMA 2020 flow diagram for the process of studies screening and selection. Supplementary Fig. 2. Sensitivity analysis for the effects of oral consumption of dates in late pregnancy on the duration of gestation (a), labor’s latent phase (b), labor’s active phase (c), the first labor stage (d), the second labor stage (e), and the third labor stage (f). Supplementary Fig. 3. Sensitivity analysis for the effects of oral consumption of dates in labor on the duration of total labor (a), labor’s active phase (b), the first labor stage (c), the second labor stage (d), and the third labor stage (e); and cervical dilatation approximately two hours after the beginning of intervention (f). Supplementary Fig. 4. Sensitivity analysis for the effects of oral consumption of dates in late pregnancy on cervical dilatation upon admission (a); Bishop score (b); frequency of spontaneous onset of labor (c); and frequency of need for labor induction (d). Supplementary Fig. 5. Sensitivity analysis for the effects of oral consumption of dates in late pregnancy on the frequency of spontaneous vaginal delivery (a), need for instrumental vaginal delivery (b), and need for cesarean section delivery (c). Supplementary Fig. 6. Sensitivity analysis for the effects of oral consumption of dates in postpartum on changes in breast milk quantity from baseline to post-intervention (a); the frequency of smoothness of breast milk production (b); and first-day postpartum bleeding rate (c). Supplementary Fig. 7. Sensitivity analysis for the effect of oral consumption of dates in the third trimester of pregnancy on changes in maternal hemoglobin levels (gr/dl) from baseline to post-intervention. Supplementary Fig. 8. Dose-response analysis for the association between the total administration dosage of dates and changes in the duration of the second labor stage (minute, intervention time: labor). Supplementary Fig. 9. Dose-response analysis for the association betwee [file 12884_2023_6196_MOESM1_ESM.docx]

**
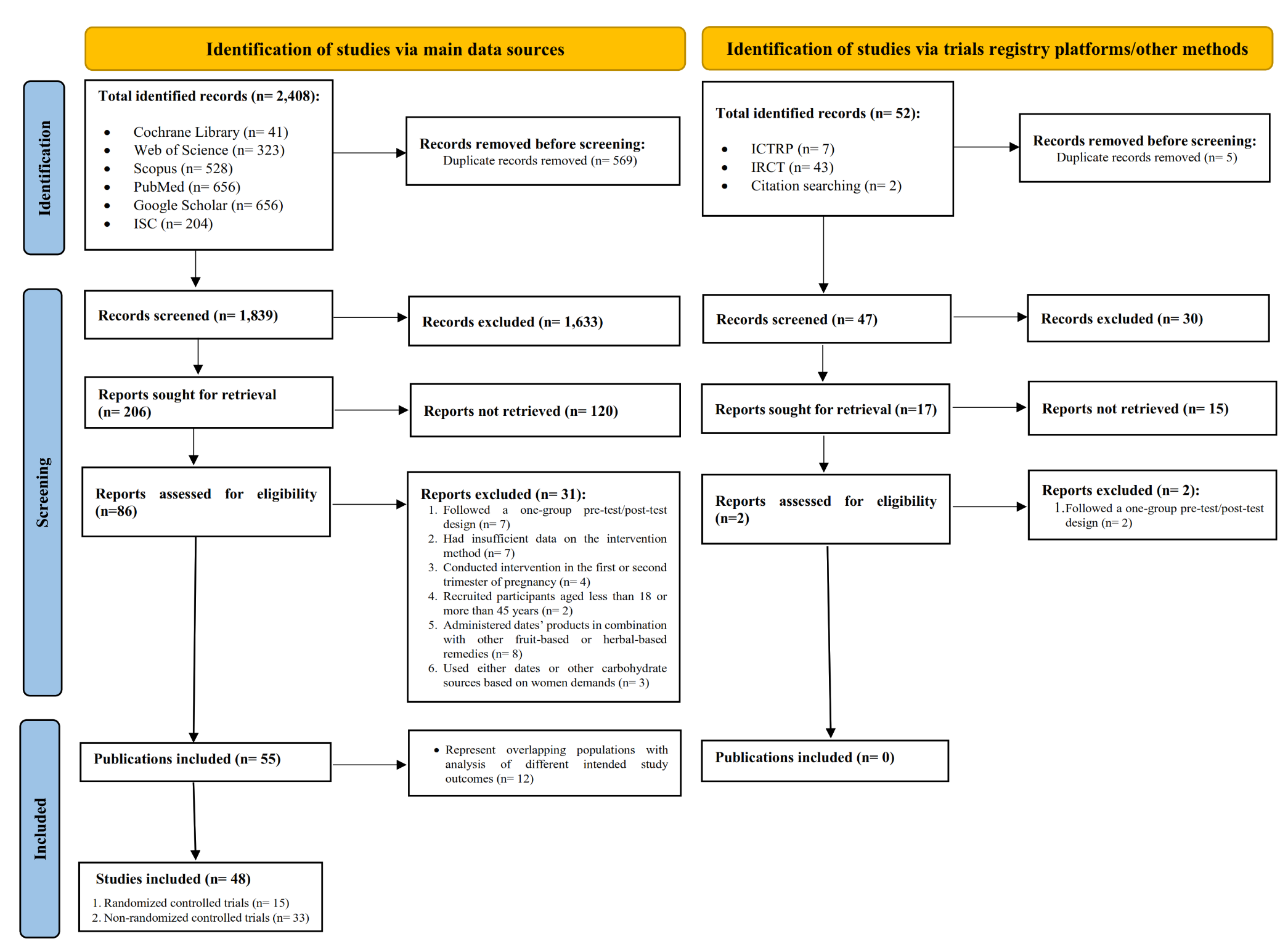
**

**Supplementary Fig. 1:** PRISMA 2020 flow diagram for the process of studies screening and selection

**
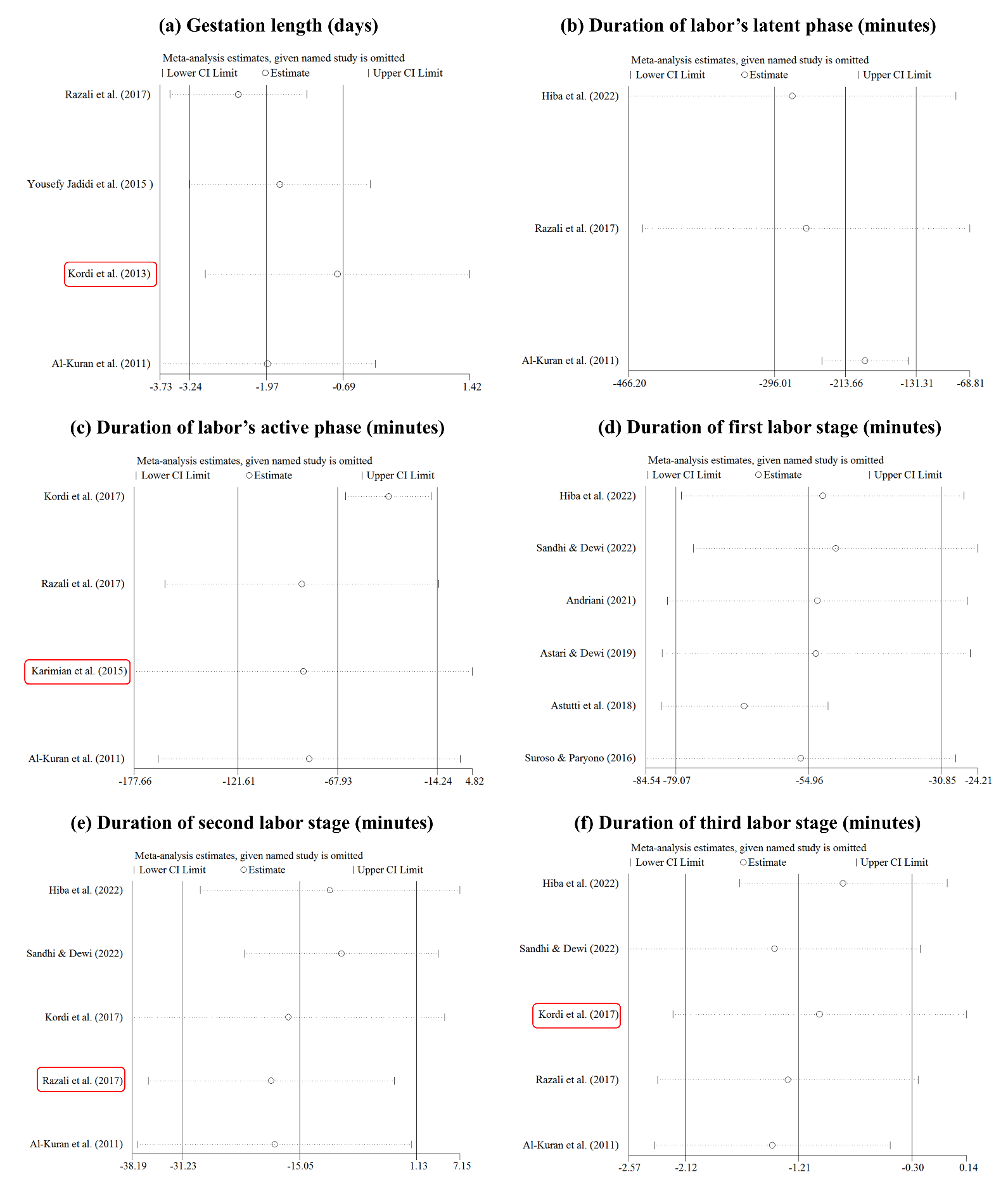
Supplementary Fig. 2:** Sensitivity analysis for the effects of oral consumption of dates in late pregnancy on the duration of gestation (a), labor’s latent phase (b), labor’s active phase (c), the first labor stage (d), the second labor stage (e), and the third labor stage (f)

**
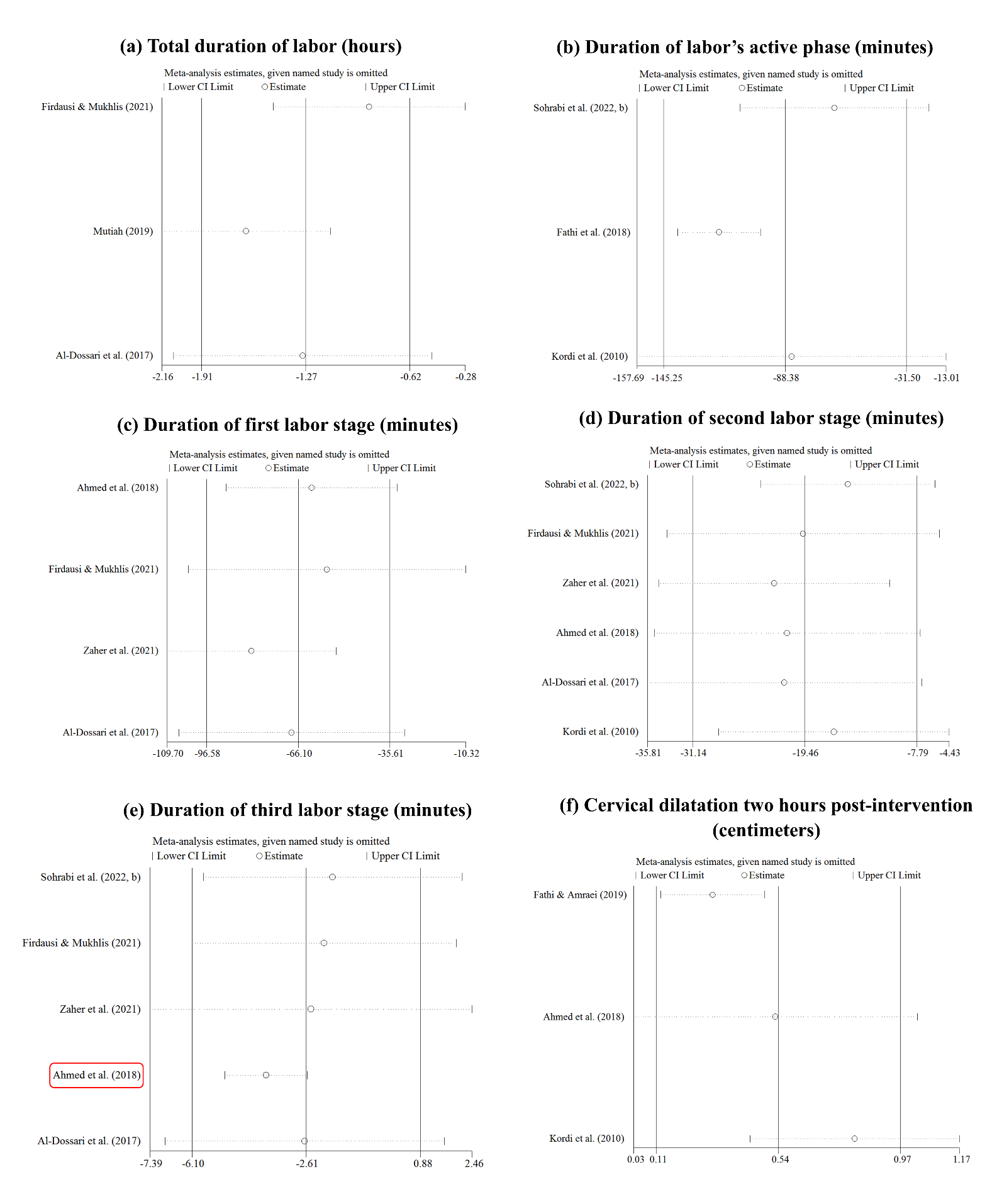
Supplementary Fig. 3:** Sensitivity analysis for the effects of oral consumption of dates in labor on the duration of total labor (a), labor’s active phase (b), the first labor stage (c), the second labor stage (d), and the third labor stage (e); and cervical dilatation approximately two hours after the beginning of intervention (f)

**
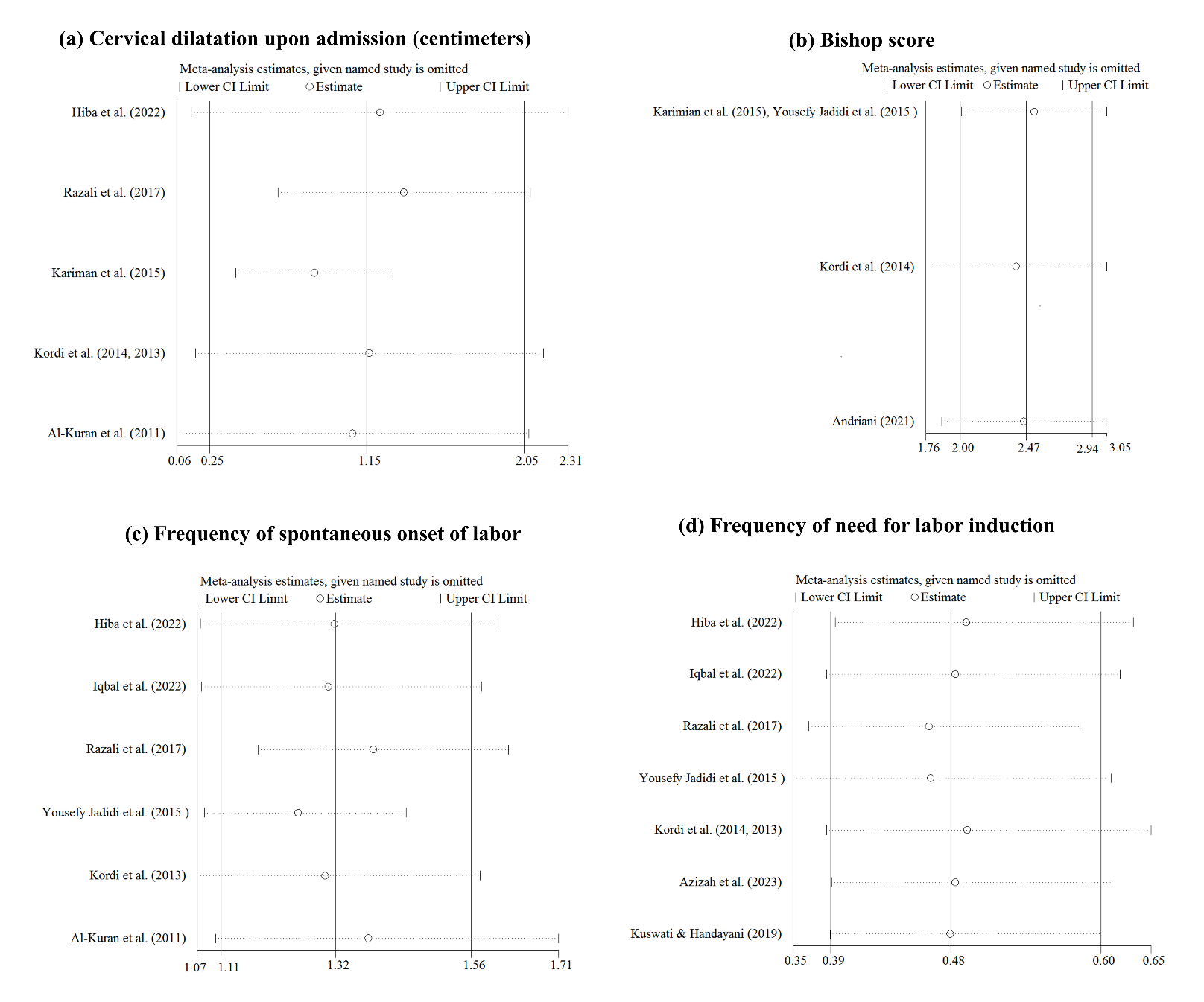
Supplementary Fig. 4:** Sensitivity analysis for the effects of oral consumption of dates in late pregnancy on cervical dilatation upon admission (a); Bishop score (b); frequency of spontaneous onset of labor (c); and frequency of need for labor induction (d)

**
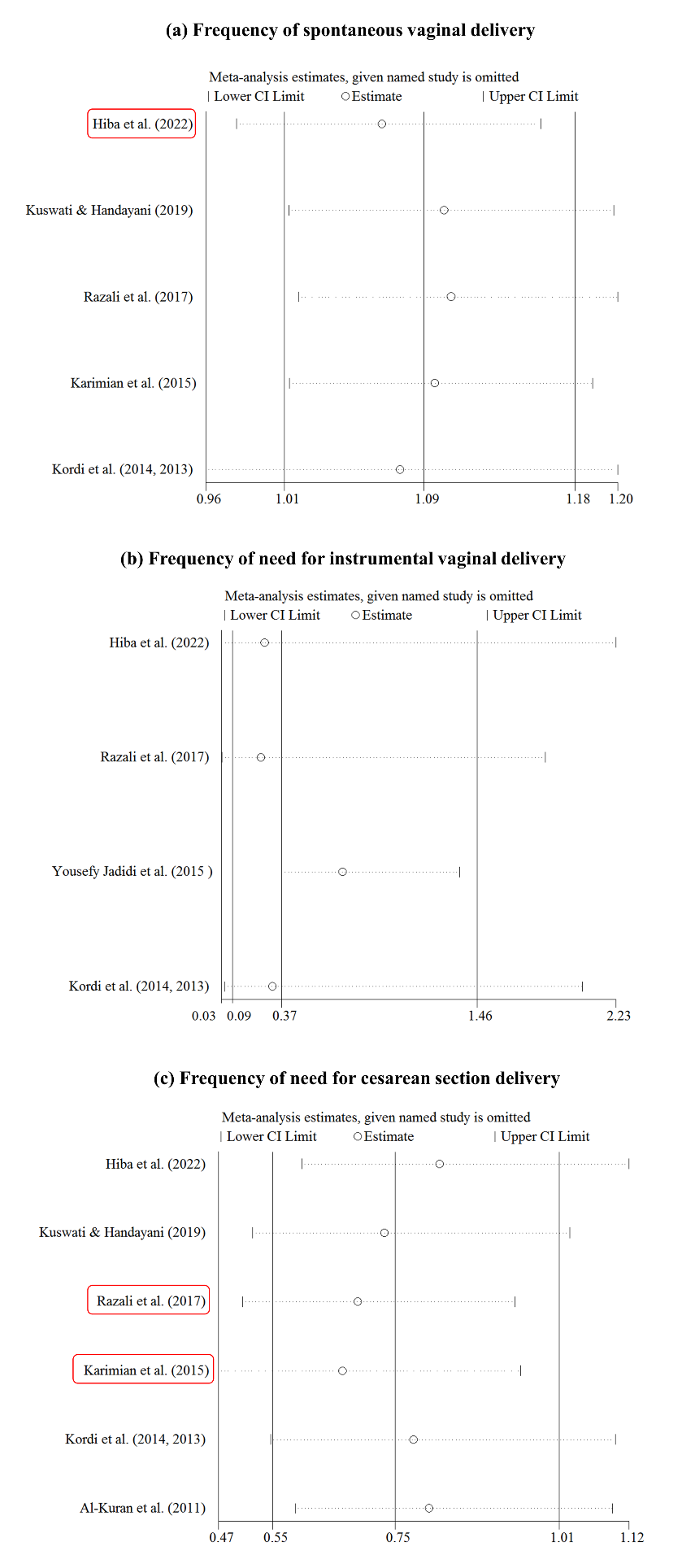
**

**Supplementary Fig. 5:** Sensitivity analysis for the effects of oral consumption of dates in late pregnancy on the frequency of spontaneous vaginal delivery (a), need for instrumental vaginal delivery (b), and need for cesarean section delivery (c)

**
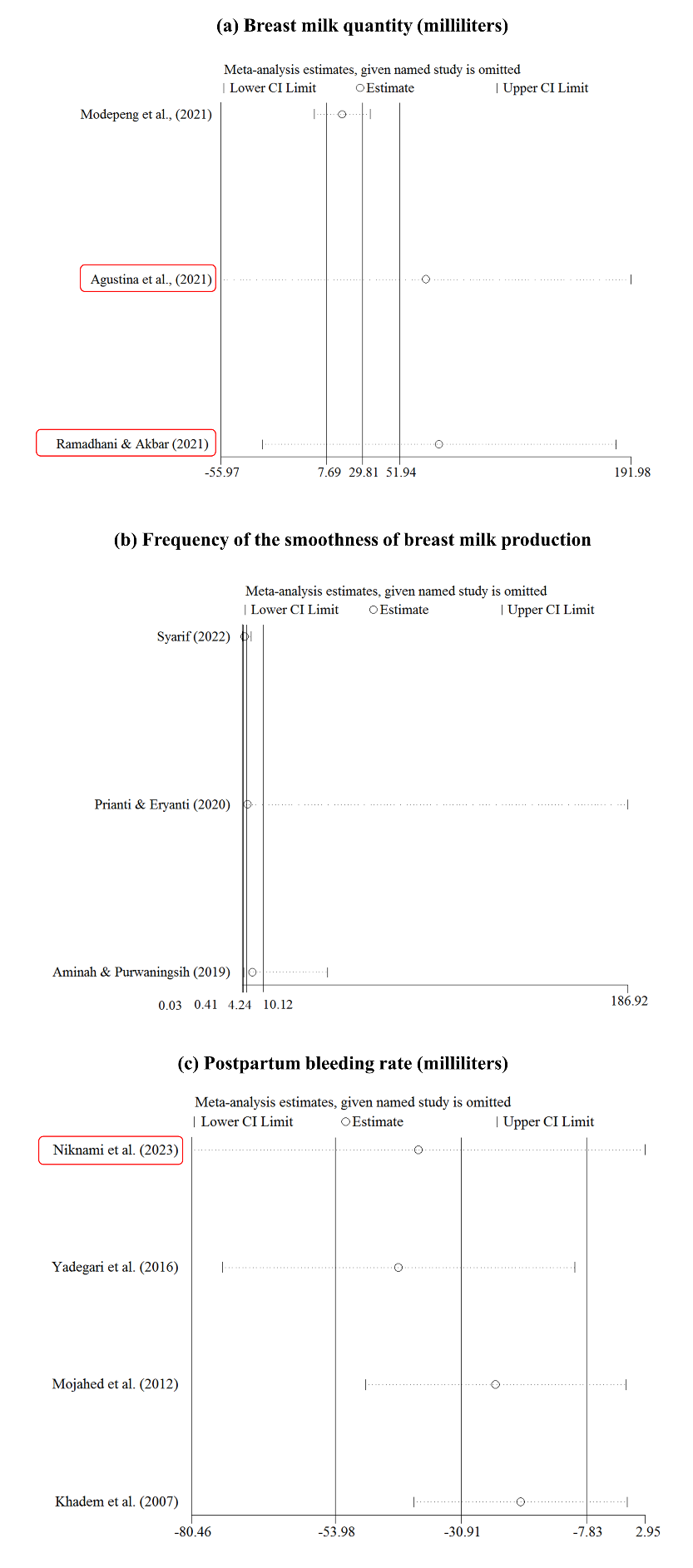
**

**Supplementary Fig. 6:** Sensitivity analysis for the effects of oral consumption of dates in postpartum on changes in breast milk quantity from baseline to post-intervention (a); the frequency of smoothness of breast milk production (b); and first-day postpartum bleeding rate (c)

**
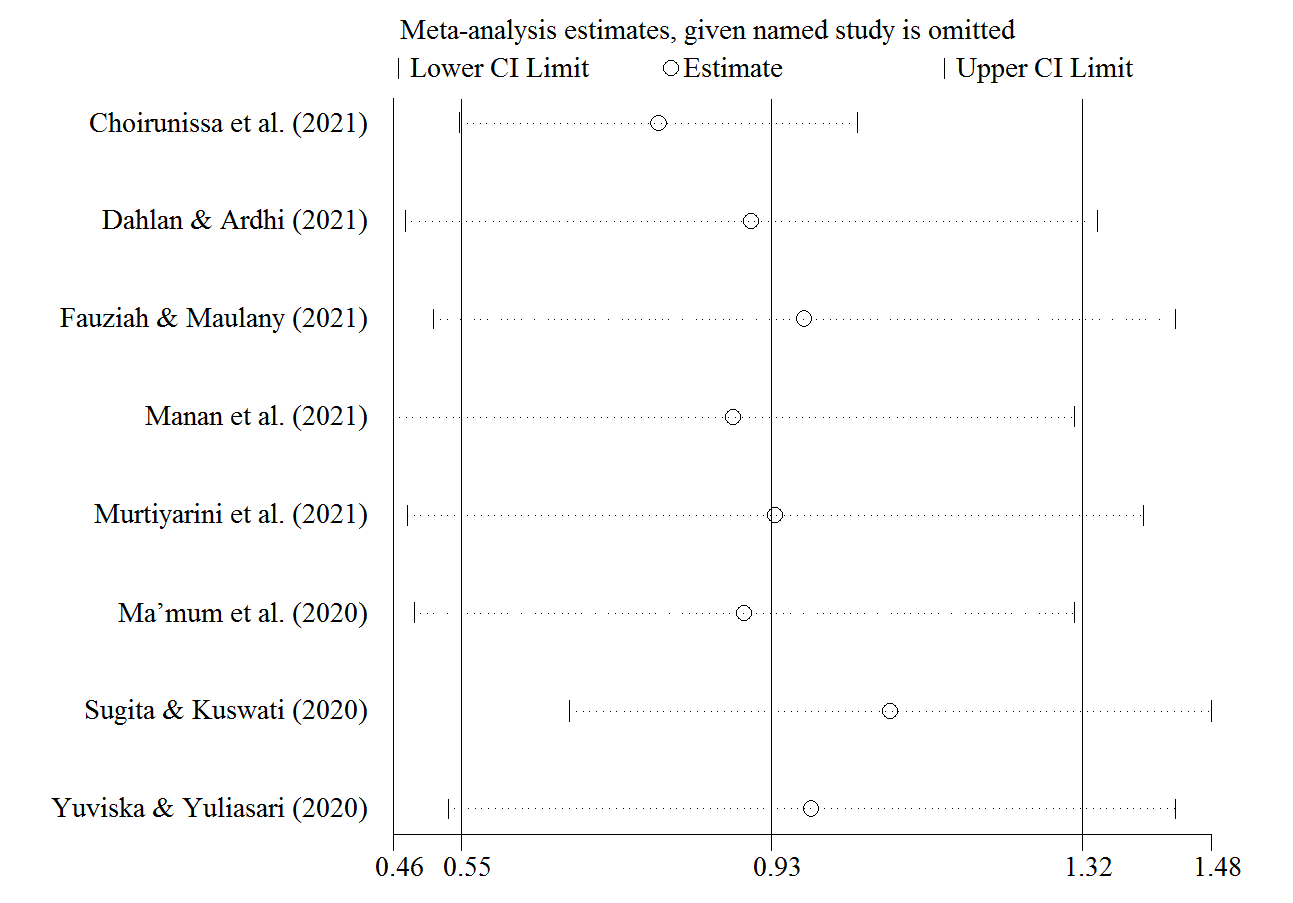
Supplementary Fig. 7:** Sensitivity analysis for the effect of oral consumption of dates in the third trimester of pregnancy on changes in maternal hemoglobin levels (gr/dl) from baseline to post-intervention

**
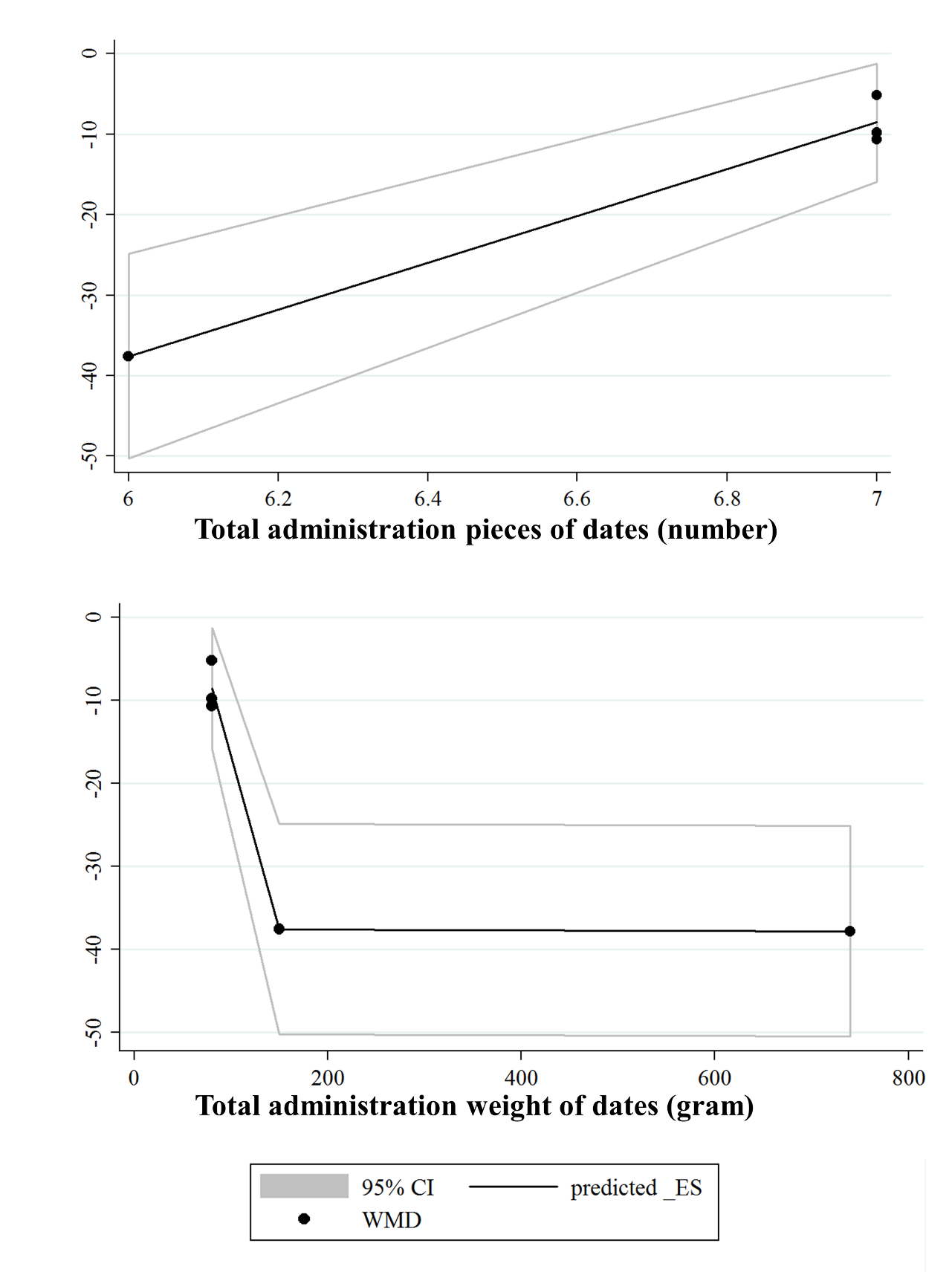
 Supplementary Fig. 8:** Dose-response analysis for the association between the total administration dosage of dates and changes in the duration of the second labor stage (minute, intervention time: labor)

**
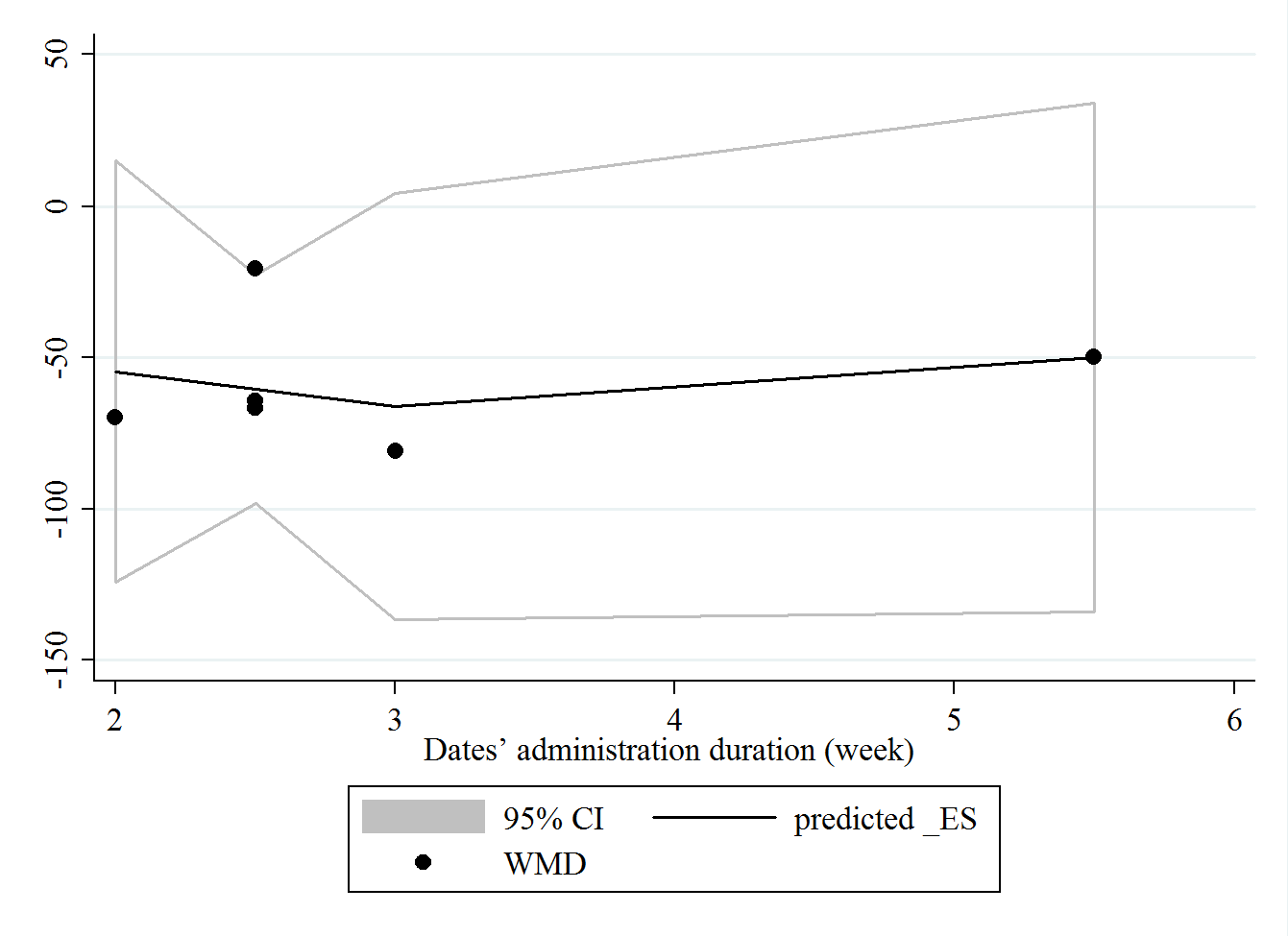
Supplementary Fig. 9:** Dose-response analysis for the association between the administration duration of dates and changes in the length of the first labor stage (minute, intervention time: late pregnancy)

**
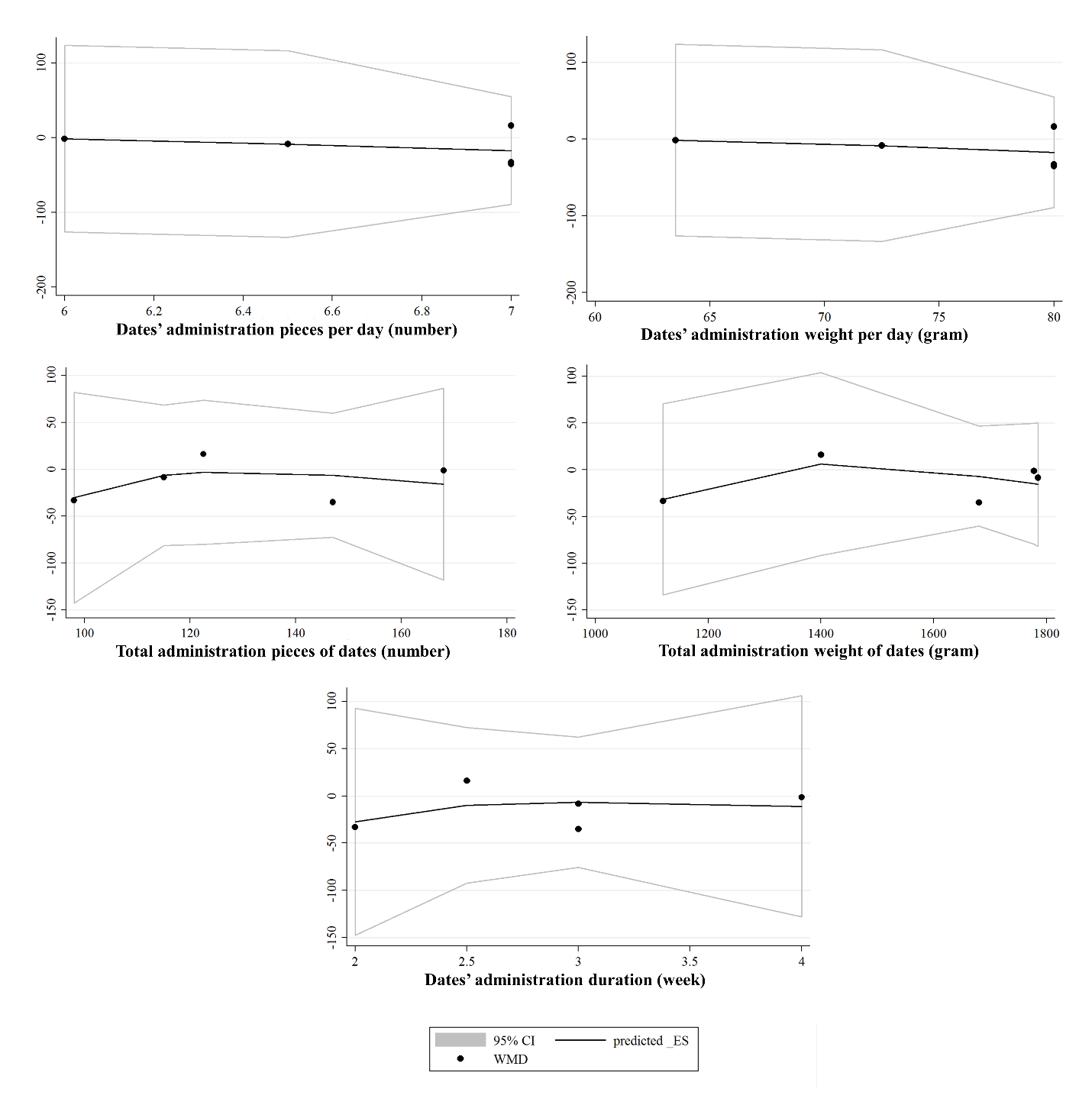
Supplementary Fig. 10:** Dose-response analysis for the association between the administration dosage and duration of dates and changes in the length of the second labor stage (minute, intervention time: late pregnancy)

**
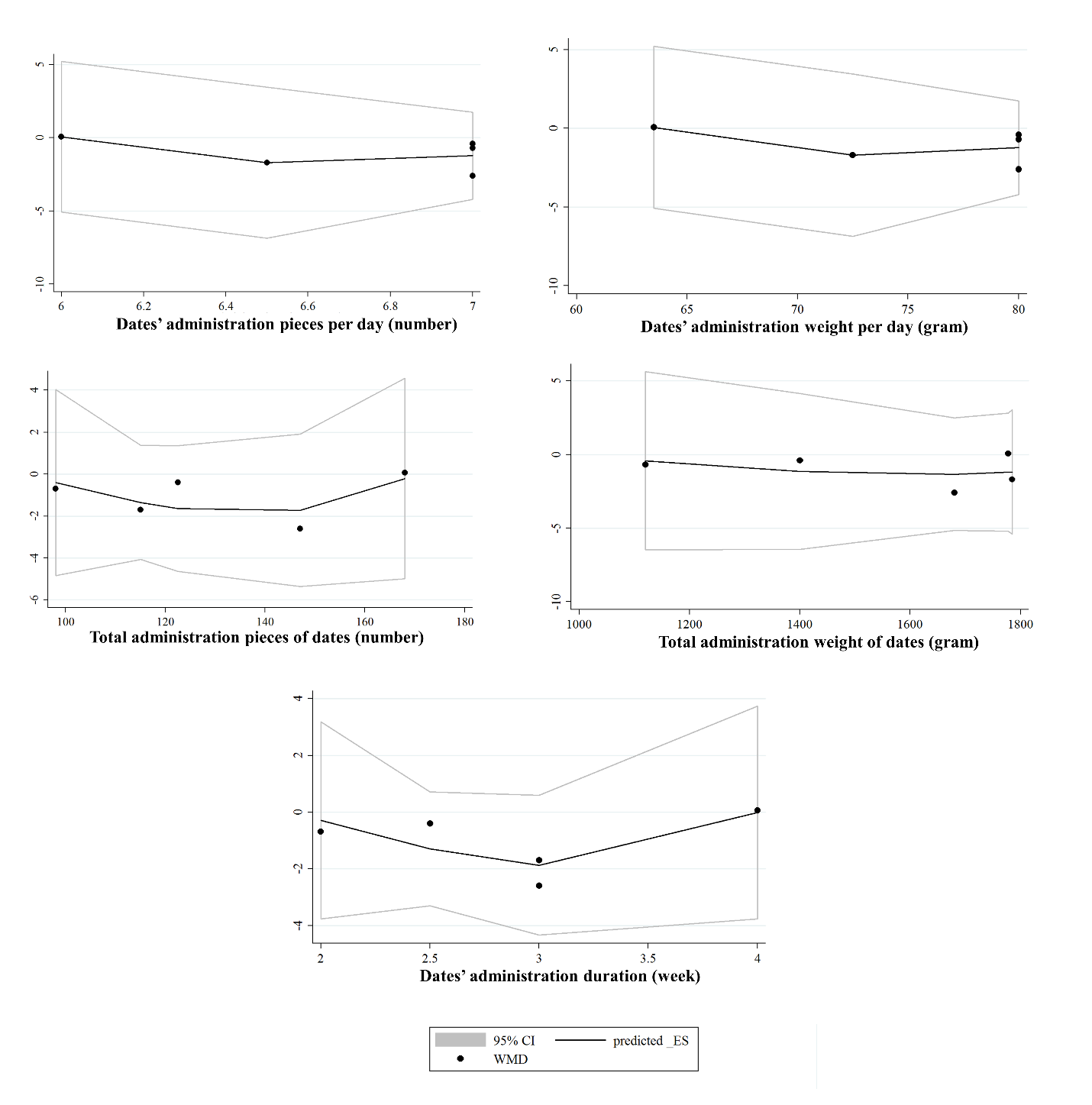
Supplementary Fig. 11:** Dose-response analysis for the association between the administration dosage and duration of dates and changes in the length of the third labor stage (minute, intervention time: late pregnancy)

**
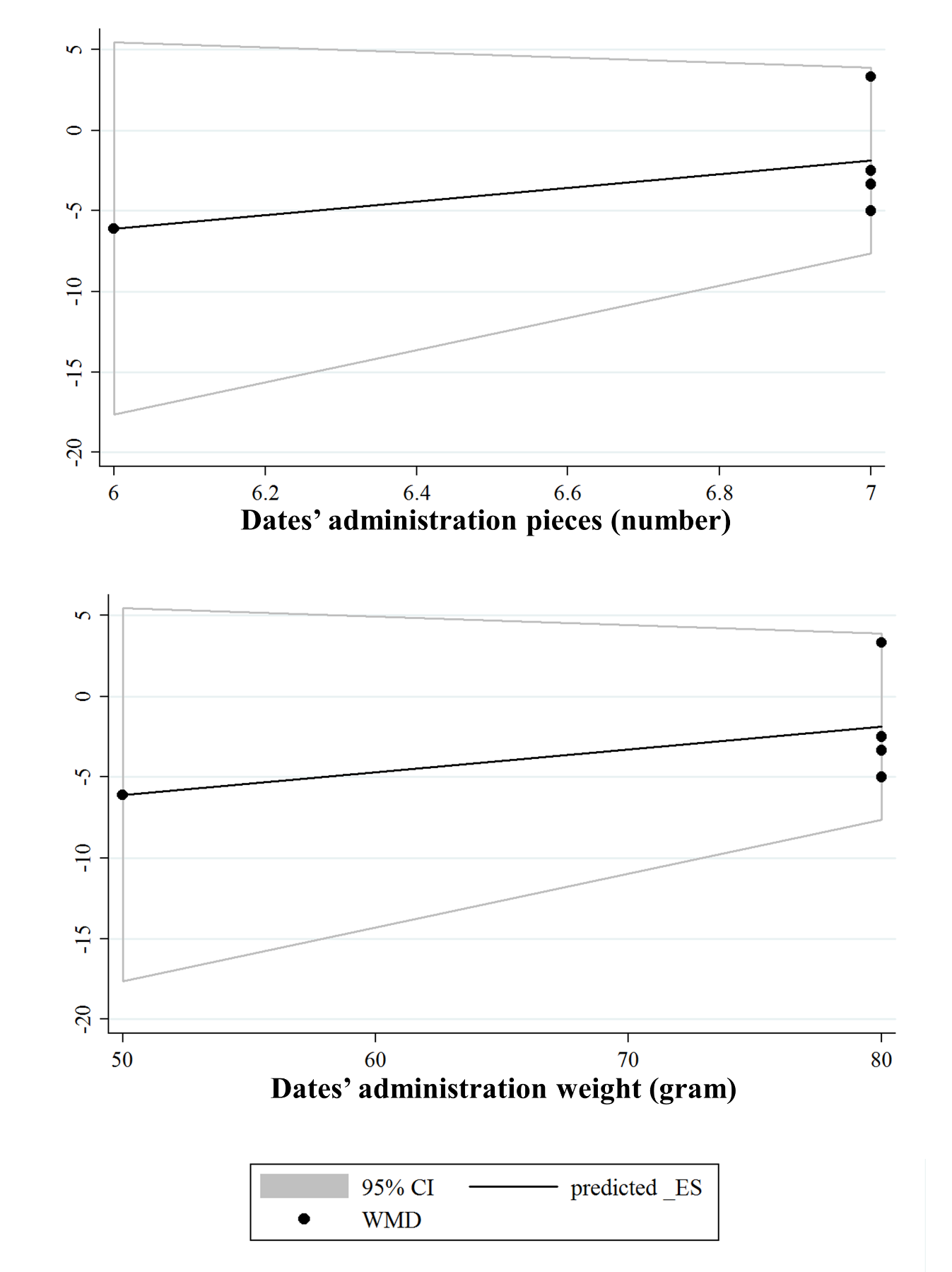
Supplementary Fig. 12:** Dose-response analysis for the association between the administration dosage of dates and changes in the length of the third labor stage (minute, intervention time: labor)

**
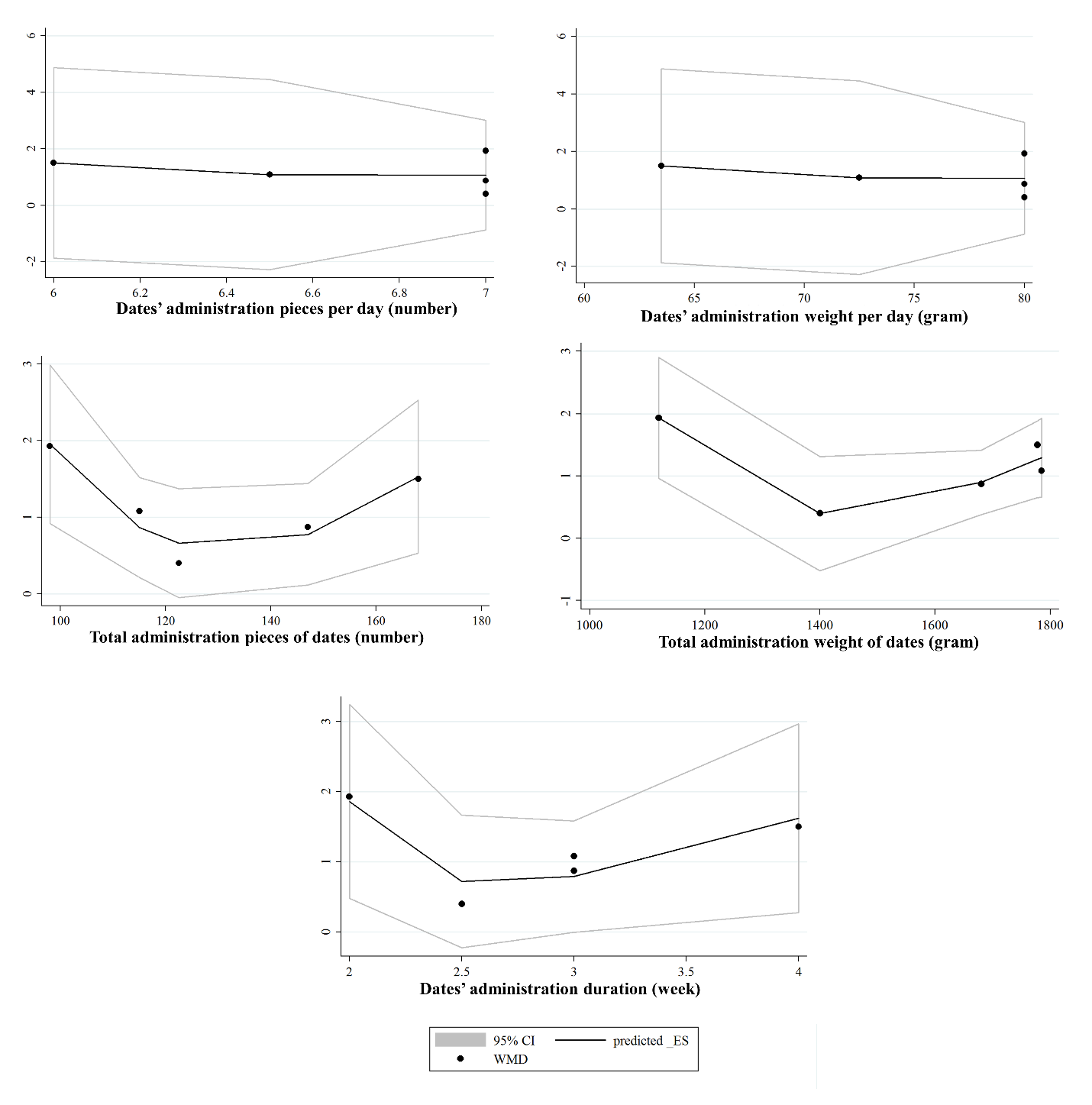
 Supplementary Fig. 13:** Dose-response analysis for the association between the administration dosage and duration of dates and changes in the cervical dilation upon admission (centimeters, intervention time: late pregnancy)

**
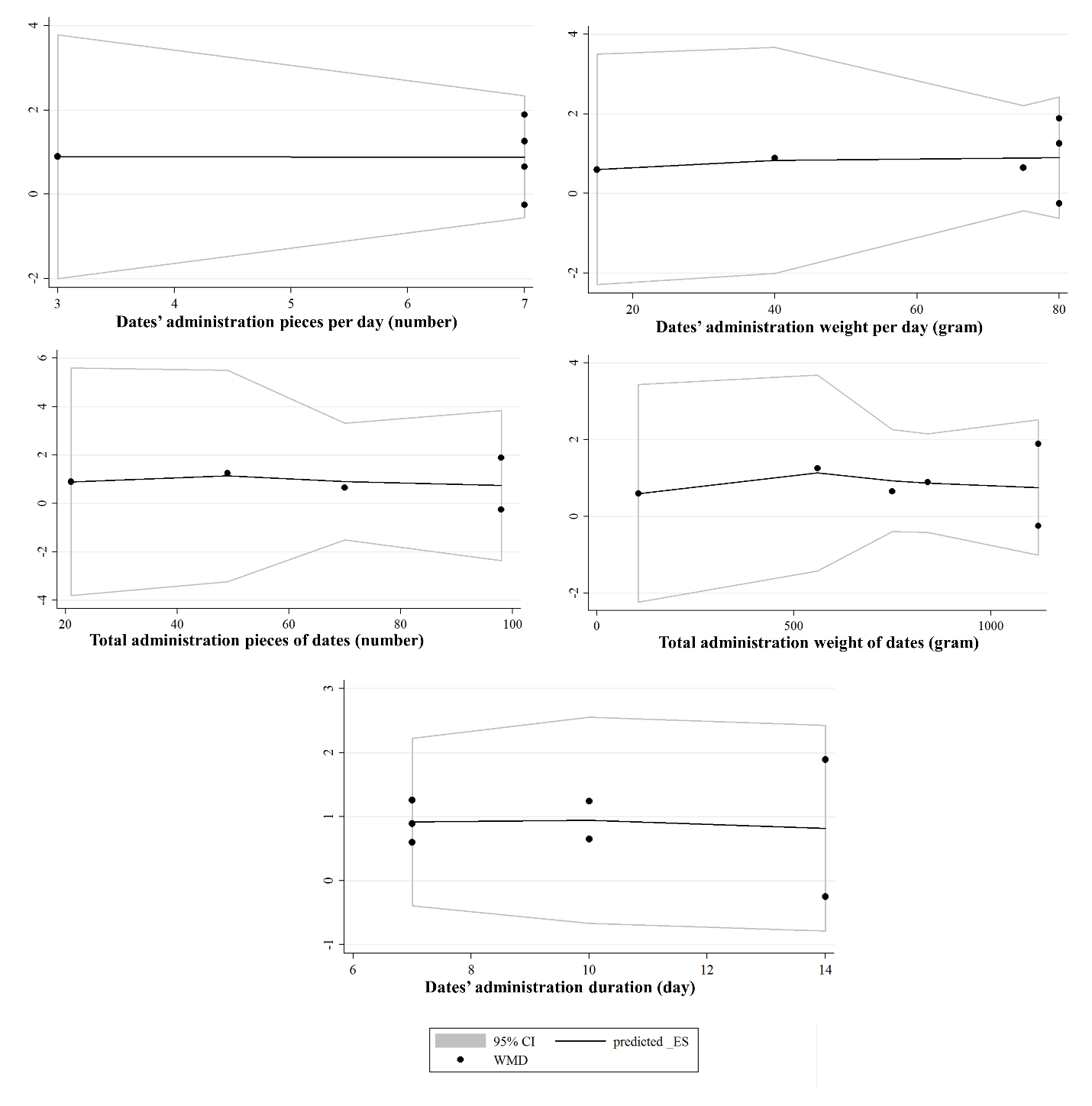
Supplementary Fig. 14:** Dose-response analysis for the association between the administration dosage and duration of dates and changes in the maternal hemoglobin levels (gr/dl, intervention time: the third trimester of pregnancy)

**
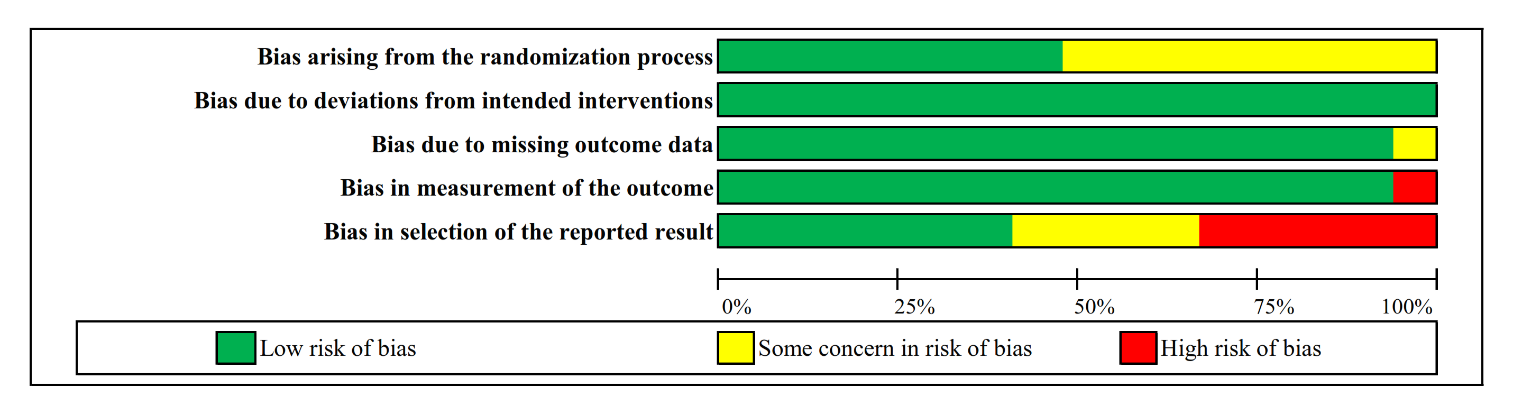
 Supplementary Fig. 15:** Summary of the authors’ judgments about the risk of bias domains across the 15 included randomized controlled trials (RCTs)

**
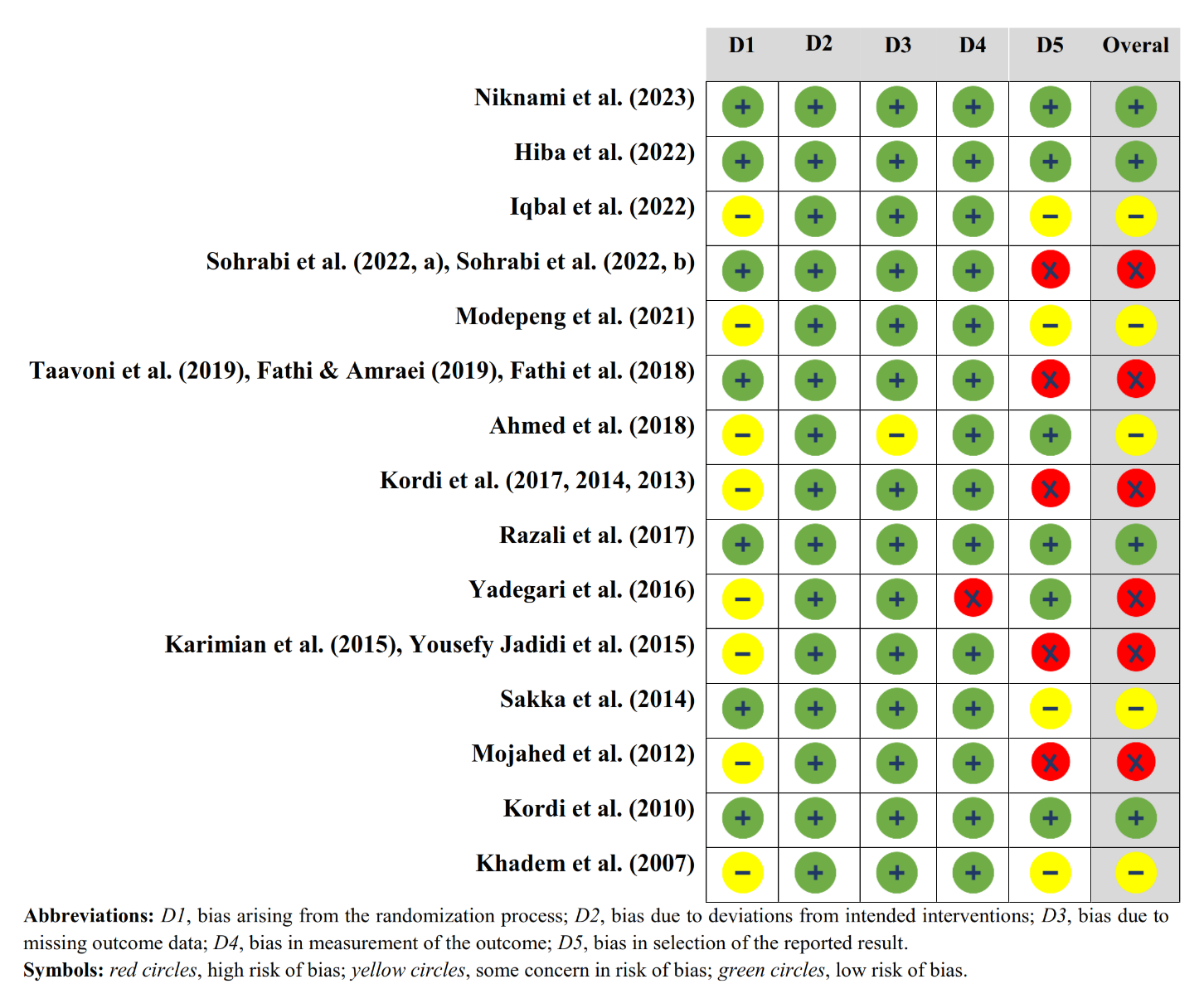
Supplementary Fig. 16:** Summary of the authors’ judgments about the risk of bias domains within the 15 included randomized controlled trials (RCTs)

**
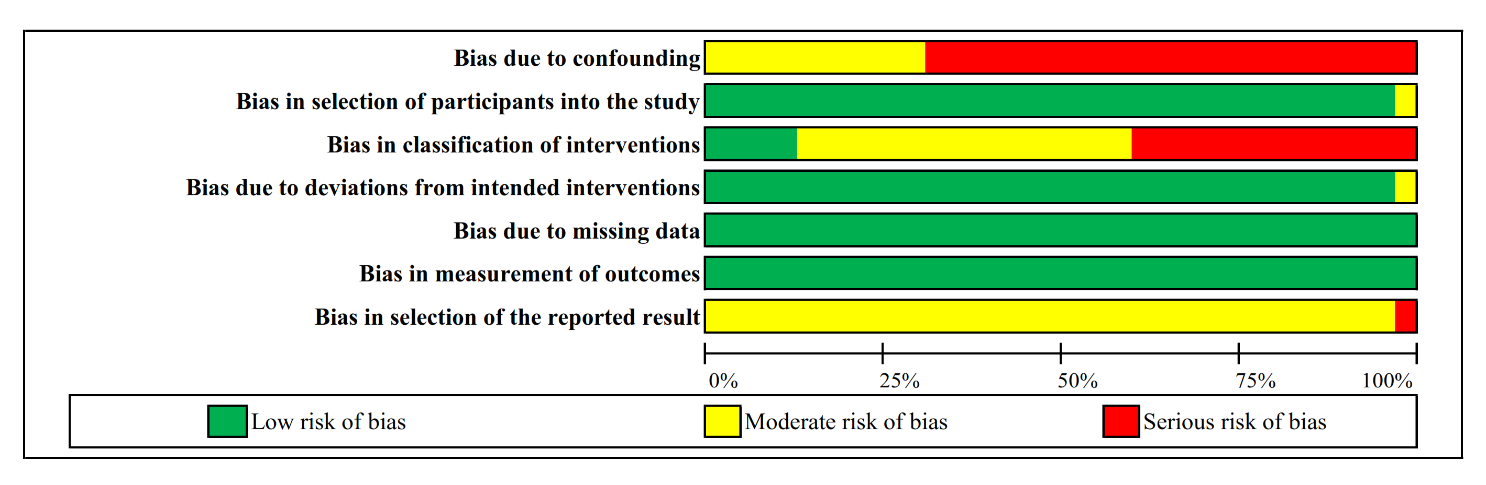
 Supplementary Fig. 17:** Summary of the authors’ judgments about the risk of bias domains across the 33 included non-randomized controlled trials (non-RCTs)

**
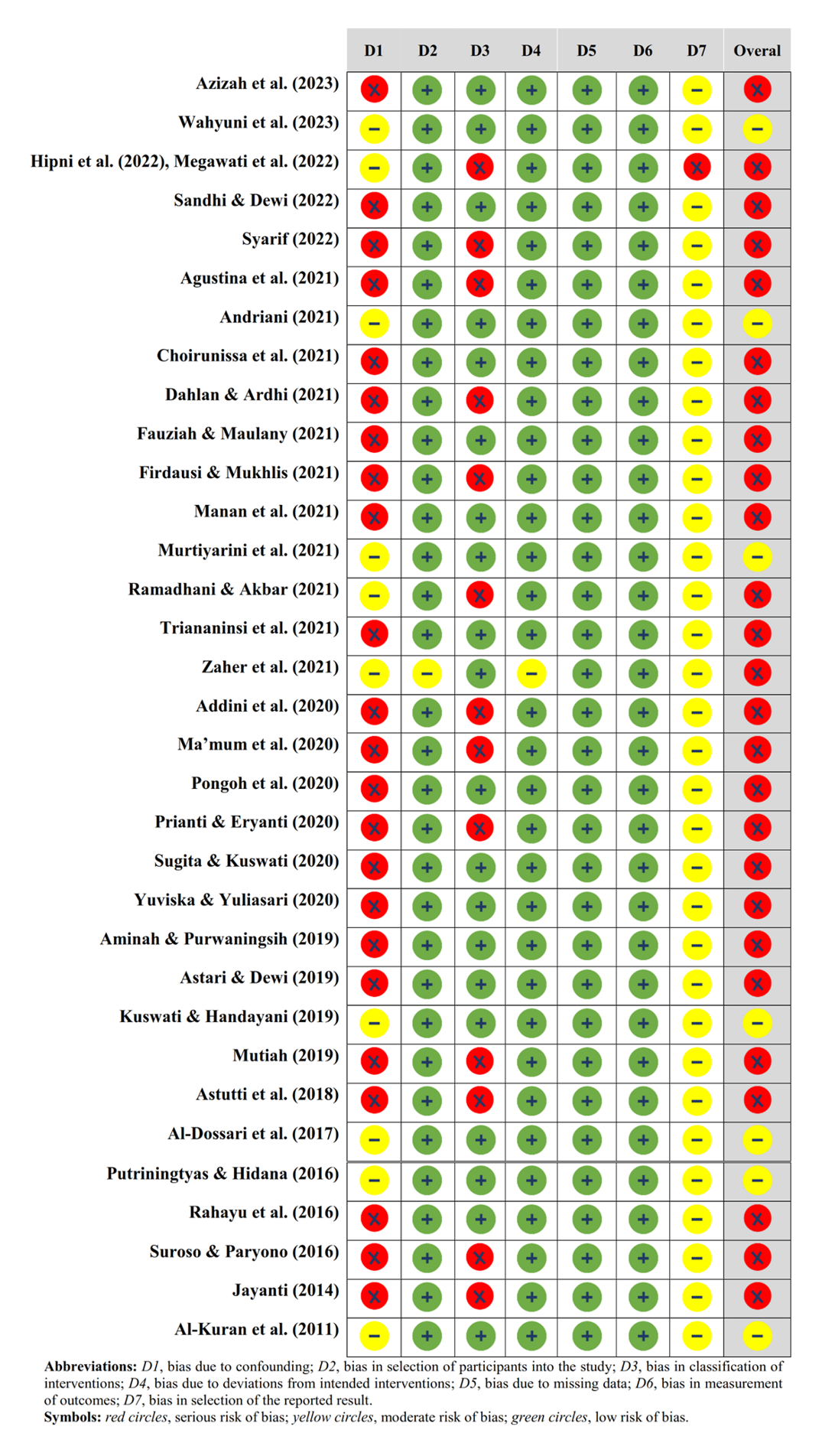
**

**Supplementary Fig. 18:** Summary of the authors’ judgments about the risk of bias domains within the 33 included non-randomized controlled trials (non-RCTs)

**Supplementary Table 1.** PRISMA 2020 checklist for reporting systematic reviews and meta-analyses

| **Section and Topic** | **Item #** | **Checklist item** | **Location where item is reported** |
| --- | --- | --- | --- |
| **TITLE** | | |  |
| Title | 1 | Identify the report as a systematic review. | p 1 (line 3) |
| **ABSTRACT** | | |  |
| Abstract | 2 | See the PRISMA 2020 for Abstracts checklist. | p 3-4 |
| **INTRODUCTION** | | |  |
| Rationale | 3 | Describe the rationale for the review in the context of existing knowledge. | p 6-7 |
| Objectives | 4 | Provide an explicit statement of the objective(s) or question(s) the review addresses. | p 7 |
| **METHODS** | | |  |
| Eligibility criteria | 5 | Specify the inclusion and exclusion criteria for the review and how studies were grouped for the syntheses. | p 8, table 1 |
| Information sources | 6 | Specify all databases, registers, websites, organisations, reference lists and other sources searched or consulted to identify studies. Specify the date when each source was last searched or consulted. | p 8 |
| Search strategy | 7 | Present the full search strategies for all databases, registers and websites, including any filters and limits used. | p 9, Supplementary Table 2 |
| Selection process | 8 | Specify the methods used to decide whether a study met the inclusion criteria of the review, including how many reviewers screened each record and each report retrieved, whether they worked independently, and if applicable, details of automation tools used in the process. | p 9-10 |
| Data collection process | 9 | Specify the methods used to collect data from reports, including how many reviewers collected data from each report, whether they worked independently, any processes for obtaining or confirming data from study investigators, and if applicable, details of automation tools used in the process. | p 9-10 |
| Data items | 10a | List and define all outcomes for which data were sought. Specify whether all results that were compatible with each outcome domain in each study were sought (e.g. for all measures, time points, analyses), and if not, the methods used to decide which results to collect. | Table 1 |
|  | 10b | List and define all other variables for which data were sought (e.g. participant and intervention characteristics, funding sources). Describe any assumptions made about any missing or unclear information. | Table 1, p 9 |
| Study risk of bias assessment | 11 | Specify the methods used to assess risk of bias in the included studies, including details of the tool(s) used, how many reviewers assessed each study and whether they worked independently, and if applicable, details of automation tools used in the process. | p 10 |
| Effect measures | 12 | Specify for each outcome the effect measure(s) (e.g. risk ratio, mean difference) used in the synthesis or presentation of results. | p 10 |
| Synthesis methods | 13a | Describe the processes used to decide which studies were eligible for each synthesis (e.g. tabulating the study intervention characteristics and comparing against the planned groups for each synthesis (item #5)). | p 10 |
|  | 13b | Describe any methods required to prepare the data for presentation or synthesis, such as handling of missing summary statistics, or data conversions. | p 9-10 |
|  | 13c | Describe any methods used to tabulate or visually display results of individual studies and syntheses. | p 10 |
|  | 13d | Describe any methods used to synthesize results and provide a rationale for the choice(s). If meta-analysis was performed, describe the model(s), method(s) to identify the presence and extent of statistical heterogeneity, and software package(s) used. | p 10 |
|  | 13e | Describe any methods used to explore possible causes of heterogeneity among study results (e.g. subgroup analysis, meta-regression). | p 10 |
|  | 13f | Describe any sensitivity analyses conducted to assess robustness of the synthesized results. | p 10 |
| Reporting bias assessment | 14 | Describe any methods used to assess risk of bias due to missing results in a synthesis (arising from reporting biases). | p 10 |
| Certainty assessment | 15 | Describe any methods used to assess certainty (or confidence) in the body of evidence for an outcome. | p 10 |
| **RESULTS** | | |  |
| Study selection | 16a | Describe the results of the search and selection process, from the number of records identified in the search to the number of studies included in the review, ideally using a flow diagram. | p 11, Supplementary Figure 1 |
|  | 16b | Cite studies that might appear to meet the inclusion criteria, but which were excluded, and explain why they were excluded. | p 11, Supplementary Table 3 |
| Study characteristics | 17 | Cite each included study and present its characteristics. | p 11-12 (Table 2) |
| Risk of bias in studies | 18 | Present assessments of risk of bias for each included study. | Supplementary Figures 16, 18 |
| Results of individual studies | 19 | For all outcomes, present, for each study: (a) summary statistics for each group (where appropriate) and (b) an effect estimate and its precision (e.g. confidence/credible interval), ideally using structured tables or plots. | Table 2 |
| Results of syntheses | 20a | For each synthesis, briefly summarise the characteristics and risk of bias among contributing studies. | Supplementary Figures 15, 17 |
|  | 20b | Present results of all statistical syntheses conducted. If meta-analysis was done, present for each the summary estimate and its precision (e.g. confidence/credible interval) and measures of statistical heterogeneity. If comparing groups, describe the direction of the effect. | P 12-20, Figures 1-6 |
|  | 20c | Present results of all investigations of possible causes of heterogeneity among study results. | p 20-21 |
|  | 20d | Present results of all sensitivity analyses conducted to assess the robustness of the synthesized results. | Suppl Figures 2-7 |
| Reporting biases | 21 | Present assessments of risk of bias due to missing results (arising from reporting biases) for each synthesis assessed. | p 22 |
| Certainty of evidence | 22 | Present assessments of certainty (or confidence) in the body of evidence for each outcome assessed. | p 22 |
| **DISCUSSION** | | |  |
| Discussion | 23a | Provide a general interpretation of the results in the context of other evidence. | p 23-27 |
|  | 23b | Discuss any limitations of the evidence included in the review. | p 32 |
|  | 23c | Discuss any limitations of the review processes used. | p 32 |
|  | 23d | Discuss implications of the results for practice, policy, and future research. | p 27-31 |
| **OTHER INFORMATION** | | |  |
| Registration and protocol | 24a | Provide registration information for the review, including register name and registration number, or state that the review was not registered. | p 7 |
|  | 24b | Indicate where the review protocol can be accessed, or state that a protocol was not prepared. | p 7 |
|  | 24c | Describe and explain any amendments to information provided at registration or in the protocol. | N/A |
| Support | 25 | Describe sources of financial or non-financial support for the review, and the role of the funders or sponsors in the review. | p 34 |
| Competing interests | 26 | Declare any competing interests of review authors. | p 33 |

**Supplementary Table 2.** Search characteristics in selected data-sources for the effects of oral consumption of dates in the peripartum period on childbirth and perinatal outcomes

| **Data sources** | **Search strategy** | **Search results** |
| --- | --- | --- |
| WOS (Core Collection) | (AB=(Phoeniceae) OR AB=("Phoenix dactylifera") OR AB=(Nakhl) OR AB=("Date palm") OR AB=("Palm date") OR AB=("Date fruit*") OR TI=(Dates) OR AB=(Khorma) OR AB=(Kurma) OR AB=(Rutab) OR AB=(Ruthab)) AND (AB=(Labor) OR AB=(Labour) OR AB=(Obstetric) OR AB=(Deliver*) OR AB=(Parturition) OR AB=(Parturient*) OR AB=(Childbirth) OR AB=(Birth) OR AB=(Pregnan*) OR AB=(Conception) OR AB=(Gestation) OR AB=(Postpartum) OR AB=(Post-partum) OR AB=(Infant*) OR AB=(Neonate*)) AND (AB=(Intervention*) OR TS=("Clinical trial") OR AB=(Trial) OR AB=(Experiment*) OR AB=(Quasi-experiment*) OR AB=(Quasiexperiment*) OR AB=(Random*) OR AB=(Assign*)) | 323 |
| Scopus | (TITLE-ABS-KEY (Phoeniceae) OR TITLE-ABS-KEY ("Phoenix dactylifera") OR TITLE-ABS-KEY (Nakhl) OR TITLE-ABS-KEY ("Date palm") OR TITLE-ABS-KEY ("Palm date") OR TITLE-ABS-KEY ("Date fruit*") OR TITLE (Dates) OR TITLE-ABS-KEY (Khorma) OR TITLE-ABS-KEY (Kurma) OR TITLE-ABS-KEY (Rutab) OR TITLE-ABS-KEY (Ruthab)) AND (TITLE-ABS-KEY (Labor) OR TITLE-ABS-KEY (Labour) OR TITLE-ABS-KEY (Obstetric) OR TITLE-ABS-KEY (Deliver*) OR TITLE-ABS-KEY (Parturition) OR TITLE-ABS-KEY (Parturient*) OR TITLE-ABS-KEY (Childbirth) OR TITLE-ABS-KEY (Birth) OR TITLE-ABS-KEY (Pregnan*) OR TITLE-ABS-KEY (Conception) OR TITLE-ABS-KEY (Gestation) OR TITLE-ABS-KEY (Post-partum) OR TITLE-ABS-KEY (Postpartum) OR TITLE-ABS-KEY (Infant*) OR TITLE-ABS-KEY (Neonate*)) AND (TITLE-ABS-KEY (Intervention*) OR TITLE-ABS-KEY (Trial) OR TITLE-ABS-KEY (Experiment*) OR TITLE-ABS-KEY (Quasi-experiment*) OR TITLE-ABS-KEY (Quasiexperiment*) OR TITLE-ABS-KEY (Random) OR TITLE-ABS-KEY (Assign*)) | 528 |
| Cochrane Library | (Phoeniceae OR "Phoenix dactylifera" OR Nakhl OR "Date palm" OR "Palm date" OR "Date fruit*" OR Khorma OR Kurma OR Rutab OR Ruthab) in Title Abstract Keyword AND (Labor OR Labour OR Obstetric OR Deliver* OR Parturition OR Parturient* OR Childbirth OR Birth OR Pregnan* OR Conception OR Gestation OR Post-partum OR Postpartum OR Infant* OR Neonate*) in Title Abstract Keyword - (Word variations have been searched) | 41 |
| PubMed | ((Phoeniceae[Mesh] OR Phoeniceae[Tiab] OR "Phoenix dactylifera"[All] OR Phoenix[Tiab] OR Nakhl[Tiab] OR "Date palm"[Tiab] OR "Palm date"[Tiab] OR "Date fruit*"[Tiab] OR "Dates fruit*"[Tiab] OR Dates[Ti] OR Khorma[Tiab] OR Kurma[Tiab] OR Rutab[Tiab] OR Ruthab[Tiab]) AND ("Labor, Obstetric"[Mesh] OR Labor[Tiab] OR Labour[Tiab] OR "Delivery, Obstetric"[Mesh] OR Deliver*[Tiab] OR Parturition[Mesh] OR Parturition[Tiab] OR Parturient*[Tiab] OR Childbirth[Tiab] OR Birth[Tiab] OR Pregnancy[Mesh] OR Pregnan*[Tiab] OR Conception[Tiab] OR Gestation[Tiab] OR Post-partum[Tiab] OR Postpartum[Tiab] OR Infant[Mesh] OR Infant*[Tiab] OR Neonate*[Tiab] OR Newborn*[Tiab])) | 656 |
| Google Scholar | intitle:(Phoeniceae\|"Phoenix dactylifera*"\|"Date palm"\|"Date fruit*"\|Khorma\|Kurma\|Rutab) AND (Labor\|Delivery\|Obstetric\|Parturition\|Pregnan*\|Childbirth\|Gestation\|Post-partum\|Postpartum) AND (Trial\|Intervention*\|Experiment*) | 656 |
| ISC^[[1]](#footnote-1)^ | The search was done in Persian and English:   - Persian: Allfields=خرما و زايمان&Language=.Persian.English.Arabic4050 - English: Allfields= ((Date OR Phoenix dactylifera OR Khorma OR Kurma) AND (Pregnancy OR Labor OR Childbirth))&Language=.Persian.English.Arabic4050 | 204 |
| ICTRP | (Phoeniceae OR Phoenix dactylifera* OR Date palm OR Date fruit* OR Khorma OR Kurma OR Rutab) AND (Labor OR Delivery OR Obstetric OR Parturition OR Pregnan* OR Childbirth OR Gestation OR Post-partum OR Postpartum) | 7 |
| IRCT^[[2]](#footnote-2)^ | The search was done in Persian and English:   - Persian: summary_fa:خرما و زايمان - English: ((@summary:Labor) OR (@summary:Delivery) OR (@summary:Obstetric) OR (@summary:Parturition) OR (@summary:Childbirth) OR (@summary:Gestation) OR (@summary:Post-partum) OR (@summary:Postpartum)) AND ((@summary:Date) OR (@summary:Phoeniceae) OR (@summary:"Phoenix dactylifera") OR (@summary:"Date palm") OR (@summary:"Date fruit*")) | 43 |
| **Abbreviations:** ICTRP, International Clinical Trials Registry Platform; IRCT, Iranian Registry of Clinical Trials; ISC, Scientific Content Database of Islamic World Science Citation Center; WOS, Web of Science. | | |

| **Supplementary Table 3.** Studies excluded after assessing their eligibility by full-text review (n= 33) | |
| --- | --- |
| **Criteria** | **References** |
| Followed a one-group pre-test/post-test design (n= 9) | 1. Dewi RK, Afifi DN, Awatiszahro A, Yulianti D. The effect of dates (Phoenix dactylifera) on breast milk production in post-partum mother day 3-9. J Global Res Public Health. 2021;6(1):52-5. https://doi.org/10.30994/jgrph.v6i1.314. 2. Dewi RK, Puspita NLM, Rohmawati H, Khotimah S, Salsabila G. The effect of dates administration on hemoglobin levels in pregnant women trimester III. J Global Res Public Health. 2021;6(2):124-8. https://doi.org/10.30994/jgrph.v6i2.353. 3. Ginting WM, Br. Girsang DM, Ginting KA, Br. Karo DM, Sirait RA. [The effect of giving dates on increasing haemoglobin levels in pregnant women with anemia (Indonesian)]. Jurnal Penelitian Kebidanan & Kespro. 2021;3(2):45-50. https://doi.org/10.36656/jpk2r.v3i2.651. 4. Irmawati S, Rosdiana R. [Effect of dates extract on increasing Hb levels in pregnant women (Indonesian)]. Jurnal Ilmiah Kesehatan Sandi Husada. 2020;9(2):1063-9. https://doi.org/10.35816/jiskh.v12i2.463. 5. Maulidanita R, Mardiah A. The effect of giving date juice on hemoglobin levels of pregnant women in the third trimester anemia at Pratama Clinic Hanum Medan 2020. Jurnal Kesehatan LLDikti Wilayah 1 (JUKES). 2021;1(1):22-7. https://doi.org/10.54076/jukes.v1i1.129. 6. Rahma Kusuma D, Nara Lintan Mega P, Huda R, Siti K, Gina S. The effect of dates administration on hemoglobin levels in pregnant women trimester III. J Global Res Public Health. 2021;6(2):124-8. https://doi.org/10.30994/jgrph.v6i2.353. 7. Setiowati W. [The influence of palm extract (Phoenix Dactylifera) to increase of hemoglobin level to trimester III pragnant woman (Indonesian)]. J Kesehat STIKES Darul Azhar Batulicin. 2018;6(1):85-95. 8. Widowati R, Kundaryanti R, Lestari PP. [The effect of giving dates extract on increasing hemoglobin levels in pregnant women (Indonesian)]. Jurnal Al-Azhar Indonesia Seri Sains dan Teknologi. 2019;5(2):60-5. https://doi.org/10.36722/sst.v5i2.351. 9. Wulandari W, Siregar Y, Irianti E, Hasibuan Y. [Effectiveness of date palm extract on improving hemoglobin levels of third trimester pregnant women in Sumiariani Clinic and Pratama Sari Clinic in 2019 (Indonesian)]. Medical Journal. 2019;7(2):356-60. |
| Had insufficient data on the intervention method (n= 7) | 1. Gunawan W, Ode Hajrah W. The effect of date palm juice consumption on the active phase of labor among primigravida. SEAJOM: The Southeast Asia Journal of Midwifery. 2020;6(1):16-21. 2. Maryani D, Himalaya D, Ningsih DA. Effect of date syrup (Phoenix Dactylifera) on hemoglobin of postpartum mothers in midwifery independent practices, Bengkulu City. Open Access Maced J Med Sci. 2022;10(G):413-6. https://doi.org/10.3889/oamjms.2022.8672. 3. Prihartini SD. [The effect of dates juice on the smoothness of the 1^st^ stage of labor (Indonesian)]. Jurnal Program studi Kebidanan Fakultas Ilmu Kesehatan. 2014;1(2):14. 4. Rahayu R. [The effectiveness of adding dates extract in fulfilling the nutrition of anemia pregnant women at the Wedi Health Center, Klaten Regency (Indonesian)]. Jurnal Kebidanan dan Kesehatan Tradisional (J Kebidanan Kesehatan Tradisional). 2017;2(2):97-108. https://doi.org/10.37341/jkkt.v2i2.45. 5. Rosyidah NN, Kiftiyah B. [The effectiveness of giving young dates extract on the acceleration of the first stage of labor (Indonesian)]. Jurnal Keperawatan Dan Kebidanan. 2017;9(1):http://jurnalonline.lppmdianhusada.ac.id. 6. Winancy W, Aticeh A, Hajrah WO. The effect of date palm juice consumption on the active phase of labor among primigravida. SEAJOM: The Southeast Asia Journal of Midwifery. 2020;6(1):16-21. 7. Yulinda D, Azizah I. [The effect of date juice on prolactin and expulsion of breast milk in postpartum mothers at Bpm Pipin Heryanti Yogyakarta in 2017 (Indonesian)]. Media Ilmu Kesehat. 2017;6(3):195-8. https://doi.org/10.30989/mik.v6i3.196. |
| Conducted intervention in the first or second trimester of pregnancy (n= 4) | 1. Jannah M, Puspaningtyas M. [Increasing Hb levels of pregnant women with dates palm juice and green bean juice in Pekalongan (Indonesian)]. PLACENTUM: Jurnal Ilmiah Kesehatan Dan Aplikasinya. 2018;6(2):1-6. https://doi.org/10.13057/placentum.v%vi%i.22518. 2. Megasari M, Husanah E. The effectiveness of consumption of dates on increased hemoglobin levels in pregnant women with mild anemia at the Arrabih Primary Clinic. Science Midwifery. 2021;9(2):463-5. 3. Sendra E, Pratamaningtyas S, Panggayuh A. [The effect of consumption of dates (Phoenix Dactylifera) on increased hemoglobin levels in trimester II pregnant women at the Kediri Health Center (Indonesian)]. Jurnal Ilmu Kesehatan. 2016;5(1):96-104. https://doi.org/10.32831/jik.v5i1.119. 4. Sumarni T, Yuliwati N. The effectiveness of dates and beets on hemoglobin levels in trimester II pregnant women at BPM Titin Sumarni. Int J Med Health. 2023;2(1):53-9. https://doi.org/10.55606/ijmh.v2i1.991. |
| Recruited participants aged less than 18 or more than 45 years (n= 2) | 1. Purnama A, Melawati M. The benefits of dates ruthab on blood volume on labor stage 4^th^. J Nurs Pract. 2019;3(1):72-8. https://doi.org/10.30994/jnp.v3i1.67. 2. Saidah H, Sari DK. [Differences in the effectiveness of giving dates and oxytocin massage on breast milk production for breastfeeding mothers 0-6 months (Indonesian)]. Judika (Jurnal Nusantara Medika). 2021;5(2):71-84. https://doi.org/10.29407/judika.v5i2.17439. |
| Administered dates in combination with other fruits or herbal agents (n= 8) | 1. Anggiani N, Farlikhatun L. Comparison of date milk and oxytocin massage on increasing breast milk production in breastfeeding mothers in the Jiput Health Center Working Area, Pandeglang Regency, 2022. Jurnal Eduhealth. 2023;14(01):107-14. 2. Astuti Y, Susiloningtyas I. The effect of mixed juice of young green coconut and date fruits on the duration of active phase of labor. Jurnal Kebidanan dan Kesehatan Tradisional. 2021:7-15. https://doi.org/10.37341/jkkt.v0i0.229. 3. Baska DY, Wahyuni EE, Yulyana N. Intervention of Kusuma milk-shake drink on cervical dilatation and duration of labor: Experience from Bengkulu, Indonesia. Althea Med J. 2023;10(1):21-6. https://doi.org/10.15850/amj.v10n1.2824. 4. Martasari BL, Cahyadi W, Nugraha GI, Husin F, Susiarno H, Hidayat YM, et al. The effect of mixed-fruit juice on uterine contractions and cervical dilatation during the first stage of delivery. Glob Med Health Commun. 2019;7(1):7-14. 5. Mohammadierad R, Mohammad-Alizadeh-Charandabi S, Mirghafourvand M, Fazil F. Effect of saffron with or without date sugar on intensity of pain and anxiety during labor in primiparous females: A randomized, controlled trial. Iran Red Crescent Med J. 2018;20(1):1-8. https://doi.org/10.5812/ircmj.61289. 6. Rahayu S, Wahyuni S. The effect of mixture of citrus dates juice extract (Phoenix Dactylifera L) on blood profile changes for post-partum mother. Indian J Public Health Res Dev. 2019;10(8):1097‐102. 7. Suryati Y, Murtiningsih M, Fitriani H, Mulyani S, Pragholapati A. The effect of spinach combined with date juice on hemoglobin concentration among pregnant women in Indonesia: A queasy experimental design. Mal J Med Health Sci. 2022;18(Supp 17):291-5. 8. Tanasinpiboon C, Pavadhgul P, Bumrungpert A. Consumption of palm date herbal beverage: Effect on maternal and infant nutritional status. Thai J Public Health. 2021;51(3):234-42. |
| Used either dates or other carbohydrate sources based on women demands (n= 3) | 1. Rahmani R, Khakbazan Z, Yavari P, Granmayeh M, Yavari L. Effect of oral carbohydrate intake on labor progress: Randomized controlled trial. Iran J Public Health. 2012;41(11):59-66. 2. Rahmani Bilandi R, Moslem A, Rahmani B M, Askari F. [The effect of food intake during labor on maternal outcomes: A randomized controlled trial (Persian)]. Intern Med Today. 2009;15(2):17-23. 3. Rahmani Bilandi R, Khakbazan Z, Bahri N, Baloochi T, Khargani R. [The effect of food intake during labor on pain and anxiety in pregnant women (Persian)]. Zahedan J Res Med Sci. 2012;13(8):e93776. |

**Supplementary Table 4.** Subgroup analyses for the effects of oral consumption of dates in the peripartum period on childbirth and perinatal outcomes

| **Variables** | | **ESs (n)** | **I^2^** | **Cochran’s Q test** | **WMD/RR^*^**  **(95% CI)** | **P-within** |
| --- | --- | --- | --- | --- | --- | --- |
| **Gestation length (days, intervention time: late pregnancy, I^2^ overal: 9.6%)**^1, 2^ | |  |  |  |  |  |
| Women’s gestational age at recruitment (week) | 36 | 2 | **0.0%** | 0.589 | ˗0.33 (˗2.64, 1.99) | 0.782 |
|  | ≥37 | 2 | **0.0%** | 0.484 | **˗2.54 (˗3.71, ˗1.37)** | **< 0.001** |
| Study’s country of origin | Iran | 2 | **0.0%** | 0.484 | **˗2.54 (˗3.71, ˗1.37)** | **< 0.001** |
|  | Others (i.e., Malaysia, Jordan) | 2 | **0.0%** | 0.859 | ˗0.33 (˗2.64, 1.99) | 0.782 |
| Study’s publication language | English | 2 | **0.0%** | 0.859 | ˗0.33 (˗2.64, 1.99) | 0.782 |
|  | Persian | 2 | **0.0%** | 0.484 | **˗2.54 (˗3.71, ˗1.37)** | **< 0.001** |
| Study’s methodological quality**^**^** | Low | 2 | **0.0%** | 0.484 | ˗2.54 (˗3.71, ˗1.37) | < 0.001 |
|  | Moderate | 1 | - | - | ˗0.70 (˗5.43, 4.03) | 0.772 |
|  | High | 1 | - | - | ˗0.21 (˗2.86, 2.44) | 0.877 |
| Dates’ administration ripening stage | Tamer | 3 | 24.2% | 0.268 | ˗1.74 (˗3.25, ˗0.23) | 0.024 |
|  | Rutab | 1 | - | - | ˗5.04 (˗12.13, 2.05) | 0.164 |
| Dates’ administration variety | Bam Mazafati/Iran | 2 | **0.0%** | 0.484 | **˗2.54 (˗3.71, ˗1.37)** | **< 0.001** |
|  | Not reported | 2 | **0.0%** | 0.859 | ˗0.33 (˗2.64, 1.99) | 0.782 |
| **Duration of labor’s latent phase (minutes, intervention time: late pregnancy, I^2^ overal: 27.5%)**^2,3^ | | | |  |  |  |
| Women’s gestational age at recruitment (week) | 36 | 2 | 47.1% | 0.169 | ˗275.56 (˗466.20, ˗ 84.91) | 0.005 |
|  | >35 | 1 | - | - | ˗190.20 (˗243.03, ˗137.37) | < 0.001 |
| Study’s country of origin | Malaysia | 1 | - | - | ˗197.00 (˗325.60, ˗41.40) | 0.013 |
|  | Jordan | 1 | - | - | ˗396.00 (˗633.13, ˗158.87) | 0.001 |
|  | Pakistan | 1 | - | - | ˗190.20 (˗243.03, ˗137.37) | < 0.001 |
| Study’s methodological quality**^**^** | Moderate | 1 | - | - | ˗396.00 (˗633.13, ˗158.87) | 0.001 |
|  | High | 2 | **0.0%** | 0.935 | ˗190.90 (˗240.93, ˗140.87) | < 0.001 |
| Dates’ administration ripening stage | Tamer | 2 | 47.1% | 0.169 | ˗275.56 (˗466.20, ˗ 84.91) | 0.005 |
|  | Not reported | 1 | - | - | ˗190.20 (˗243.03, ˗137.37) | < 0.001 |
| **Duration of labor’s active phase (minutes, intervention time: late pregnancy, I^2^ overal: 77.0%)**^1,2^ | | | |  |  |  |
| Women’s gestational age at recruitment (week) | 36 | 2 | **0.0%** | 0.611 | ˗35.61 (˗71.93, 0.71) | 0.055 |
|  | ≥37 | 2 | 91.5% | 0.001 | ˗125.14 (˗296.33, 46.06) | 0.152 |
| Study’s country of origin | Iran | 2 | 91.5% | 0.001 | ˗125.14 (˗296.33, 46.06) | 0.152 |
|  | Others (Malaysia, Jordan) | 2 | **0.0%** | 0.611 | ˗35.61 (˗71.93, 0.71) | 0.055 |
| Study’s publication language | English | 3 | 84.2% | 0.002 | ˗86.42 (˗177.66, 4.82) | 0.063 |
|  | Persian | 1 | - | - | ˗43.68 (˗73.59, ˗13.77) | 0.004 |
| Study’s methodological quality**^**^** | Low | 2 | 91.5% | 0.001 | ˗125.14 (˗296.33, 46.06) | 0.152 |
|  | Moderate | 1 | - | - | ˗44.00 (˗92.61, 4.61) | 0.076 |
|  | High | 1 | - | - | ˗0.25 (˗76.65, 29.65) | 0.370 |
| Dates’ administration ripening stage | Tamer | 3 | 84.2% | 0.002 | ˗86.42 (˗177.66, 4.82) | 0.063 |
|  | Rutab | 1 | - | - | ˗43.68 (˗73.59, ˗13.77) | 0.004 |
| Dates’ administration variety | Bam Mazafati/Iran | 2 | 91.5% | 0.001 | ˗125.14 (˗296.33, 46.06) | 0.152 |
|  | Not reported | 2 | **0.0%** | 0.611 | ˗35.61 (˗71.93, 0.71) | 0.055 |
| **Duration of first labor stage (minutes, intervention time: late pregnancy, I^2^ overall: 70.8%)^2,^**^4^ | | | |  |  |  |
| Women’s gestational age at recruitment (week) | 34 | 1 | - | - | ˗49.80 (˗92.34, ˗7.26) | 0.022 |
|  | ≥35 | 5 | 76.6% | 0.002 | ˗56.40 (˗84.54, ˗28.26) | < 0.001 |
| Women’s parity | 0 | 1 | - | - | ˗81.00 (˗144.93, ˗17.07) | 0.013 |
|  | ≥1 | 3 | **0.0%** | 0.836 | **˗60.47 (˗84.13, ˗36.82)** | **< 0.001** |
|  | Not reported | 2 | 94.2% | <0.001 | ˗44.80 (˗92.95, 3.35) | 0.068 |
| Study’s country of origin | Indonesia | 5 | 74.8% | 0.003 | ˗52.36 (˗78.04, ˗26.68) | < 0.001 |
|  | Pakistan | 1 | - | - | ˗81.00 (˗144.93, ˗17.07) | 0.013 |
| Study’s publication language | English | 1 | - | - | ˗81.00 (˗144.93, ˗17.07) | 0.013 |
|  | Indonesian | 5 | 74.8% | 0.003 | ˗52.36 (˗78.04, ˗26.68) | < 0.001 |
| Study’s methodological quality**^**^** | Low | 4 | 79.7% | 0.002 | ˗49.84 (˗79.23, ˗20.44) | 0.001 |
|  | Moderate | 1 | - | - | ˗66.70 (˗110.59, ˗22.81) | 0.003 |
|  | High | 1 | - | - | ˗81.00 (˗144.93, ˗17.07) | 0.013 |
| Dates’ administration form | Pure | 4 | **0.0%** | 0.975 | **˗69.05 (˗85.30, ˗52.79)** | **< 0.001** |
|  | Juice | 2 | 36.2% | 0.210 | ˗28.35 (˗53.51, ˗3.19) | 0.027 |
| Dates’ administration variety | Ajwa | 1 | - | - | ˗69.81 (˗90.64, ˗48.96) | < 0.001 |
|  | Tunisian | 1 | - | - | ˗66.70 (˗110.59, ˗22.81) | 0.003 |
|  | Sukkari | 1 | - | - | ˗64.20 (˗101.58, ˗26.82) | 0.001 |
|  | Not reported | 3 | 54.7% | 0.110 | ˗40.23 (˗72.76, ˗7.71) | 0.015 |
| **Duration of second labor stage (minutes, intervention time: late pregnancy, I^2^ overall: 90.6%)^1,2^** | | | |  |  |  |
| Women’s gestational age at recruitment (week) | >35 | 1 | - | - | ˗35.04 (˗57.76, ˗12.32) | 0.003 |
|  | 36 | 2 | **0.0%** | 0.394 | 1.50 (˗13.41, 16.41) | 0.844 |
|  | ≥37 | 2 | 97.1% | <0.001 | ˗20.70 (˗44.89, 3.50) | 0.094 |
| Study’s country of origin | Iran | 1 | - | - | ˗8.50 (˗13.00, ˗4.00) | < 0.001 |
|  | Indonesia | 1 | - | - | ˗33.19 (˗40.17, ˗26.21) | < 0.001 |
|  | Malaysia | 1 | - | - | 16.00 (˗20.54, 52.54) | 0.391 |
|  | Jordan | 1 | - | - | ˗1.40 (˗17.73, 14.93) | 0.867 |
|  | Pakistan | 1 | - | - | ˗35.04 (˗57.76, ˗12.32) | 0.003 |
| Study’s publication language | English | 4 | 61.0% | 0.053 | ˗9.26 (˗22.66, 4.14) | 0.176 |
|  | Indonesian | 1 | - | - | ˗33.19 (˗40.17, ˗26.21) | < 0.001 |
| Study’s methodological quality**^**^** | Low | 2 | 97.1% | <0.001 | ˗20.70 (˗44.89, 3.50) | 0.094 |
|  | Moderate | 1 | - | - | ˗1.40 (˗17.73, 14.93) | 0.867 |
|  | High | 2 | 81.5% | 0.020 | ˗11.61 (˗61.46, 38.24) | 0.648 |
| Dates’ administration ripening stage | Tamer | 3 | 13.4% | 0.315 | ˗6.58 (˗13.40, 0.24) | 0.059 |
|  | Not reported | 2 | **0.0%** | 0.879 | ˗33.35 (˗40.02, ˗26.68) | < 0.001 |
| Dates’ administration variety | Bam Mazafati/Iran | 1 | - | - | ˗8.50 (˗13.00, ˗4.00) | < 0.001 |
|  | Ajwa | 1 | - | - | ˗33.19 (˗40.17, ˗26.21) | < 0.001 |
|  | Not reported | 3 | 73.9% | 0.022 | ˗8.80 (˗35.92, 18.32) | 0.525 |
| **Duration of third labor stage (minutes, intervention time: late pregnancy, I^2^ overall: 28.8%)^1,2^** | | | |  |  |  |
| Women’s gestational age at recruitment (week) | >35 | 1 | - | - | ˗2.60 (˗4.22, ˗0.98) | 0.002 |
|  | 36 | 2 | **0.0%** | 0.793 | ˗0.12 (˗1.79, 1.55) | 0.891 |
|  | ≥37 | 2 | 1.1% | 0.315 | **˗1.09 (˗2.06, ˗12.00)** | **0.009** |
| Study’s country of origin | Iran | 1 | - | - | ˗1.70 (˗3.23, ˗0.17) | 0.029 |
|  | Indonesia | 1 | - | - | ˗0.69 (˗1.93, 0.55) | 0.276 |
|  | Malaysia | 1 | - | - | ˗0.40 (˗3.09, 2.29) | 0.771 |
|  | Jordan | 1 | - | - | 0.06 (˗2.07, 2.19) | 0.956 |
|  | Pakistan | 1 | - | - | ˗2.60 (˗4.22, ˗0.98) | 0.002 |
| Study’s publication language | English | 4 | 34.0% | 0.208 | ˗1.40 (˗2.57, ˗0.23) | 0.019 |
|  | Indonesian | 1 | - | - | ˗0.69 (˗1.93, 0.55) | 0.276 |
| Study’s methodological quality**^**^** | Low | 2 | 1.1% | 0.315 | **˗1.09 (˗2.06, ˗12.00)** | **0.009** |
|  | Moderate | 1 | - | - | 0.06 (˗2.07, 2.19) | 0.956 |
|  | High | 2 | 47.0% | 0.020 | ˗1.77 (˗3.86, 0.32) | 0.096 |
| Dates’ administration ripening stage | Tamer | 3 | **0.0%** | 0.377 | ˗0.98 (˗2.10, 0.15) | 0.089 |
|  | Not reported | 2 | 70.3% | 0.067 | ˗1.57 (˗3.44, 0.29) | 0.099 |
| Dates’ administration variety | Bam Mazafati/Iran | 1 | - | - | ˗1.70 (˗3.23, ˗0.17) | 0.029 |
|  | Ajwa | 1 | - | - | ˗0.69 (˗1.93, 0.55) | 0.276 |
|  | Not reported | 3 | 55.1% | 0.108 | ˗1.15 (˗2.96, 0.67) | 0.215 |
| **Total duration of labor (hours, intervention time: labor, I^2^ overall: 48.7%)^2,4^** | |  |  |  |  |  |
| Women’s gestational age at recruitment (week) | >37 | 1 | - | - | ˗1.00 (˗3.09, 1.09) | 0.379 |
|  | Not reported | 2 | 73.8% | 0.051 | ˗1.29 (˗2.09, ˗0.48) | 0.002 |
| Women’s parity | 0 | 1 | - | - | ˗1.00 (˗3.09, 1.09) | 0.379 |
|  | ≥1 | 2 | 73.8% | 0.051 | ˗1.29 (˗2.09, ˗0.48) | 0.002 |
| Study’s country of origin | Indonesia | 2 | 73.8% | 0.051 | ˗1.29 (˗2.09, ˗0.48) | 0.002 |
|  | Saudi Arabia | 1 | - | - | ˗1.00 (˗3.09, 1.09) | 0.379 |
| Study’s publication language | English | 1 | - | - | ˗1.00 (˗3.09, 1.09) | 0.379 |
|  | Indonesian | 2 | 73.8% | 0.051 | ˗1.29 (˗2.09, ˗0.48) | 0.002 |
| Study’s methodological quality**^**^** | Low | 2 | 73.8% | 0.051 | ˗1.29 (˗2.09, ˗0.48) | 0.002 |
|  | Moderate | 1 | - | - | ˗1.00 (˗3.09, 1.09) | 0.379 |
| Dates’ administration form | Pure | 2 | **0.0%** | 0.537 | ˗1.64 (˗2.16, ˗1.11) | <0.001 |
|  | Juice | 1 | - | - | ˗0.86 (˗1.48, ˗0.24) | 0.007 |
| Dates’ administration variety | Sukkari | 1 | - | - | ˗1.68 (˗2.22, ˗1.14) | <0.001 |
|  | Not reported | 2 | **0.0%** | 0.900 | ˗0.87 (˗1.47, ˗0.28) | 0.004 |
| **Duration of labor’s active phase (minutes, intervention time: labor, I^2^ overall: 94.2%)^5^** | | |  |  |  |  |
| Women’s gestational age at recruitment (week) | 37-42 | 2 | **0.0%** | 0.370 | ˗119.27 (˗138.56, ˗99.98) | <0.001 |
|  | 38-42 | 1 | - | - | ˗48.85 (˗145.25, ˗31.50) | <0.001 |
| Control condition | Placebo + standard care | 2 | **0.0%** | 0.370 | ˗119.27 (˗138.56, ˗99.98) | <0.001 |
|  | Drinking non-sweet liquids + standard care | 1 | - | - | ˗48.85 (˗145.25, ˗31.50) | <0.001 |
| Study’s publication language | English | 1 | - | - | ˗122.67 (˗143.34, ˗102.00) | <0.001 |
|  | Persian | 2 | 64.4% | 0.094 | ˗65.22 (˗109.45, ˗20.99) | 0.004 |
| Study’s methodological quality**^**^** | Low | 2 | 97.0% | <0.001 | ˗85.35 (˗157.69, ˗13.01) | <0.001 |
|  | High | 1 | - | - | ˗96.33 (˗150.04, ˗42.62) | 0.021 |
| Dates’ administration ripening stage | Tamer | 2 | 97.0% | <0.001 | ˗85.35 (˗157.69, ˗13.01) | <0.001 |
|  | Not reported | 1 | - | - | ˗96.33 (˗150.04, ˗42.62) | 0.021 |
| Dates’ administration variety | Bam Mazafati/Iran | 1 | - | - | ˗122.67 (˗143.34, ˗102.00) | <0.001 |
|  | Not reported | 2 | 64.4% | 0.094 | ˗65.22 (˗109.45, ˗20.99) | 0.004 |
| **Duration of first labor stage (minutes, intervention time: labor, I^2^ overall: 30.2%)^2^** | | |  |  |  |  |
| Women’s gestational age at recruitment (week) | >37 | 2 | **0.0%** | 0.846 | ˗42.34 (˗75.13, ˗9.56) | 0.011 |
|  | Not reported | 2 | **0.0%** | 0.340 | ˗83.15 (˗112.03, ˗54.27) | <0.001 |
| Women’s parity | 0 | 2 | **0.0%** | 0.846 | ˗42.34 (˗75.13, ˗9.56) | 0.011 |
|  | ≥1 | 2 | **0.0%** | 0.340 | **˗83.15 (˗112.03, ˗54.27)** | **<0.001** |
| Study’s country of origin | Saudi Arabia | 2 | **0.0%** | 0.328 | ˗97.93 (˗182.08, ˗13.79) | 0.023 |
|  | Others (Indonesia, Egypt) | 2 | 63.3% | 0.099 | ˗61.41 (˗98.73, ˗24.10) | 0.001 |
| Study’s publication language | English | 3 | 18.2% | 0.295 | ˗56.52 (˗102.73, ˗10.32) | 0.016 |
|  | Indonesian | 1 | - | - | ˗79.55 (˗109.36, ˗49.47) | <0.001 |
| Study’s methodological quality**^**^** | Low | 2 | 63.3% | 0.099 | ˗61.41 (˗98.73, ˗24.10) | 0.001 |
|  | Moderate | 2 | **0.0%** | 0.328 | **˗97.93 (˗182.08, ˗13.79)** | **0.023** |
| Study arms | Two | 3 | 27.2% | 0.253 | ˗61.57 (˗90.02, ˗33.12) | <0.001 |
|  | Three | 1 | - | - | ˗138.03 (˗254.40, ˗21.66) | 0.020 |
| Dates’ administration form | Pure | 2 | 63.3% | 0.099 | ˗61.41 (˗98.73, ˗24.10) | 0.001 |
|  | Pure followed by drinking water | 2 | **0.0%** | 0.328 | **˗97.93 (˗182.08, ˗13.79)** | **0.023** |
| Dates’ administration ripening stage | Rutab | 1 | - | - | ˗138.03 (˗254.40, ˗21.66) | 0.020 |
|  | Not reported | 3 | 27.2% | 0.253 | ˗61.57 (˗90.02, ˗33.12) | <0.001 |
| Dates’ administration variety | Sukkari | 1 | - | - | ˗79.55 (˗109.36, ˗49.47) | <0.001 |
|  | Rutana | 1 | - | - | ˗138.03 (˗254.40, ˗21.66) | 0.020 |
|  | Not reported | 2 | **0.0%** | 0.846 | ˗42.34 (˗75.13, ˗9.56) | 0.011 |
| **Duration of second labor stage (minutes, intervention time: labor, I^2^ overall: 91.3%)** | | |  |  |  |  |
| Women’s gestational age at recruitment (week) | >37 | 4 | 94.6% | <0.001 | ˗21.96 (˗39.63, ˗4.28) | 0.015 |
|  | Not reported | 2 | **33.4%** | 0.221 | ˗15.58 (˗24.02, ˗7.13) | <0.001 |
| Women’s parity | 0 | 4 | 94.6% | <0.001 | **˗21.96 (˗39.63, ˗4.28)** | **0.015** |
|  | ≥1 | 2 | **33.4%** | 0.221 | ˗15.58 (˗24.02, ˗7.13) | <0.001 |
| Control condition | Standard care | 4 | 64.1% | 0.039 | ˗10.61 (˗17.17, ˗4.04) | 0.002 |
|  | Placebo + Standard care | 2 | **0.0%** | 0.978 | **˗37.65 (˗45.09, ˗30.21)** | **<0.001** |
| Study’s country of origin | Iran | 2 | **0.0%** | 0.978 | **˗37.65 (˗45.09, ˗30.21)** | **<0.001** |
|  | Saudi Arabia | 2 | **0.0%** | 0.903 | ˗10.61 (˗17.08, ˗3.25) | 0.004 |
|  | Others (Indonesia, Egypt) | 2 | 87.5% | 0.005 | ˗11.72 (˗25.58, 2.15) | 0.098 |
| Study’s publication language | English | 4 | 93.3% | 0.295 | ˗15.71 (˗30.58, ˗0.84) | 0.038 |
|  | Others (Indonesian, Persian) | 2 | 75.9% | 0.042 | **˗27.47 (˗45.45, ˗9.50)** | **0.003** |
| Study’s methodological quality**^**^** | Low | 3 | 95.3% | <0.001 | **˗20.47 (˗40.26, ˗0.68)** | **0.043** |
|  | Moderate | 2 | **0.0%** | 0.903 | ˗10.16 (˗17.08, ˗3.25) | 0.004 |
|  | High | 1 | - | - | ˗37.84 (˗53.27, ˗22.41) | <0.001 |
| Study arms | Two | 3 | 75.5% | 0.017 | ˗10.83 (˗19.32, ˗2.33) | 0.013 |
|  | Three | 3 | 87.8% | <0.001 | **˗28.50 (˗47.05, ˗9.95)** | **0.003** |
| Dates’ administration form | Pure | 3 | 75.5% | 0.017 | ˗10.83 (˗19.32, ˗2.33) | 0.013 |
|  | Pure followed by drinking water | 1 | - | - | ˗10.67 (˗21.41, 0.07) | 0.052 |
|  | Syrup | 2 | **0.0%** | 0.978 | **˗37.65 (˗45.09, ˗30.21)** | **<0.001** |
| Dates’ administration ripening stage | Tamer | 1 | - | - | ˗37.59 (˗46.08, ˗29.10) | <0.001 |
|  | Rutab | 1 | - | - | ˗10.67 (˗21.41, 0.07) | 0.052 |
|  | Not reported | 3 | 86.1% | <0.001 | ˗16.37 (˗27.70, ˗5.04) | 0.005 |
| Dates’ administration variety | Bam Mazafati/Iran | 1 | - | - | ˗37.59 (˗46.08, ˗29.10) | <0.001 |
|  | Sukkari | 1 | - | - | ˗19.36 (˗28.19, ˗10.53) | <0.001 |
|  | Rutana | 1 | - | - | ˗10.67 (˗21.41, 0.07) | 0.052 |
|  | Not reported | 3 | 87.6% | <0.001 | ˗15.70 (˗30.21, ˗1.19) | 0.034 |
| **Duration of third labor stage (minutes, intervention time: labor, I^2^ overall: 96.3%)** | | |  |  |  |  |
| Women’s gestational age at recruitment (week) | >37 | 3 | 52.5% | 0.122 | ˗3.66 (˗5.15, ˗2.18) | <0.001 |
|  | Not reported | 2 | 96.5% | <0.001 | ˗0.74 (˗8.90, 7.43) | 0.860 |
| Women’s parity | 0 | 3 | 52.5% | 0.122 | **˗3.66 (˗5.15, ˗2.18)** | **<0.001** |
|  | ≥1 | 2 | 96.5% | <0.001 | ˗0.74 (˗8.90, 7.43) | 0.860 |
| Control condition | Standard care | 4 | 97.0% | <0.001 | ˗1.80 (˗5.77, 2.16) | 0.372 |
|  | Placebo + standard care | 1 | - | - | ˗6.09 (˗8.88, ˗3.30) | <0.001 |
| Study’s country of origin | Saudi Arabia | 2 | 95.2% | <0.001 | 0.49 (˗5.22, 6.20) | 0.866 |
|  | Others (Iran, Indonesia, Egypt) | 3 | 56.3% | 0.101 | ˗4.38 (˗6.13, ˗2.62) | <0.001 |
| Study’s publication language | English | 4 | 97.1% | <0.001 | ˗2.06 (˗6.10, 1.97) | 0.316 |
|  | Indonesian | 1 | - | - | ˗5.00 (˗7.79, ˗2.21) | <0.001 |
| Study’s methodological quality**^**^** | Low | 3 | 56.3% | 0.101 | **˗4.38 (˗6.13, ˗2.62)** | **<0.001** |
|  | Moderate | 2 | 95.2% | <0.001 | 0.49 (˗5.22, 6.20) | 0.866 |
| Study arms | Two | 3 | **0.0%** | 0.386 | **˗3.37 (˗3.95, ˗2.79)** | **<0.001** |
|  | Three | 2 | 97.3% | <0.001 | ˗1.29 (˗10.52, 7.94) | 0.784 |
| Dates’ administration form | Pure | 3 | **0.0%** | 0.386 | ˗3.37 (˗3.95, ˗2.79) | <0.001 |
|  | Pure followed by drinking water | 1 | - | - | 3.33 (2.13, 4.53) | <0.001 |
|  | Syrup | 1 | - | - | ˗6.09 (˗8.88, ˗3.30) | <0.001 |
| Dates’ administration ripening stage | Tamer | 1 | - | - | ˗6.09 (˗8.88, ˗3.30) | <0.001 |
|  | Rutab | 1 | - | - | 3.33 (2.13, 4.53) | <0.001 |
|  | Not reported | 3 | **0.0%** | 0.386 | ˗3.37 (˗3.95, ˗2.79) | <0.001 |
| Dates’ administration variety | Bam Mazafati/Iran | 1 | - | - | ˗6.09 (˗8.88, ˗3.30) | <0.001 |
|  | Sukkari | 1 | - | - | ˗5.00 (˗7.79, ˗2.21) | <0.001 |
|  | Rutana | 1 | - | - | 3.33 (2.13, 4.53) | <0.001 |
|  | Not reported | 2 | **0.0%** | 0.463 | ˗3.30 (˗3.89, ˗2.71) | <0.001 |
| **Cervical dilatation two hours post-intervention (centimeters, intervention time: labor, I^2^ overall: 65.6%)^6^** | | | | |  |  |
| Women’s gestational age at recruitment (week) | >37 | 2 | 82.2% | 0.018 | 0.53 (0.03, 1.02) | 0.038 |
|  | Not reported | 1 | - | - | 0.73 (˗0.69, 2.15) | 0.313 |
| Women’s parity | 0 | 2 | 82.2% | 0.018 | 0.53 (0.03, 1.02) | 0.038 |
|  | ≥1 | 1 | - | - | 0.73 (˗0.69, 2.15) | 0.313 |
| Control condition | Standard care | 1 | - | - | 0.73 (˗0.69, 2.15) | 0.313 |
|  | Standard care + Placebo/non-sweet liquids | 2 | 82.2% | 0.018 | 0.53 (0.03, 1.02) | 0.038 |
| Study’s country of origin | Iran | 2 | 82.2% | 0.018 | 0.53 (0.03, 1.02) | 0.038 |
|  | Saudi Arabia | 1 | - | - | 0.73 (˗0.69, 2.15) | 0.313 |
| Study’s publication language | English | 1 | - | - | ˗5.00 (˗7.79, ˗2.21) | <0.001 |
|  | Persian | 2 | 82.2% | 0.018 | 0.53 (0.03, 1.02) | 0.038 |
| Study’s methodological quality**^**^** | Low | 1 | - | - | 0.81 (0.43, 1.19) | <0.001 |
|  | Moderate | 1 | - | - | 0.73 (˗0.69, 2.15) | 0.313 |
|  | High | 1 | - | - | 0.30 (0.12, 0.48) | 0.001 |
| Dates’ administration form | Pure followed by drinking water | 1 | - | - | ˗5.00 (˗7.79, ˗2.21) | <0.001 |
|  | Syrup | 2 | 82.2% | 0.018 | 0.53 (0.03, 1.02) | 0.038 |
| Dates’ administration ripening stage | Tamer | 1 | - | - | 0.81 (0.43, 1.19) | <0.001 |
|  | Rutab | 1 | - | - | 0.73 (˗0.69, 2.15) | 0.313 |
|  | Not reported | 1 | - | - | 0.30 (0.12, 0.48) | 0.001 |
| Dates’ administration variety | Rutana | 1 | - | - | ˗5.00 (˗7.79, ˗2.21) | <0.001 |
|  | Not reported | 2 | 82.2% | 0.018 | 0.53 (0.03, 1.02) | 0.038 |
| **Cervical dilatation upon admission (centimeters, intervention time: late pregnancy, I^2^ overall: 99.3%)^1,2^** | | | | |  |  |
| Women’s gestational age at recruitment (week) | >35 | 1 | - | - | 0.87 (0.60, 1.14) | <0.001 |
|  | 36 | 2 | 88.6% | 0.003 | 0.89 (˗0.18, 1.96) | 0.104 |
|  | 37 or 38 | 2 | 67.9% | 0.077 | **1.64 (0.85, 2.43)** | **<0.001** |
| Study’s country of origin | Iran | 2 | 67.9% | 0.077 | 1.64 (0.85, 2.43) | <0.001 |
|  | Others (Malaysia, Jordan, Pakistan) | 3 | 89.6% | <0.001 | 0.81 (0.32, 1.31) | 0.001 |
| Study’s publication language | English | 3 | 89.6% | <0.001 | 0.81 (0.32, 1.31) | 0.001 |
|  | Persian | 2 | 67.9% | 0.077 | **1.64 (0.85, 2.43)** | **<0.001** |
| Study’s methodological quality**^**^** | Low | 2 | 67.9% | 0.077 | **1.64 (0.85, 2.43)** | **<0.001** |
|  | Moderate | 1 | - | - | 1.50 (0.78, 2.22) | <0.001 |
|  | High | 2 | 90.9% | 0.001 | 0.62 (0.16, 1.08) | 0.008 |
| Dates’ administration ripening stage | Tamer | 3 | 81.3% | 0.005 | 0.92 (0.14, 1.71) | 0.021 |
|  | Rutab | 1 | - | - | 1.93 (1.84, 2.02) | <0.001 |
|  | Not reported | 1 | - | - | 0.87 (0.60, 1.14) | <0.001 |
| Dates’ administration variety | Bam Mazafati/Iran | 2 | 67.9% | 0.077 | 1.64 (0.85, 2.43) | <0.001 |
|  | Not reported | 3 | 89.6% | <0.001 | 0.81 (0.32, 1.31) | 0.001 |
| **Bishop score (intervention time: late pregnancy, I^2^ overall: 0.0%)^2,7^** | | |  |  |  |  |
| Women’s gestational age at recruitment (week) | ≥36 | 1 | - | - | 2.50 (1.72, 3.28) | <0.001 |
|  | 37 or 38 | 2 | **0.0%** | 0.603 | 2.46 (1.87, 3.04) | <0.001 |
| Women’s parity | 0 | 2 | **0.0%** | 0.603 | 2.46 (1.87, 3.04) | <0.001 |
|  | ≥1 | 1 | - | - | 2.50 (1.72, 3.28) | <0.001 |
| Study’s country of origin | Iran | 2 | **0.0%** | 0.603 | 2.46 (1.87, 3.04) | <0.001 |
|  | Indonesia | 1 | - | - | 2.50 (1.72, 3.28) | <0.001 |
| Study’s publication language | English | 1 | - | - | 2.55 (1.86, 3.24) | <0.001 |
|  | Others (Persian, Indonesian) | 2 | **0.0%** | 0.668 | 2.40 (1.76, 3.05) | <0.001 |
| Study’s methodological quality**^**^** | Low | 2 | **0.0%** | 0.603 | 2.46 (1.87, 3.04) | <0.001 |
|  | Moderate | 1 | - | - | 2.50 (1.72, 3.28) | <0.001 |
| Dates’ administration ripening stage | Tamer | 1 | - | - | 2.55 (1.86, 3.24) | <0.001 |
|  | Rutab | 1 | - | - | 2.20 (1.07, 3.33) | <0.001 |
|  | Not reported | 1 | - | - | 2.50 (1.72, 3.28) | <0.001 |
| Dates’ administration variety | Bam Mazafati/Iran | 2 | **0.0%** | 0.603 | 2.46 (1.87, 3.04) | <0.001 |
|  | Tunisian | 1 | - | - | 2.50 (1.72, 3.28) | <0.001 |
| **Frequency of spontaneous onset of labor (intervention time: late pregnancy, I^2^ overall: 76.4%)^1,2^** | | | |  |  |  |
| Women’s gestational age at recruitment (week) | ≥35 | 1 | - | - | 1.37 (1.11, 1.70) | 0.003 |
|  | 36 | 2 | **15.0%** | 0.278 | 1.10 (0.90, 1.22) | 0.081 |
|  | 37-38 | 3 | **30.6%** | 0.237 | **1.51 (1.25, 1.83)** | **<0.001** |
| Study’s country of origin | Iran | 2 | 63.7% | 0.097 | **1.66 (1.11, 2.48)** | **0.013** |
|  | Pakistan | 2 | **0.0%** | 0.845 | 1.39 (1.18, 1.64) | <0.001 |
|  | Others (Malaysia, Jordan,) | 2 | 15.0% | 0.278 | 1.10 (0.99, 1.22) | 0.081 |
| Study’s publication language | English | 4 | 63.9% | 0.040 | 1.21 (1.04, 1.40) | 0.012 |
|  | Persian | 2 | 63.7% | 0.097 | **1.66 (1.11, 2.48)** | **0.013** |
| Study’s methodological quality**^**^** | Low | 2 | 63.7% | 0.097 | **1.66 (1.11, 2.48)** | **0.013** |
|  | Moderate | 2 | 51.0% | 0.153 | 1.25 (1.01, 1.54) | 0.036 |
|  | High | 2 | 81.4% | 0.021 | 1.18 (0.89, 1.57) | 0.244 |
| Dates’ administration ripening stage | Tamer | 3 | 75.3% | 0.017 | 1.19 (0.99, 1.42) | 0.058 |
|  | Rutab | 1 | - | - | 2.14 (1.35, 3.39) | 0.001 |
|  | Not reported | 2 | **0.0%** | 0.845 | 1.39 (1.18, 1.64) | <0.001 |
| Dates’ administration variety | Bam Mazafati/Iran | 2 | 63.7% | 0.097 | 1.66 (1.11, 2.48) | 0.013 |
|  | Not reported | 4 | 63.9% | 0.040 | 1.21 (1.04, 1.40) | 0.012 |
| **Frequency of need for labor induction (intervention time: late pregnancy, I^2^ overall: 0.0%)^2,7^** | | | |  |  |  |
| Women’s gestational age at recruitment (week) | ≥35 | 1 | - | - | 0.41 (0.22, 0.76) | 0.004 |
|  | 36-38 | 5 | **0.0%** | 0.832 | 0.50 (0.39, 0.63) | <0.001 |
| Women’s parity | 0 | 4 | **0.0%** | 0.643 | 0.49 (0.38, 0.63) | <0.001 |
|  | ≥1 | 1 | - | - | 0.50 (0.05, 5.22) | 0.563 |
| Study’s country of origin | Iran | 2 | **0.0%** | 0.613 | 0.49 (0.36, 0.65) | <0.001 |
|  | Pakistan | 2 | **0.0%** | 0.790 | 0.43 (0.28, 0.67) | <0.001 |
|  | Indonesia | 2 | **0.0%** | 0.899 | 0.43 (0.18, 1.04) | 0.062 |
|  | Malaysia | 1 | - | - | 0.75 (0.34, 1.68) | 0.483 |
| Study’s publication language | English | 4 | **0.0%** | 0.690 | 0.49 (0.34, 0.71) | <0.001 |
|  | Persian | 2 | **0.0%** | 0.613 | 0.49 (0.36, 0.65) | <0.001 |
|  | Indonesian | 1 | - | - | 0.42 (0.16, 1.09) | 0.075 |
| Study’s methodological quality**^**^** | Low | 3 | **0.0%** | 0.844 | 0.48 (0.36, 0.63) | 0.030 |
|  | Moderate | 1 | **0.0%** | 0.944 | 0.46 (0.26, 0.83) | 0.010 |
|  | High | 2 | 28.1% | 0.238 | **0.52 (0.29, 0.94)** | **<0.001** |
| Dates’ administration ripening stage | Tamer | 2 | 18.8% | 0.267 | 0.52 (0.33, 0.81) | 0.005 |
|  | Rutab | 1 | - | - | 0.52 (0.35, 0.77) | 0.001 |
|  | Not reported | 4 | **0.0%** | 0.993 | 0.43 (0.29, 0.64) | <0.001 |
| Dates’ administration variety | Bam Mazafati/Iran | 2 | **0.0%** | 0.613 | 0.49 (0.36, 0.65) | <0.001 |
|  | Ajwa | 1 | - | - | 0.42 (0.16, 1.09) | 0.075 |
|  | Not reported | 4 | **0.0%** | 0.690 | 0.49 (0.34, 0.71) | <0.001 |
| **Frequency of spontaneous vaginal delivery (intervention time: late pregnancy, I^2^ overall: 0.0%)^2,7^** | | | |  |  |  |
| Women’s gestational age at recruitment (week) | ≥35 | 1 | - | - | 1.28 (1.02, 1.60) | 0.030 |
|  | 36, 37, or 38 | 3 | **0.0%** | 0.587 | 1.07 (0.98, 1.18) | 0.147 |
| Women’s parity | 0 | 4 | **0.0%** | 0.398 | 1.10 (1.01, 1.20) | 0.029 |
|  | 0-2 | 1 | - | - | 1.04 (0.86, 1.25) | 0.688 |
| Study’s country of origin | Iran | 2 | **0.0%** | 0.514 | 1.09 (0.98, 1.22) | 0.102 |
|  | Others (Pakistan, Malaysia, Indonesia) | 3 | 33.5% | 0.222 | 1.09 (0.94, 1.26) | 0.237 |
| Study’s publication language | English | 3 | 33.5% | 0.222 | 1.09 (0.94, 1.26) | 0.237 |
|  | Persian | 2 | **0.0%** | 0.514 | 1.09 (0.98, 1.22) | 0.102 |
| Study’s methodological quality**^**^** | Low | 2 | **0.0%** | 0.514 | 1.09 (0.98, 1.22) | 0.102 |
|  | Moderate | 1 | - | - | 1.04 (0.86, 1.25) | 0.688 |
|  | High | 2 | 60.5% | 0.111 | 1.13 (0.89, 1.43) | 0.332 |
| Dates’ administration ripening stage | Tamer | 2 | **0.0%** | 0.376 | 1.08 (0.98, 1.19) | 0.128 |
|  | Rutab | 1 | - | - | 1.00 (0.73, 1.37) | <0.001 |
|  | Not reported | 2 | 59.7% | 0.115 | 1.14 (0.91, 1.43) | 0.246 |
| Dates’ administration variety | Bam Mazafati/Iran | 2 | **0.0%** | 0.514 | 1.09 (0.98, 1.22) | 0.102 |
|  | Not reported | 3 | 33.5% | 0.222 | 1.09 (0.94, 1.26) | 0.237 |
| **Frequency of need for instrumental vaginal delivery (intervention time: late pregnancy, I^2^ overall: 75.8%)^1,2^** | | | | |  |  |
| Women’s gestational age at recruitment (week) | ≥35 | 1 | - | - | 0.67 (0.20, 2.26) | 0.515 |
|  | 36, 37, or 38 | 3 | 83.6% | 0.002 | 0.27 (0.03, 2.23) | 0.226 |
| Study’s country of origin | Iran | 2 | 80.3% | 0.024 | 0.14 (0.01, 2.55) | 0.185 |
|  | Others (Pakistan, Malaysia) | 2 | **0.0%** | 0.812 | 0.75 (0.37, 1.53) | 0.432 |
| Study’s publication language | English | 2 | **0.0%** | 0.812 | 0.75 (0.37, 1.53) | 0.432 |
|  | Persian | 2 | 80.3% | 0.024 | 0.14 (0.01, 2.55) | 0.185 |
| Study’s methodological quality**^**^** | Low | 2 | 80.3% | 0.024 | 0.14 (0.01, 2.55) | 0.185 |
|  | High | 2 | **0.0%** | 0.812 | 0.75 (0.37, 1.53) | 0.432 |
| Dates’ administration ripening stage | Tamer | 2 | **0.0%** | 0.625 | 0.72 (0.33, 1.57) | 0.413 |
|  | Rutab | 1 | - | - | 0.04 (0.01, 0.26) | 0.001 |
|  | Not reported | 1 | - | - | 0.67 (0.20, 2.26) | 0.515 |
| Dates’ administration variety | Bam Mazafati/Iran | 2 | 80.3% | 0.024 | 0.14 (0.01, 2.55) | 0.185 |
|  | Not reported | 2 | **0.0%** | 0.812 | 0.75 (0.37, 1.53) | 0.432 |
| **Frequency of need for cesarean section delivery (intervention time: late pregnancy, I^2^ overall: 8.8%)^2,7^** | | | | |  |  |
| Women’s gestational age at recruitment (week) | ≥35 | 1 | - | - | 0.52 (0.27, 1.00) | 0.051 |
|  | 36 | 2 | 62.6% | 0.102 | 0.77 (0.33, 1.78) | 0.540 |
|  | 37 or 38 | 2 | 24.1% | 0.251 | 0.80 (0.49, 1.30) | 0.361 |
| Women’s parity | 0 | 5 | 25.9% | 0.249 | 0.73 (0.52, 1.02) | 0.067 |
|  | 0-2 | 1 | - | - | 1.00 (0.15, 6.64) | <0.001 |
| Study’s country of origin | Iran | 2 | 24.1% | 0.251 | 0.80 (0.49, 1.30) | 0.361 |
|  | Others (Pakistan, Malaysia, Indonesia, Jordan) | 4 | 21.8% | 0.280 | 0.69 (0.44, 1.10) | 0.117 |
| Study’s publication language | English | 4 | 21.8% | 0.280 | 0.69 (0.44, 1.10) | 0.117 |
|  | Persian | 2 | 24.1% | 0.251 | 0.80 (0.49, 1.30) | 0.361 |
| Study’s methodological quality**^**^** | Low | 2 | 24.1% | 0.251 | 0.80 (0.49, 1.30) | 0.361 |
|  | Moderate | 2 | **0.0%** | 0.493 | 0.54 (0.26, 1.11) | 0.096 |
|  | High | 2 | 63.5% | 0.098 | 0.77 (0.36, 1.68) | 0.516 |
| Dates’ administration ripening stage | Tamer | 3 | 38.1% | 0.199 | 0.70 (0.41, 1.21) | 0.204 |
|  | Rutab | 1 | - | - | 0.94 (0.59, 1.51) | 0.811 |
|  | Not reported | 2 | **0.0%** | 0.526 | 0.56 (0.30, 1.04) | 0.065 |
| Dates’ administration variety | Bam Mazafati/Iran | 2 | 24.1% | 0.251 | 0.80 (0.49, 1.30) | 0.361 |
|  | Not reported | 4 | 21.8% | 0.280 | 0.69 (0.44, 1.10) | 0.117 |
| **Breast milk quantity (milliliters, intervention time: postpartum, I^2^ overall: 92.4%)^2^** | | |  |  |  |  |
| Study’s country of origin | Indonesia | 2 | 92.2% | <0.001 | 17.37 (0.52, 34.22) | 0.043 |
|  | Thailand | 1 | - | - | 134.83 (75.94, 193.72) | <0.001 |
| Study’s publication language | English | 1 | - | - | 134.83 (75.94, 193.72) | <0.001 |
|  | Indonesian | 2 | 92.2% | <0.001 | 17.37 (0.52, 34.22) | 0.043 |
| Study’s methodological quality**^**^** | Low | 2 | 92.2% | <0.001 | 17.37 (0.52, 34.22) | 0.043 |
|  | Moderate | 1 | - | - | 134.83 (75.94, 193.72) | <0.001 |
| Dates’ administration form | Pure | 1 | - | - | 134.83 (75.94, 193.72) | <0.001 |
|  | Juice | 1 | - | - | 8.13 (˗1.12, 17.38) | 0.085 |
|  | Not reported | 1 | - | - | 25.37 (23.46, 27.28) | <0.001 |
| Dates’ administration ripening stage | Tamer | 1 | - | - | 134.83 (75.94, 193.72) | <0.001 |
|  | Not reported | 2 | 92.2% | <0.001 | 17.37 (0.52, 34.22) | 0.043 |
| Dates’ administration variety | Deglet Nour/Tunisia | 1 | - | - | 134.83 (75.94, 193.72) | <0.001 |
|  | Not reported | 2 | 92.2% | <0.001 | 17.37 (0.52, 34.22) | 0.043 |
| **Frequency of smoothness of breast milk production (intervention time: postpartum, I^2^ overall: 91.6%)^8^** | | | | |  |  |
| Control condition | Standard care | 2 | 79.1% | 0.029 | 4.72 (0.54, 41.18) | 0.160 |
|  | Standard care + drinking Katuk leaves extract | 1 | - | - | 0.53 (0.32, 0.88) | 0.015 |
| Dates’ administration forms | Pure | 1 | - | - | 0.53 (0.32, 0.88) | 0.015 |
|  | Juice | 2 | 79.1% | 0.029 | 4.72 (0.54, 41.18) | 0.160 |
| **First-day postpartum bleeding rate (milliliters, intervention time: postpartum, I^2^ overall: 85.9%)^2,9^** | | | |  |  |  |
| Women’s gestational age at recruitment (week) | 37-42 | 3 | 76.9% | 0.013 | ˗19.96 (˗39.58, ˗0.34) | 0.046 |
|  | 38-42 | 1 | - | - | ˗58.20 (˗81.02, ˗35.38) | <0.001 |
| Women’s parity | 0 | 1 | - | - | ˗4.42 (˗17.27, 8.43) | 0.500 |
|  | ≥1 | 2 | 90.1% | 0.001 | ˗36.42 (˗76.52, 3.68) | 0.075 |
|  | Not reported | 1 | - | - | ˗58.61 (˗93.69, ˗23.53) | 0.001 |
| Control condition | Standard care | 2 | 55.0% | 0.136 | ˗11.30 (˗23.79, 1.19) | 0.076 |
|  | Intramascular oxytocin | 2 | **0.0%** | 0.985 | **˗58.32 (˗77.45, ˗39.19)** | **<0.001** |
| Study’s publication language | English | 2 | 90.1% | 0.001 | ˗36.42 (˗76.52, 3.68) | 0.075 |
|  | Persian | 2 | 87.6% | 0.004 | ˗28.96 (˗81.82, 23.91) | 0.283 |
| Study’s methodological quality**^**^** | Low | 2 | 87.6% | 0.004 | ˗28.96 (˗81.82, 23.91) | 0.283 |
|  | Moderate | 1 | - | - | ˗58.20 (˗81.02, ˗35.38) | <0.001 |
|  | High | 1 | - | - | ˗17.20 (˗28.02, ˗6.38) | 0.002 |
| Dates’ administration form | Pure | 3 | 87.7% | <0.001 | ˗24.53 (˗48.48, ˗0.58) | 0.045 |
|  | Pure followed by drinking water | 1 | - | - | ˗58.61 (˗93.69, ˗23.53) | 0.001 |
| Dates’ administration ripening stage | Tamer | 2 | 90.1% | 0.001 | ˗36.42 (˗76.52, 3.68) | 0.075 |
|  | Rutab | 2 | 87.6% | 0.004 | ˗28.96 (˗81.82, 23.91) | 0.283 |
| Dates’ administration variety | Bam Mazafati/Iran | 3 | 76.9% | 0.013 | ˗19.96 (˗39.58, ˗0.34) | 0.046 |
|  | Deglet Nour | 1 | - | - | ˗58.20 (˗81.02, ˗35.38) | <0.001 |
| **Maternal hemoglobin levels (gr/dl, intervention time: the third trimester of pregnancy, I^2^ overall: 96.3%)^2,10^** | | | | |  |  |
| Study’s publication language | English | 4 | 94.3% | <0.001 | **1.29 (0.79, 1.78)** | **<0.001** |
|  | Indonesian | 4 | 80.3% | 0.002 | 0.55 (0.27, 0.84) | <0.001 |
| Study’s methodological quality**^**^** | Low | 7 | 96.8% | <0.001 | 0.94 (0.48, 1.39) | <0.001 |
|  | Moderate | 1 | - | - | 0.89 (0.68, 1.10) | <0.001 |
| Dates’ administration forms | Pure | 6 | 95.8% | <0.001 | **0.95 (0.45, 1.44)** | **<0.001** |
|  | Juice | 2 | 74.7% | 0.047 | 0.84 (0.23, 1.45) | 0.007 |
| Dates’ administration variety | Ajwa/Sukari | 2 | 75.5% | 0.043 | 1.06 (0.70, 1.42) | <0.001 |
|  | Tunisian/Egyptian | 2 | 84.3% | 0.012 | 0.86 (0.42, 1.30) | <0.001 |
|  | Not reported | 4 | 98.2% | <0.001 | 0.88 (0.01, 1.74) | 0.046 |
| **Abbreviations:** CI, confidence interval; ESs, effect sizes; I^2^, I-squared statistic; n, number; RR, risk ratio; WMD, weighted mean difference.  ^1^ All women were nulliparous and all used dates in pure form; all studies used a two-arm design.  ^2^ All studies used standard care for the comparison group.  ^3^ All women were nulliparous and all used dates in pure form; all studies were published in English, did not report the dates’ administration variety, and used a two-arm design.  ^4^ None of the studies reported the dates’ administration ripening stage, all used a two-arm design.  ^5^ All women were nulliparous and all used dates in syrup form; all studies were conducted in Iran and had a three-arm design.  ^6^ All studies followed a three-arm design.  ^7^ All studies used dates in pure form and followed a two-arm design.  ^8^ All studies were conducted in Indonesia, were published in Indonesian, followed a two-arm design, and had low methodological quality.  ^9^ All studies were conducted in Iran and followed a two-arm design.  ^10^ All studies were conducted in Indonesia, followed a two-arm design, and used dates in Tamer ripening stage.  **^*^** RR is reported for frequency of spontaneous onset of labor, need for labor induction, spontaneous vaginal delivery, need for instrumental vaginal delivery, need for cesarean section delivery, and smoothness of breast milk production; while WMD is used for other study outcomes.  **^**^** Cochrane’s Risk of Bias (RoB) Assessment Tools: Cochrane’s RoB in Non-randomized Studies-of Interventions (ROBINS-I) and the RoB2 tools were used for non-randomized and randomized studies, respectively. | | | | | | |

**Supplementary Table 5.** Meta-regression for the effects of oral consumption of dates in the peripartum period on childbirth and perinatal outcomes

| **Variables** | **ESs (n)** | ***β* (95% CI)** | **I^2^ residual** | **P-value** |
| --- | --- | --- | --- | --- |
| **Gestation length (days, intervention time: late pregnancy)** | | | | |
| Study’s publication date | 4 | 0.36 (˗1.15, 1.88) | 6.86% | 0.406 |
| Total sample size | 4 | ˗0.01 (˗0.10, 0.07) | 14.05% | 0.550 |
| Dates’ administration duration (week) | 4 | 0.48 (˗7.68, 8.65) | 39.60% | 0.823 |
| Dates’ administration pieces per day | 4 | 0.61 (˗11.68, 12.91) | 30.80% | 0.850 |
| Dates’ administration dosage per day (gram) | 4 | 0.02 (˗0.74, 0.79) | 33.26% | 0.898 |
| Total dates’ administration pieces | 4 | 0.04 (˗0.15, 0.24) | 6.48% | 0.435 |
| Total dates’ administration dosage (gram) | 4 | ˗0.0007 (˗0.01, 0.01) | 25.38% | 0.879 |
| **Duration of labor’s latent phase (minutes, intervention time: late pregnancy)** | | | | |
| Study’s publication date | 3 | 13.53 (˗109.04, 136.12) | 0.0% | 0.394 |
| Total sample size | 3 | 4.68 (˗42.06, 51.43) | 11.32% | 0.424 |
| Dates’ administration duration (week) | 3 | ˗126.87 (˗1357.76, 1104.02) | 3.63% | 0.415 |
| Dates’ administration pieces per day | 3 | 205.09 (˗1366.04, 1776.24) | 0.0% | 0.345 |
| Dates’ administration dosage per day (gram) | 3 | 12.43 (˗82.79, 107.65) | 0.0% | 0.345 |
| Total dates’ administration pieces | 3 | ˗3.34 (˗49.77, 43.08) | 51.38% | 0.528 |
| Total dates’ administration dosage (gram) | 3 | ˗0.29 (˗6.23, 5.65) | 61.39% | 0.643 |
| **Duration of labor’s active phase (minutes, intervention time: late pregnancy)** | | | | |
| Study’s publication date | 4 | ˗11.43 (˗97.51, 74.63) | 84.14% | 0.625 |
| Total sample size | 4 | ˗1.69 (˗6.94, 3.55) | 79.00% | 0.299 |
| Dates’ administration duration (week) | 4 | ˗12.43 (˗313.83, 288.97) | 84.38% | 0.875 |
| Dates’ administration pieces per day | 4 | 38.36 (˗491.73, 568.99) | 84.07% | 0.784 |
| Dates’ administration dosage per day (gram) | 4 | 1.88 (˗30.78, 34.56) | 84.21% | 0.827 |
| Total dates’ administration pieces | 4 | 0.57 (˗7.93, 9.09) | 84.66% | 0.797 |
| Total dates’ administration dosage (gram) | 4 | ˗0.13 (˗0.81, 0.54) | 82.49% | 0.478 |
| **Duration of first labor stage (minutes, intervention time: late pregnancy)** | | | | |
| Study’s publication date | 6 | ˗7.30 (˗19.32, 4.71) | 36.62% | 0.167 |
| Total sample size | 6 | ˗0.32 (˗1.31, 0.66) | 72.83% | 0.416 |
| Dates’ administration duration (week) | 6 | 2.12 (˗27.21, 31.46) | 76.37% | 0.850 |
| Dates’ administration pieces per day | 4 | ˗1.40 (˗20.43, 17.62) | 0.00% | 0.780 |
| Dates’ administration dosage per day (gram) | 4 | ˗0.06 (˗2.66, 2.52) | 0.00% | 0.921 |
| Total dates’ administration pieces | 4 | ˗0.13 (˗1.48, 1.21) | 0.00% | 0.708 |
| Total dates’ administration dosage (gram) | 4 | ˗0.01 (˗0.15, 0.12) | 0.00% | 0.702 |
| **Duration of second labor stage (minutes, intervention time: late pregnancy)** | | | | |
| Study’s publication date | 5 | ˗3.39 (˗7.10, 0.31) | 55.21% | 0.062 |
| Total sample size | 5 | 0.17 (˗0.30, 0.64) | 69.89% | 0.339 |
| Dates’ administration duration (week) | 5 | 12.32 (˗27.57, 52.23) | 73.54% | 0.398 |
| Dates’ administration pieces per day | 5 | ˗27.80 (˗85.95, 30.33) | 67.98% | 0.225 |
| Dates’ administration dosage per day (gram) | 5 | ˗1.60 (˗5.38, 2.17) | 71.97% | 0.269 |
| Total dates’ administration pieces | 5 | 0.20 (˗1.02, 1.42) | 89.75% | 0.635 |
| Total dates’ administration dosage (gram) | 5 | 0.02 (˗0.07, 0.13) | 66.31% | 0.486 |
| **Duration of third labor stage (minutes, intervention time: late pregnancy)** | | | | |
| Study’s publication date | 5 | ˗0.12 (˗0.53, 0.28) | 34.53% | 0.398 |
| Total sample size | 5 | ˗0.007 (˗0.03, 0.01) | 27.31% | 0.437 |
| Dates’ administration duration (week) | 5 | 0.04 (˗2.63, 2.73) | 45.95% | 0.959 |
| Dates’ administration pieces per day | 5 | ˗0.97 (˗5.37, 3.42) | 40.85% | 0.532 |
| Dates’ administration dosage per day (gram) | 5 | ˗0.06 (˗0.33, 0.20) | 39.83% | 0.509 |
| Total dates’ administration pieces | 5 | ˗0.001 (˗0.07, 0.06) | 44.96% | 0.954 |
| Total dates’ administration dosage (gram) | 5 | ˗0.001 (˗0.006, 0.004) | 33.11% | 0.537 |
| **Total duration of labor (hours, intervention time: labor)** | | | | |
| Study’s publication date | 3 | ˗0.32 (˗2.60, 1.95) | 0.00% | 0.319 |
| Total sample size | 3 | 0.04 (˗2.59, 2.68) | 73.76% | 0.857 |
| **Duration of labor’s active phase (minutes, intervention time: labor)** | | | | |
| Study’s publication date | 3 | ˗1.30 (˗82.99, 80.37) | 95.65% | 0.872 |
| Total sample size | 3 | ˗0.75 (˗14.25, 12.75) | 87.45% | 0.608 |
| Dates’ administration duration (minute) | 3 | ˗0.73 (˗8.11, 6.65) | 73.13% | 0.427 |
| Total dates’ administration dosage (milliliter) | 3 | ˗0.01 (˗1.50, 1.46) | 97.02% | 0.900 |
| **Duration of first labor stage (minutes, intervention time: labor)** | | | | |
| Study’s publication date | 4 | 8.20 (˗52.80, 69.20) | 48.70% | 0.621 |
| Total sample size | 4 | 0.59 (˗0.99, 2.18) | 0.00% | 0.251 |
| Dates’ administration pieces | 4 | ˗66.39 (˗118.68, ˗14.10) | 30.24% | **0.027** |
| Dates’ administration dosage (gram) | 4 | ˗66.39 (˗118.68, ˗14.10) | 30.24% | **0.027** |
| **Duration of second labor stage (minutes, intervention time: labor)** | | | | |
| Study’s publication date | 6 | 0.99 (˗3.46, 05.44) | 92.83% | 0.570 |
| Total sample size | 6 | ˗0.12 (˗0.68, 0.43) | 93.00% | 0.563 |
| Dates’ administration pieces | 5 | 27.03 (3.51, 50.54) | 64.10% | **0.035** |
| Dates’ administration dosage (gram) | 6 | ˗0.08 (˗0.85, 0.68) | 92.57% | 0.777 |
| **Duration of third labor stage (minutes, intervention time: labor)** | | | | |
| Study’s publication date | 5 | ˗1.21 (˗3.50, 1.07) | 91.57% | 0.191 |
| Total sample size | 5 | ˗0.02 (˗0.20, 0.15) | 95.97% | 0.674 |
| Dates’ administration pieces | 5 | 4.30 (˗9.19, 17.80) | 96.99% | 0.385 |
| Dates’ administration dosage (gram) | 5 | 0.14 (˗0.30, 0.59) | 96.99% | 0.385 |
| **Cervical dilatation two hours post-intervention (centimeters, intervention time: labor)** | | | | |
| Study’s publication date | 3 | 0.05 (˗0.24, 0.35) | 0.00% | 0.250 |
| Total sample size | 3 | 0.12 (˗0.54, 0.80) | 0.00% | 0.250 |
| Dates’ administration dosage (gram) | 3 | ˗0.006 (˗0.03, 0.02) | 0.00% | 0.251 |
| **Cervical dilatation upon admission (centimeters, intervention time: late pregnancy)** | | | | |
| Study’s publication date | 5 | ˗0.07 (˗0.33, 0.18) | 99.18% | 0.425 |
| Total sample size | 5 | ˗0.01 (˗0.03, 0.01) | 88.25% | 0.297 |
| Dates’ administration duration (week) | 5 | ˗0.06 (˗1.67, 1.54) | 98.86% | 0.901 |
| Dates’ administration pieces per day | 5 | ˗0.38 (˗3.03, 2.27) | 99.51% | 0.680 |
| Dates’ administration dosage per day (gram) | 5 | ˗0.02 (˗0.18, 0.13) | 99.51% | 0.674 |
| Total dates’ administration pieces | 5 | ˗0.004 (˗0.04, 0.03) | 98.55% | 0.737 |
| Total dates’ administration dosage (gram) | 5 | ˗0.0007 (˗0.004, 0.003) | 98.52% | 0.606 |
| **Bishop score (intervention time: late pregnancy)** | | | | |
| Study’s publication date | 3 | 0.002 (˗0.93, 0.94) | 0.00% | 0.980 |
| Total sample size | 3 | 0.0007 (˗0.04, 0.04) | 0.00% | 0.873 |
| Dates’ administration duration (week) | 3 | 0.30 (˗7.86, 8.46) | 0.00% | 0.720 |
| Dates’ administration pieces per day | 3 | ˗0.01 (˗1.75, 1.71) | 0.00% | 0.910 |
| Dates’ administration dosage per day (gram) | 3 | ˗0.002 (˗0.18, 0.17) | 0.00% | 0.892 |
| Total dates’ administration pieces | 3 | 0.0001 (˗0.10, 0.10) | 0.00% | 0.985 |
| Total dates’ administration dosage (gram) | 3 | 0.00008 (˗0.006, 0.006) | 0.00% | 0.892 |
| **Frequency of spontaneous onset of labor (intervention time: late pregnancy)** | | | | |
| Study’s publication date | 6 | 0.001 (˗0.10, 0.10) | 87.13% | 0.961 |
| Total sample size | 6 | ˗0.002 (˗0.01, 0.009) | 87.32% | 0.643 |
| Dates’ administration duration (week) | 6 | ˗0.25 (˗0.86, 0.35) | 87.34% | 0.315 |
| Dates’ administration pieces per day | 6 | 0.16 (˗0.80, 1.13) | 87.20% | 0.660 |
| Dates’ administration dosage per day (gram) | 6 | 0.01 (˗0.05, 0.08) | 87.33% | 0.584 |
| Total dates’ administration pieces | 6 | ˗0.007 (˗0.02, 0.008) | 86.89% | 0.251 |
| Total dates’ administration dosage (gram) | 6 | ˗0.0007 (˗0.002, 0.0009) | 86.99% | 0.290 |
| **Frequency of need for labor induction (intervention time: late pregnancy)** | | | | |
| Study’s publication date | 7 | ˗0.006 (˗0.09, 0.07) | 0.00% | 0.843 |
| Total sample size | 7 | ˗0.0001 (˗0.006, 0.005) | 0.00% | 0.962 |
| Dates’ administration duration (week) | 7 | ˗0.06 (˗0.62, 0.48) | 0.00% | 0.758 |
| Dates’ administration pieces per day | 7 | 0.06 (˗0.70, 0.84) | 0.00% | 0.829 |
| Dates’ administration dosage per day (gram) | 7 | 0.005 (˗0.05, 0.06) | 0.00% | 0.822 |
| Total dates’ administration pieces | 7 | ˗0.001 (˗0.01, 0.01) | 0.00% | 0.830 |
| Total dates’ administration dosage (gram) | 7 | ˗0.0001 (˗0.001, 0.0008) | 0.00% | 0.746 |
| **Frequency of spontaneous vaginal delivery (intervention time: late pregnancy)** | | | | |
| Study’s publication date | 5 | 0.01 (˗0.04, 0.07) | 16.20% | 0.458 |
| Total sample size | 5 | 0.0004 (˗0.002, 0.003) | 23.22% | 0.682 |
| Dates’ administration duration (week) | 5 | 0.15 (˗0.29, 0.60) | 0.00% | 0.349 |
| Dates’ administration pieces per day | 5 | ˗0.04 (˗0.33, 0.24) | 22.00% | 0.653 |
| Dates’ administration dosage per day (gram) | 5 | ˗0.002 (˗0.01, 0.01) | 21.56% | 0.640 |
| Total dates’ administration pieces | 5 | 0.0002 (˗0.005, 0.006) | 28.46% | 0.890 |
| Total dates’ administration dosage (gram) | 5 | 0.00005 (˗0.0005, 0.0006) | 26.47% |  |
| **Frequency of need for instrumental vaginal delivery (intervention time: late pregnancy)** | | | | 0.785 |
| Study’s publication date | 4 | 0.02 (˗0.46, 0.52) | 0.00% | 0.833 |
| Total sample size | 4 | 0.002 (˗0.04, 0.05) | 0.00% | 0.854 |
| Dates’ administration duration (week) | 4 | 0.22 (˗4.02, 4.48) | 0.00% | 0.840 |
| Dates’ administration pieces per day | 4 | 0.34 (˗7.57, 8.26) | 0.00% | 0.870 |
| Dates’ administration dosage per day (gram) | 4 | 0.02 (˗0.50, 0.55) | 0.00% | 0.870 |
| Total dates’ administration pieces | 4 | 0.008 (˗0.08, 0.10) | 0.00% | 0.738 |
| Total dates’ administration dosage (gram) | 4 | 0.0002 (˗0.006, 0.007) | 0.00% | 0.878 |
| **Frequency of need for cesarean section delivery (intervention time: late pregnancy)** | | | | |
| Study’s publication date | 6 | ˗0.001 (˗0.12, 0.11) | 0.00% | 0.969 |
| Total sample size | 6 | ˗0.001 (˗0.01, 0.009) | 0.00% | 0.663 |
| Dates’ administration duration (week) | 6 | ˗0.28 (˗0.89, 0.31) | 0.00% | 0.257 |
| Dates’ administration pieces per day | 6 | 0.38 (˗0.1462, 1.39) | 0.00% | 0.324 |
| Dates’ administration dosage per day (gram) | 6 | 0.02 (˗0.03, 0.08) | 0.00% | 0.365 |
| Total dates’ administration pieces | 6 | ˗0.005 (˗0.02, 0.009) | 0.00% | 0.360 |
| Total dates’ administration dosage (gram) | 6 | ˗0.0006 (˗0.001, 0.0007) | 0.00% | 0.280 |
| **Breast milk quantity (milliliters, intervention time: postpartum)** | | | | |
| Study’s publication date | 3 | 50.81 (˗113.38, 215.00) | 92.37% | 0.315 |
| Total sample size | 3 | 6.90 (˗18.03, 31.85) | 92.19% | 0.176 |
| **Frequency of smoothness of breast milk production (intervention time: postpartum)** | | | | |
| Study’s publication date | 3 | 4.65 (˗9.34, 18.65) | 97.01% | 0.148 |
| Total sample size | 3 | ˗3.75 (˗68.72, 61.20) | 99.25% | 0.597 |
| **First-day postpartum bleeding rate (milliliters, intervention time: postpartum)** | | | | |
| Study’s publication date | 4 | 2.97 (˗5.47, 11.42) | 86.41% | 0.269 |
| Total sample size | 4 | 0.95 (˗3.77, 5.68) | 82.77% | 0.477 |
| Dates’ administration duration (minute) | 3 | 0.43 (˗1.89, 2.75) | 55.03% | 0.256 |
| Dates’ administration pieces | 4 | 7.13 (˗19.18, 33.45) | 76.94% | 0.364 |
| Dates’ administration dosage (gram) | 4 | 0.71 (˗1.91, 3.34) | 76.94% | 0.364 |
| **Maternal hemoglobin levels (gr/dl, intervention time: the third trimester of pregnancy)** | | | | |
| Study’s publication date | 8 | 0.05 (˗1.28, 1.40) | 95.07% | 0.917 |
| Total sample size | 8 | ˗0.004 (˗0.05, 0.04) | 96.64% | 0.813 |
| Dates’ administration duration (day) | 7 | ˗0.004 (˗0.24, 0.23) | 95.00% | 0.962 |
| Dates’ administration pieces per day | 5 | 0.003 (˗0.79, 0.80) | 97.31% | 0.990 |
| Dates’ administration dosage per day (gram) | 6 | 0.004 (˗0.03, 0.03) | 96.25% | 0.750 |
| Total dates’ administration pieces | 5 | ˗0.002 (˗0.04, 0.04) | 96.92% | 0.885 |
| Total dates’ administration dosage (gram) | 6 | 0.0001 (˗0.002, 0.002) | 95.90% | 0.883 |
| **Abbreviation:** CI, confidence interval; ESs, effect sizes; I^2^, I-squared statistic; n, number. | | | | |

**Supplementary Table 6.** Publication bias for the effects of oral consumption of dates in the peripartum period on childbirth and perinatal outcomes

| **Outcomes (intervention time)** | **ESs**  **(number)** | **P-values** | |
| --- | --- | --- | --- |
|  |  | **Begg’s test** | **Egger’s test** |
| Gestation length (late pregnancy) | 4 | 1.000 | 0.751 |
| Duration of labor’s latent phase (late pregnancy) | 3 | 0.296 | 0.426 |
| Duration of labor’s active phase (late pregnancy) | 4 | 0.734 | 0.302 |
| Duration of first labor stage (late pregnancy) | 6 | 0.707 | 0.227 |
| Duration of second labor stage (late pregnancy) | 5 | 0.806 | 0.928 |
| Duration of third labor stage (late pregnancy) | 5 | 1.000 | 0.710 |
| Total duration of labor (labor) | 3 | 1.000 | 0.814 |
| Duration of labor’s active phase (labor) | 3 | 1.000 | 0.669 |
| Duration of first labor stage (labor) | 4 | 0.734 | 0.712 |
| Duration of second labor stage (labor) | 6 | 0.707 | 0.141 |
| Duration of third labor stage (labor) | 5 | 1.000 | 0.935 |
| Cervical dilatation two hours post-intervention (labor) | 3 | 1.000 | 0.557 |
| Cervical dilatation upon admission (late pregnancy) | 5 | 0.806 | 0.841 |
| Bishop score (late pregnancy) | 3 | 0.296 | 0.069 |
| Spontaneous onset of labor (late pregnancy) | 6 | 0.060 | **0.003** |
| Need for labor induction (late pregnancy) | 7 | 1.000 | 0.785 |
| Spontaneous vaginal delivery (late pregnancy) | 5 | 1.000 | 0.840 |
| Need for instrumental vaginal delivery (late pregnancy) | 4 | 0.089 | 0.061 |
| Need for cesarean section delivery (late pregnancy) | 6 | 1.000 | 0.703 |
| Breast milk quantity (postpartum) | 3 | 1.000 | 0.925 |
| Smoothness of breast milk production (postpartum) | 3 | 0.296 | **0.026** |
| First-day postpartum bleeding rate (postpartum) | 4 | 0.734 | 0.185 |
| Maternal hemoglobin levels (the third trimester of pregnancy) | 8 | 0.902 | 0.362 |
| **Abbreviation:** ESs, effect sizes. | | | |

**Supplementary Table 7.** Assessment of the risk of bias of the 15 included randomized controlled trials (RCTs) regarding the effects of oral consumption of dates in the peripartum period on childbirth and perinatal outcomes

| **Authors** | **Domains** | **Judgment** | **Support for judgment** |
| --- | --- | --- | --- |
| **Niknami et al., 2023** | Bias arising from the randomization process | Low | - Quote: “The subjects were selected using the available sampling method and the eligible subjects were divided into the date and control groups using the block randomization method with block sizes of four and six. The Sealed Envelope Simple Randomization Service (2019) was used to generate the randomization list and sequentially numbered; opaque sealed envelopes (SNOSE) were used to hide the allocation of participants.” - Comment: Allocation was adequately concealed, and an adequate method was used to generate the allocation sequence. Also, baseline balances across intervention groups suggest no problem with randomization. |
|  | Bias due to deviations from intended interventions | Low | - Comment: Participants, carers, or personnel were probably not blinded, but co-interventions were assumed to be balanced across intervention groups, and there was no evidence of deviations from the intended interventions that were likely to impact the outcome. |
|  | Bias due to missing outcome data | Low | - Quote: “A total of 202 mothers were screened and 98 mothers were eligible to enter the study. Follow-up data were available for 93 mothers (48 cases in the date group and 45 cases in the control group) to be included in the intention-to-treat (ITT) analysis.” - Comment: There were missing data, but an adequate statistical method was used for handling missing data. |
|  | Bias in measurement of the outcome | Low | - Comment: The outcome assessor was probably not blinded, but the outcome assessment was unlikely to be influenced by knowledge of the intervention received. |
|  | Bias in selection of the reported result | Low | - Comment: All the study’s pre-specified outcomes have been reported in a pre-specified way (registered protocol No. IRCT20210607051505N2). |
| **Hiba et al., 2022** | Bias arising from the randomization process | Low | - Quote: “One hundred forty eligible women were randomized into equal-strength Experimental and Control Groups (70 in each Group) using the lottery technique after obtaining informed written consent”; “The randomization arrangement was performed by means of sealed envelopes numbered from 1 to 10, equally allocated by randomization to the date consumers and the group of control.” - Comment: Allocation was adequately concealed, and an adequate method was used to generate the allocation sequence. Additionally, no information was provided about baseline imbalances across intervention groups to decide on the randomization problem. |
|  | Bias due to deviations from intended interventions | Low | - Comment: No information was provided on the blindness of participants, carers, or personnel, but co-interventions were assumed to be balanced across intervention groups and there was no evidence of deviations from the intended interventions that were likely to impact on the outcome. |
|  | Bias due to missing outcome data | Low | - Comment: No missing outcome data. |
|  | Bias in measurement of the outcome | Low | - Comment: There was no information on the blindness of outcome assessors, but the outcome assessment was unlikely to be influenced by knowledge of the intervention received. |
|  | Bias in selection of the reported result | Low | - Comment: The registered protocol is unavailable, but the review authors believed that reported outcome data were unlikely to have been selected from multiple outcome measurements or multiple analyses of the data. |
| **Iqbal et al., 2022** | Bias arising from the randomization process | Some concern | - Quote: “Random division of the patients in two groups by lottery method was carried out at recruitment.” - Comment: No information was provided about the concealment of allocation, but an acceptable method was used to generate the allocation sequence. Additionally, no information was provided about baseline imbalances across intervention groups to decide on the randomization problem. |
|  | Bias due to deviations from intended interventions | Low | - Comment: No information was provided on the blindness of participants, carers, or personnel, but co-interventions were assumed to be balanced across intervention groups, and there was no evidence of deviations from the intended interventions that were likely to impact the outcome. |
|  | Bias due to missing outcome data | Low | - Comment: No missing outcome data. |
|  | Bias in measurement of the outcome | Low | - Comment: There was no information on the blindness of outcome assessors, but the outcome assessment was unlikely to be influenced by knowledge of the intervention received. |
|  | Bias in selection of the reported result | Some concern | - Comment: The registered protocol is unavailable, and there is insufficient information to exclude the possibility that reported outcome data were selected from multiple outcome measurements or analyses of the data. |
| **Sohrabi et al., 2022, a; Sohrabi et al., 2022, b** | Bias arising from the randomization process | Low | - Quote: “For sampling, at first, participants were selected by the available or easy method and the randomization method was in the form of triple block (ABC) in which each method was placed in sealed envelopes and participants were randomly divided into three groups, including a group receiving saffron–honey syrup, a group receiving date syrup, and a control group”; “… the randomization method was in the form of triple block (ABC) in which each method was placed in sealed envelopes ...” - Allocation was adequately concealed, and an adequate method was used to generate the allocation sequence. Also, baseline balances across intervention groups suggest no problem with randomization. |
|  | Bias due to deviations from intended interventions | Low | - Quotes: “It was not possible to prepare date syrup and honey–saffron syrup with the same shape, color, and taste, so it was not possible to blind the participants completely”; “The syrups were provided in opaque glasses and coded. The codes were delivered by researcher. The assistant researcher Fateme Mohamadi (FM) was unaware of the codes”; “The researcher gave the syrups to the participants and knew the type of intervention but the assistant researcher did not know the type of intervention in the three groups. Thus, the blindness of the study was maintained”; “Each participant was placed in an individual room during her labor. They were not in contact with each other and did not know the type of intervention in each other”; “… also the assistant researcher recorded the results of vaginal examinations.” - Comment: Participants and personnel were unaware of intervention groups, and there was no evidence of deviations from the intended interventions likely to impact the outcome. |
|  | Bias due to missing outcome data | Low | - Quote: “… 63 pregnant women were chosen for participation in each group and the total sample size was 189 people”; “This study was performed on 180 nulliparous women in three 60‑people groups.” - Comment: Three out of 63 women were excluded from each group. Assuming a similar proportion of and similar reasons for missing data in compared groups, the review authors believed the missing data could not clinically influence the observed effect size. |
|  | Bias in measurement of the outcome | Low | - Quotes: “The syrups were provided in opaque glasses and coded. The codes were delivered by researcher. The assistant researcher Fateme Mohamadi (FM) was unaware of the codes”; “The researcher gave the syrups to the participants and knew the type of intervention but the assistant researcher did not know the type of intervention in the three groups”; “… also the assistant researcher recorded the results of vaginal examinations.” - Comment: The outcome assessor was unaware of the intervention received by study participants. |
|  | Bias in selection of the reported result | High | - Comment: Evaluation of pain severity was not documented in the registered protocol (No. IRCT20190924044873N1). Also, two articles were published with, to some extent, similar data. Hence, reported outcome data were likely selected from multiple outcome measurements. |
| **Modepeng et al., 2021** | Bias arising from the randomization process | Some concern | - Quote: “The participants in this study were asked to separate into two groups: an intervention group and a control group”; “The 25 participants in the intervention group were breastfeeding mothers who received health care services at a breastfeeding clinic from one local hospital (district A). The other 25 participants in the control group were breastfeeding mothers who received health care services at a breastfeeding clinic from another hospital (district B).” - Comment: No information was provided about the concealment of allocation, and there was a problem with the sequence generation method. |
|  | Bias due to deviations from intended interventions | Low | - Comment: No information was provided on the blindness of participants, carers, or personnel, but co-interventions were assumed to be balanced across intervention groups, and there was no evidence of deviations from the intended interventions that were likely to impact the outcome. |
|  | Bias due to missing outcome data | Low | - Quote: “At the beginning of the study, there were 25 participants in each group. However, at the end of the study, there were 23 participants in the control group.” - Comment: The review authors believed the missing data could not clinically influence the observed effect size. |
|  | Bias in measurement of the outcome | Low | - Quote: “Participants pumped their breast milk, using an advanced electric breast pump (XILING) for 24 hours, and recorded breastmilk quantity (mL) at week 0, 2, and 4 on the breast milk quantity record form”; “Moreover, the researcher visited the intervention group and the control group in week 2 and 4 to keep a record of the breast milk quantity, dietary, and water intake.” - Comment: The outcome assessor was probably not blinded, but the outcome assessment was unlikely to be influenced by knowledge of the intervention received. |
|  | Bias in selection of the reported result | Some concern | - Comment: The registered protocol is unavailable, and there is insufficient information to exclude the possibility that reported outcome data were selected from multiple outcome measurements or analyses of the data. |

| **Taavoni et al., 2019; Fathi & Amraei, 2019; Fathi et al., 2018** | Bias arising from the randomization process | Low | - Quote: “In this study, samples were randomly assigned to the palm syrup group (card No. 1) or placebo group (card No. 2). The first person who chose a card was randomly selected, and then placed in each of the two groups and the next person was placed in the other group. For example, if the first person chose the number two, she would be placed in the control group, and as a result, the next person would be in the palm syrup group, and again for the third one, both cards were submitted for selection”; “Also, none of the samples were aware of the nature of the numbers 1 and 2 cards, and after the selection of the card, the process of doing research was explained to them.” - Comment: Allocation was adequately concealed, and an adequate method was used to generate the allocation sequence. Also, baseline balances across intervention groups suggest no problem with randomization. |
| --- | --- | --- | --- |
|  | Bias due to deviations from intended interventions | Low | - Quote: “The random allocation of samples was done by the researcher, but in order to prevent bias in the research, the researcher’s colleague (who was not aware of the nature of each person in each of the two groups) was used to record the severity of pain”; “Also, in order to blindness and preventing bias, the research team was used the fellow researchers to record the pain intensity. In fact, the fellow researchers, without any knowledge of the nature of the subjects studied in which of the two groups, recorded the severity of pain…” - Comment: Participants and personnel were unaware of intervention groups, and there was no evidence of deviations from the intended interventions that were likely to impact the outcome. |
|  | Bias due to missing outcome data | Low | - Comment: No missing outcome data. |
|  | Bias in measurement of the outcome | Low | - Quote: “Also, in order to blindness and preventing bias, the research team was used the fellow researchers to record the pain intensity. In fact, the fellow researchers, without any knowledge of the nature of the subjects studied in which of the two groups, recorded the severity of pain…” - Comment: The outcome assessors were unaware of the intervention received by study participants. |
|  | Bias in selection of the reported result | High | - Comment: All the study’s pre-specified outcomes have been documented in a pre-specified way (registered protocol No. IRCT201405182172N16). However, reported outcome data were likely to have been selected from multiple data analyses because different sample sizes and outcomes were reported in three trial reports. |
| **Ahmed et al., 2018** | Bias arising from the randomization process | Some concern | - Quote: “The simple random sampling technique was used to select the participants in the study”; “After study explanation and obtaining consent, the women enrolled in the study were requested to join one of three groups in an open-label manner, with an instruction against cross movement.” - Comment: No information was provided about the concealment of allocation, and there was a problem with the sequence generation method. Additionally, no information was provided about baseline imbalances across intervention groups to decide on the randomization problem. |
|  | Bias due to deviations from intended interventions | Low | - Comment: No information was provided on the blindness of participants, carers, or personnel, but co-interventions were assumed to be balanced across intervention groups, and there was no evidence of deviations from the intended interventions that were likely to impact the outcome. |
|  | Bias due to missing outcome data | Some concern | - Comment: There was unclear information on proportion and reasons for missingness in compared groups, but the review authors believed that the missing data could not clinically influence the observed effect size. |
|  | Bias in measurement of the outcome | Low | - Comment: There was no information on the blindness of outcome assessors, but the outcome assessment was unlikely to be influenced by knowledge of the intervention received. |
|  | Bias in selection of the reported result | Low | - Comment: The registered protocol is unavailable, but the review authors believed that reported outcome data were unlikely to have been selected from multiple outcome measurements or multiple analyses of the data. |
| **Kordi et al., 2017, 2014, 2013** | Bias arising from the randomization process | Some concern | - Quote: “The participants were categorized in one of the two groups of dates’ consumption and lack of dates’ consumption in a manner that days of the week were numbered 1–6 from Saturday to Thursday and 1, 2, 3 numbers were written on one side and 4, 5, 6 were written on the other side of the paper placed in a draw bag. Then, the first paper selected was named intervention and the second one named control group in such a manner that 3 days of the week (Saturday, Sunday, and Monday) were given to the dates’ group and the other 3 days (Tuesday, Wednesday, and Thursday) were given to the control group and this order was maintained till the end of the study.” - Comment: No information was provided about the concealment of allocation, but an acceptable method was used to generate the allocation sequence. Also, baseline balances across intervention groups suggest no problem with randomization. |
|  | Bias due to deviations from intended interventions | Low | - Comment: Participants, carers, or personnel were probably not blinded, but co-interventions were assumed to be balanced across intervention groups, and there was no evidence of deviations from the intended interventions that were likely to impact the outcome. |
|  | Bias due to missing outcome data | Low | - Quote: “A total of 240 women were enrolled in the study. Five cases were excluded given their unwillingness to continue the study, 1 due to sensitivity to date fruit, 11 due to non-use of date fruit for at least two weeks, 9 due to labor induction before 41 weeks of gestation, and 5 due to gestational hypertension. Finally, 210 women remained in the trial.” - Comment: Assuming a similar proportion of missing data in compared groups, the review authors believed the missing data could not clinically influence the observed effect size. |
|  | Bias in measurement of the outcome | Low | - Comment: The outcome assessor was probably not blinded, but the outcome assessment was unlikely to be influenced by knowledge of the intervention received. |
|  | Bias in selection of the reported result | High | - Comment: All the study’s pre-specified outcomes have been documented in a pre-specified way (registered protocol No. IRCT201304072956N4). However, reported outcome data were likely to have been selected from multiple analyses of the data because different sample sizes and outcomes were reported in three reports of the trial. |
| **Razali et al., 2017** | Bias arising from the randomization process | Low | - Quote: “The randomisation sequence was done using sealed envelope numbered 1–10 equally assigned with randomisation for dates-consumers and control group. Hence, they would be assigned to either dates-consumers or control group. Once the selected numbered envelope was taken up, the remaining numbered envelopes would serve as an option for subsequent patients in each group later. The selected numbered envelope was considered void and was not allowed for reselection until replacement. Subsequently, a new batch of sealed and numbered envelopes would replace the old batch of envelopes once it was exhausted.” - Comment: Allocation was adequately concealed, and an adequate method was used to generate the allocation sequence. Also, baseline balances across intervention groups suggest no problem with randomization. |
|  | Bias due to deviations from intended interventions | Low | - Comment: No information was provided on the blindness of participants, carers, or personnel, but co-interventions were assumed to be balanced across intervention groups, and there was no evidence of deviations from the intended interventions that were likely to impact the outcome. |
|  | Bias due to missing outcome data | Low | - Quote: “A total of 179 women were screened for eligibility; 10 women refused to participate. 169 women consented and randomly assigned; 84 women were assigned to the dates-consumers group and 85 women were assigned to the control group. However, only 77 women in each group were analysed as 13 women were lost in the follow-up and two were excluded. - Comment: Seven women were excluded from the experimental group (loss to follow-up: 5, discontinued intervention: 2). Additionally, eight women were excluded from the control group due to loss to follow-up. Assuming to some extent, a similar proportion of missing data in compared groups, the review authors believed that the missing data could not clinically influence the observed effect size. |
|  | Bias in measurement of the outcome | Low | - Comment: There was no information on the blindness of outcome assessors, but all outcomes except the duration of the latent phase of labor were based on the observation/examination. Hence, the outcome assessment was unlikely to be influenced by knowledge of the intervention received. |
|  | Bias in selection of the reported result | Low | - Comment: The registered protocol is unavailable, but the review authors believed that reported outcome data were unlikely to have been selected from multiple outcome measurements or multiple analyses of the data. |
| **Yadegari et al., 2016** | Bias arising from the randomization process | Some concern | - Quote: “The study subjects were randomly placed in one of the two groups of consuming dates and not consuming dates; in a way that at the beginning of sampling, the days of the week from Saturday to Thursday were numbered from one to six. Then, using a table of random numbers, three days of the week were randomly assigned to the date group and another three days to the control group.” - Comment: No information was provided about the concealment of allocation, but an acceptable method was used to generate the allocation sequence. Also, baseline balances across intervention groups suggest no problem with randomization. |
|  | Bias due to deviations from intended interventions | Low | - Comment: No information was provided on the blindness of participants, carers, or personnel, but co-interventions were assumed to be balanced across intervention groups, and there was no evidence of deviations from the intended interventions that were likely to impact the outcome. |
|  | Bias due to missing outcome data | Low | - Quote: “Out of 100 women who participated in the study, three people were excluded from the study due to not completing the PBLAC, three others were excluded due to hospitalization of their newborn, two people were excluded due to consuming less than 50 gr of dates/day, of which five people were in the intervention group, and five people were in the control group.” - Comment: Five out of 50 women were excluded from each group. Assuming a similar proportion of missing data in compared groups, the review authors believed the missing data could not clinically influence the observed effect size. |
|  | Bias in measurement of the outcome | High | - Comment: There was no information on the blindness of outcome assessors. However, the outcome assessment was likely influenced by knowledge of the intervention received as a self-report checklist was used. |
|  | Bias in selection of the reported result | Low | - Comment: All the study’s pre-specified outcomes have been reported in a pre-specified way (registered protocol No. IRCT201503116807N16). |
| **Karimian et al., 2015; Yousefy Jadidi et al., 2015** | Bias arising from the randomization process | Some concern | - Quote: “Samples were randomly classified into two groups by SPSS.” - Comment: No information was provided about the concealment of allocation, but an acceptable method was used to generate the allocation sequence. Also, baseline balances across intervention groups suggest no problem with randomization. |
|  | Bias due to deviations from intended interventions | Low | - Comment: No information was provided on the blindness of participants, carers, or personnel, but co-interventions were assumed to be balanced across intervention groups, and there was no evidence of deviations from the intended interventions that were likely to impact the outcome. |
|  | Bias due to missing outcome data | Low | - Quote: “… 110 women were entered and divided into 55 in each group. In the group of the dates, three women were excluded, and in the control group, one was excluded.” - Comment: Assuming to some extent, a similar proportion of missing data in compared groups, the review authors believed that the missing data could not clinically influence the observed effect size. |
|  | Bias in measurement of the outcome | Low | - Comment: There was no information on the blindness of outcome assessors, but the outcome assessment was unlikely to be influenced by knowledge of the intervention received. |
|  | Bias in selection of the reported result | High | - Comment: All the study’s pre-specified outcomes have been reported in a pre-specified way (registered protocol No. IRCT201207214529N11). However, the reported outcome data were likely to have been selected from multiple outcome measurements (i.e., time points). |
| **Sakka et al., 2014** | Bias arising from the randomization process | Low | - Quote: “For allocation of the participants, we used a random number table and sealed opaque envelopes.” - Comment: Allocation was adequately concealed, and an adequate method was used to generate the allocation sequence. Also, baseline balances across intervention groups suggest no problem with randomization. |
|  | Bias due to deviations from intended interventions | Low | - Comment: No information was provided on the blindness of participants, carers, or personnel, but co-interventions were assumed to be balanced across intervention groups, and there was no evidence of deviations from the intended interventions that were likely to impact the outcome. |
|  | Bias due to missing outcome data | Low | - Comment: No missing outcome data. |
|  | Bias in measurement of the outcome | Low | - Quote: “Mothers were asked to pump both breasts before the first feed in the morning of the third day, using the same brand manual breast pump and measure the pumped milk volumes using infant bottles. These were later collected and measured with a graduated cylinder.” - Comment: The outcome assessor was probably not blinded, but the outcome assessment was unlikely to be influenced by knowledge of the intervention received. |
|  | Bias in selection of the reported result | Some concern | - Comment: The registered protocol is unavailable, and there is insufficient information to exclude the possibility that reported outcome data were selected from multiple outcome measurements or analyses of the data. |
| **Mojahed et al., 2012** | Bias arising from the randomization process | Some concern | - Quote: “Samples were randomly classified into two groups.” - Comment: No information was provided about the concealment of allocation and the sequence generation method. However, baseline balances across intervention groups suggest no problem with randomization. |
|  | Bias due to deviations from intended interventions | Low | - Comment: Participants and personnel were probably unaware of intervention groups, and there was no evidence of deviations from the intended interventions likely to impact the outcome.. |
|  | Bias due to missing outcome data | Low | - Comment: No missing outcome data. |
|  | Bias in measurement of the outcome | Low | - Comment: There was no information on the blindness of outcome assessors, but the outcome assessment was unlikely to be influenced by knowledge of the intervention received. |
|  | Bias in selection of the reported result | High | - Comment: All the study’s pre-specified outcomes have been reported in a pre-specified way (registered protocol No. IRCT201205129708N1). However, the reported outcome data were likely to have been selected from multiple outcome measurements (i.e., time points). |
| **Kordi et al., 2010** | Bias arising from the randomization process | Low | - Comment: Allocation was adequately concealed, and an adequate method was used to generate the allocation sequence using sequentially numbered drug containers of the same color and shape. Also, baseline balances across intervention groups suggest no problem with randomization. |
|  | Bias due to deviations from intended interventions | Low | - Comment: Participants and personnel were blinded, and there was no evidence of deviations from the intended interventions likely to impact the outcome. |
|  | Bias due to missing outcome data | Low | - Comment: No missing outcome data. |
|  | Bias in measurement of the outcome | Low | - Comment: The outcome assessor was blinded. Hence, the outcome assessment was unlikely to be influenced by knowledge of the intervention received. |
|  | Bias in selection of the reported result | Low | - Comment: The registered protocol is unavailable, but the review authors believed that reported outcome data were unlikely to have been selected from multiple outcome measurements or multiple analyses of the data. |
| **Khadem et al., 2007** | Bias arising from the randomization process | Some concern | - Quote: “... samples were initially selected based on the study purpose and then were randomly classified in two groups. Even numbers were allocated to 31 people as the subject group and odd numbers to 31 people as the witness group.” - Comment: No information was provided about the concealment of allocation, and there was a problem with the sequence generation method. However, baseline balances across intervention groups suggest no problem with randomization. |
|  | Bias due to deviations from intended interventions | Low | - Comment: No information was provided on the blindness of participants, carers, or personnel, but co-interventions were assumed to be balanced across intervention groups, and there was no evidence of deviations from the intended interventions that were likely to impact the outcome. |
|  | Bias due to missing outcome data | Low | - Comment: No missing outcome data. |
|  | Bias in measurement of the outcome | Low | - Comment: The outcome assessor was unaware of the intervention received by study participants. |
|  | Bias in selection of the reported result | Some concern | - Comment: The registered protocol is unavailable, and there is insufficient information to exclude the possibility that reported outcome data were selected from multiple outcome measurements or analyses of the data. |

**Supplementary Table 8.** Assessment of the risk of bias of the 33 included non-randomized controlled trials (non-RCTs) regarding the effects of oral consumption of dates in the peripartum period on childbirth and perinatal outcomes

| **Authors** | **Domains** | **Judgment** | **Support for judgment** |
| --- | --- | --- | --- |
| **Azizah et al., 2023** | Bias due to confounding | Serious | - Comment: Some known essential domains (e.g., parity and maternal age) were not recorded or not controlled. Hence, there is potential for confounding the effect of the intervention. |
|  | Bias in selection of participants into the study | Low | - Comment: All participants who would have been eligible for the trial were included in the study, and for each participant start of follow-up and the beginning of intervention coincided. |
|  | Bias in classification of interventions | Low | - Comment: Intervention status is well defined, and intervention definition is based solely on information collected during the intervention. |
|  | Bias due to deviations from intended interventions | Low | - Comment: The critical co-interventions (e.g., routine care) were balanced across experimental and comparator groups. Also, there was no evidence of deviations from the intended interventions likely to impact the outcome. |
|  | Bias due to missing data | Low | - Comment: Data were complete. |
|  | Bias in measurement of outcomes | Low | - Comment: The methods of outcome assessment were comparable across intervention groups. Additionally, the outcome was unlikely to be influenced by knowledge of the intervention received by study participants because it was measured objectively. Moreover, any error in estimating the result seems unrelated to intervention status. |
|  | Bias in selection of the reported result | Moderate | - Comment: A registered protocol is not declared. However, the outcome measurements and analyses are clearly defined and internally and externally consistent. Additionally, there is no indication of the selection of the reported results from multiple analyses. There is also no indication of the cohort selection or subgroups for analysis and reporting based on the results. |
| **Wahyuni et al., 2023** | Bias due to confounding | Moderate | - Comment: Confounding expected but most known important confounding domains (i.e., parity, gravidity, gestational and maternal ages at recruitment) were appropriately measured and controlled. Additionally, the reliability and validity of the measurement of essential domains were sufficient. |
|  | Bias in selection of participants into the study | Low | - Comment: All participants who would have been eligible for the trial were included in the study, and for each participant start of follow-up and the beginning of intervention coincided. |
|  | Bias in classification of interventions | Low | - Comment: Intervention status is well defined, and intervention definition is based solely on information collected during the intervention. |
|  | Bias due to deviations from intended interventions | Low | - Comment: The critical co-interventions (e.g., routine care) were balanced across experimental and comparator groups. Also, there was no evidence of deviations from the intended interventions likely to impact the outcome. |
|  | Bias due to missing data | Low | - Comment: Data were complete. |
|  | Bias in measurement of outcomes | Low | - Comment: The methods of outcome assessment were comparable across intervention groups. Additionally, the outcome was unlikely to be influenced by knowledge of the intervention received by study participants because it was measured objectively. Moreover, any error in estimating the result seems unrelated to intervention status. |
|  | Bias in selection of the reported result | Moderate | - Comment: A registered protocol is not declared. However, the outcome measurements and analyses are clearly defined and internally and externally consistent. Additionally, there is no indication of the selection of the reported results from multiple analyses. There is also no indication of the cohort selection or subgroups for analysis and reporting based on the results. |
| **Hipni et al., 2022; Megawati et al., 2022** | Bias due to confounding | Moderate | - Comment: Confounding expected, but all known important confounding domains (i.e., parity, gestational and maternal ages at recruitment) were appropriately measured and controlled. Additionally, the reliability and validity of the measurement of essential domains were sufficient. |
|  | Bias in selection of participants into the study | Low | - Comment: All participants who would have been eligible for the trial were included in the study, and for each participant start of follow-up and the beginning of intervention coincided. |
|  | Bias in classification of interventions | Serious | - Comment: Intervention status needs to be better defined. |
|  | Bias due to deviations from intended interventions | Low | - Comment: The critical co-interventions (e.g., routine care) were balanced across experimental and comparator groups. Also, there was no evidence of deviations from the intended interventions likely to impact the outcome. |
|  | Bias due to missing data | Low | - Comment: Data were complete. |
|  | Bias in measurement of outcomes | Low | - Comment: The methods of outcome assessment were comparable across intervention groups. Additionally, the outcome was unlikely to be influenced by knowledge of the intervention received by study participants because it was measured objectively. Moreover, any error in estimating the result seems unrelated to intervention status. |
|  | Bias in selection of the reported result | Serious | - Comment: There is a high risk of selective reporting from multiple analyses. |
| **Sandhi & Dewi, 2022** | Bias due to confounding | Serious | - Comment: Some known essential domains (e.g., parity, gravidity, and maternal age) were not recorded or not controlled. Hence, there is potential for confounding the effect of the intervention. |
|  | Bias in selection of participants into the study | Low | - Comment: All participants who would have been eligible for the trial were included in the study, and for each participant start of follow-up and the beginning of intervention coincided. |
|  | Bias in classification of interventions | Low | - Comment: Intervention status is well defined, and intervention definition is based solely on information collected during the intervention. |
|  | Bias due to deviations from intended interventions | Low | - Comment: The critical co-interventions (e.g., routine care) were balanced across experimental and comparator groups. Also, there was no evidence of deviations from the intended interventions likely to impact the outcome. |
|  | Bias due to missing data | Low | - Comment: Data were complete. |
|  | Bias in measurement of outcomes | Low | - Comment: The methods of outcome assessment were comparable across intervention groups. Additionally, the outcome was unlikely to be influenced by knowledge of the intervention received by study participants because it was measured objectively. Moreover, any error in estimating the result seems unrelated to intervention status. |
|  | Bias in selection of the reported result | Moderate | - Comment: A registered protocol is not declared. However, the outcome measurements and analyses are clearly defined and internally and externally consistent. Additionally, there is no indication of the selection of the reported results from multiple analyses. There is also no indication of the cohort selection or subgroups for analysis and reporting based on the results. |
| **Syarif, 2022** | Bias due to confounding | Serious | - Comment: Some known important domains (i.e., delivery type, mother’s and infant’s ages, infant’s sex, and infant’s birth weight) were not recorded or not controlled. Hence, there is potential for confounding the effect of the intervention. |
|  | Bias in selection of participants into the study | Low | - Comment: All participants who would have been eligible for the trial were included in the study, and for each participant start of follow-up and the beginning of intervention coincided. |
|  | Bias in classification of interventions | Serious | - Comment: Intervention status needs to be better defined. |
|  | Bias due to deviations from intended interventions | Low | - Comment: The critical co-interventions (e.g., routine care) were balanced across experimental and comparator groups. Also, there was no evidence of deviations from the intended interventions likely to impact the outcome. |
|  | Bias due to missing data | Low | - Comment: Data were complete. |
|  | Bias in measurement of outcomes | Low | - Comment: The methods of outcome assessment were comparable across intervention groups. Additionally, the outcome was unlikely to be influenced by knowledge of the intervention received by study participants because it was measured objectively. Moreover, any error in estimating the result seems unrelated to intervention status. |
|  | Bias in selection of the reported result | Moderate | - Comment: A registered protocol is not declared. However, the outcome measurements and analyses are clearly defined and internally and externally consistent. Additionally, there is no indication of the selection of the reported results from multiple analyses. There is also no indication of the cohort selection or subgroups for analysis and reporting based on the results. |
| **Agustina et al., 2021** | Bias due to confounding | Serious | - Comment: Some known important domains (i.e., gravidity, delivery type, mother’s and infant’s ages, infant’s sex, and infant’s birth weight) were not recorded or not controlled. Hence, there is potential for confounding the effect of the intervention. |
|  | Bias in selection of participants into the study | Low | - Comment: All participants who would have been eligible for the trial were included in the study, and for each participant start of follow-up and the beginning of intervention coincided. |
|  | Bias in classification of interventions | Serious | - Comment: Intervention status needs to be better defined. |
|  | Bias due to deviations from intended interventions | Low | - Comment: The critical co-interventions (e.g., routine care) were balanced across experimental and comparator groups. Also, there was no evidence of deviations from the intended interventions likely to impact the outcome. |
|  | Bias due to missing data | Low | - Comment: Data were complete. |
|  | Bias in measurement of outcomes | Low | - Comment: The methods of outcome assessment were comparable across intervention groups. Additionally, the outcome was unlikely to be influenced by knowledge of the intervention received by study participants because it was measured objectively. Moreover, any error in estimating the result seems unrelated to intervention status. |
|  | Bias in selection of the reported result | Moderate | - Comment: A registered protocol is not declared. However, the outcome measurements and analyses are clearly defined and internally and externally consistent. Additionally, there is no indication of the selection of the reported results from multiple analyses. There is also no indication of the cohort selection or subgroups for analysis and reporting based on the results. |
| **Andriani, 2021** | Bias due to confounding | Moderate | - Comment: Confounding expected, but all known important confounding domains (i.e., parity, gravidity, gestational and maternal ages at recruitment) were appropriately measured and controlled. Additionally, the reliability and validity of the measurement of essential domains were sufficient. |
|  | Bias in selection of participants into the study | Low | - Comment: All participants who would have been eligible for the trial were included in the study, and for each participant start of follow-up and the beginning of intervention coincided. |
|  | Bias in classification of interventions | Low | - Comment: Intervention status is well defined, and intervention definition is based solely on information collected during the intervention. |
|  | Bias due to deviations from intended interventions | Low | - Comment: The critical co-interventions (e.g., routine care) were balanced across experimental and comparator groups. Also, there was no evidence of deviations from the intended interventions likely to impact the outcome. |
|  | Bias due to missing data | Low | - Comment: Data were complete. |
|  | Bias in measurement of outcomes | Low | - Comment: The methods of outcome assessment were comparable across intervention groups. Additionally, the outcome was unlikely to be influenced by knowledge of the intervention received by study participants because it was measured objectively. Moreover, any error in estimating the result seems unrelated to intervention status. |
|  | Bias in selection of the reported result | Moderate | - Comment: A registered protocol is not declared. However, the outcome measurements and analyses are clearly defined and internally and externally consistent. Additionally, there is no indication of the selection of the reported results from multiple analyses. There is also no indication of the cohort selection or subgroups for analysis and reporting based on the results. |
| **Choirunissa et al., 2021** | Bias due to confounding | Serious | - Comment: Some known essential domains (e.g., parity, gravidity, gestational and maternal ages at recruitment) were not recorded or not controlled. Hence, there is potential for confounding the effect of the intervention. |
|  | Bias in selection of participants into the study | Low | - Comment: All participants who would have been eligible for the trial were included in the study, and for each participant start of follow-up and the beginning of intervention coincided. |
|  | Bias in classification of interventions | Low | - Comment: Intervention status is well defined, and intervention definition is based solely on information collected during the intervention. |
|  | Bias due to deviations from intended interventions | Low | - Comment: The critical co-interventions (e.g., routine care) were balanced across experimental and comparator groups. Also, there was no evidence of deviations from the intended interventions likely to impact the outcome. |
|  | Bias due to missing data | Low | - Comment: Data were complete. |
|  | Bias in measurement of outcomes | Low | - Comment: The methods of outcome assessment were comparable across intervention groups. Additionally, the outcome was unlikely to be influenced by knowledge of the intervention received by study participants because it was measured in the lab (i.e., Hb). Also, any error in estimating the outcome seems unrelated to intervention status. |
|  | Bias in selection of the reported result | Moderate | - Comment: A registered protocol is not declared. However, the outcome measurements and analyses are clearly defined and internally and externally consistent. Additionally, there is no indication of the selection of the reported results from multiple analyses. There is also no indication of the cohort selection or subgroups for analysis and reporting based on the results. |
| **Dahlan & Ardhi, 2021** | Bias due to confounding | Serious | - Comment: Some known essential domains (e.g., parity, gravidity, gestational and maternal ages at recruitment) were not recorded or not controlled. Hence, there is potential for confounding the effect of the intervention. |
|  | Bias in selection of participants into the study | Low | - Comment: All participants who would have been eligible for the trial were included in the study, and for each participant start of follow-up and the beginning of intervention coincided. |
|  | Bias in classification of interventions | Serious | - Comment: Intervention status needs to be better defined. |
|  | Bias due to deviations from intended interventions | Low | - Comment: The critical co-interventions (e.g., routine care) were balanced across experimental and comparator groups. Also, there was no evidence of deviations from the intended interventions likely to impact the outcome. |
|  | Bias due to missing data | Low | - Comment: Data were complete. |
|  | Bias in measurement of outcomes | Low | - Comment: The methods of outcome assessment were comparable across intervention groups. Additionally, the outcome was unlikely to be influenced by knowledge of the intervention received by study participants because it was measured in the lab (i.e., Hb). Also, any error in estimating the outcome seems unrelated to intervention status. |
|  | Bias in selection of the reported result | Moderate | - Comment: A registered protocol is not declared. However, the outcome measurements and analyses are clearly defined and internally and externally consistent. Additionally, there is no indication of the selection of the reported results from multiple analyses. There is also no indication of the cohort selection or subgroups for analysis and reporting based on the results. |
| **Fauziah & Maulany, 2021** | Bias due to confounding | Serious | - Comment: Some known essential domains (e.g., parity, gravidity, gestational and maternal ages at recruitment) were not recorded or not controlled. Hence, there is potential for confounding the effect of the intervention. |
|  | Bias in selection of participants into the study | Low | - Comment: All participants who would have been eligible for the trial were included in the study, and for each participant start of follow-up and the beginning of intervention coincided. |
|  | Bias in classification of interventions | Low | - Comment: Intervention status is well defined, and intervention definition is based solely on information collected during the intervention. |
|  | Bias due to deviations from intended interventions | Low | - Comment: The critical co-interventions (e.g., routine care) were balanced across experimental and comparator groups. Also, there was no evidence of deviations from the intended interventions likely to impact the outcome. |
|  | Bias due to missing data | Low | - Comment: Data were complete. |
|  | Bias in measurement of outcomes | Low | - Comment: The methods of outcome assessment were comparable across intervention groups. Additionally, the outcome was unlikely to be influenced by knowledge of the intervention received by study participants because it was measured in the lab (i.e., Hb). Also, any error in estimating the outcome seems unrelated to intervention status. |
|  | Bias in selection of the reported result | Moderate | - Comment: A registered protocol is not declared. However, the outcome measurements and analyses are clearly defined and internally and externally consistent. Additionally, there is no indication of the selection of the reported results from multiple analyses. There is also no indication of the cohort selection or subgroups for analysis and reporting based on the results. |
| **Firdausi & Mukhlis, 2021** | Bias due to confounding | Serious | - Comment: Some known essential domains (e.g., gravidity, gestational and maternal ages at recruitment) were not recorded or not controlled. Hence, there is potential for confounding the effect of the intervention. |
|  | Bias in selection of participants into the study | Low | - Comment: All participants who would have been eligible for the trial were included in the study, and for each participant start of follow-up and the beginning of intervention coincided. |
|  | Bias in classification of interventions | Serious | - Comment: Intervention status needs to be better defined. |
|  | Bias due to deviations from intended interventions | Low | - Comment: The critical co-interventions (e.g., routine care) were balanced across experimental and comparator groups. Also, there was no evidence of deviations from the intended interventions likely to impact the outcome. |
|  | Bias due to missing data | Low | - Comment: Data were complete. |
|  | Bias in measurement of outcomes | Low | - Comment: The methods of outcome assessment were comparable across intervention groups. Additionally, the outcome was unlikely to be influenced by knowledge of the intervention received by study participants because it was measured in the lab (i.e., Hb). Also, any error in estimating the outcome seems unrelated to intervention status. |
|  | Bias in selection of the reported result | Moderate | - Comment: A registered protocol is not declared. However, the outcome measurements and analyses are clearly defined and internally and externally consistent. Additionally, there is no indication of the selection of the reported results from multiple analyses. There is also no indication of the cohort selection or subgroups for analysis and reporting based on the results. |
| **Manan et al., 2021** | Bias due to confounding | Serious | - Comment: Some known essential domains (e.g., parity, gravidity, gestational and maternal ages at recruitment) were not recorded or not controlled. Hence, there is potential for confounding the effect of the intervention. |
|  | Bias in selection of participants into the study | Low | - Comment: All participants who would have been eligible for the trial were included in the study, and for each participant start of follow-up and the beginning of intervention coincided. |
|  | Bias in classification of interventions | Low | - Comment: Intervention status is well defined, and intervention definition is based solely on information collected during the intervention. |
|  | Bias due to deviations from intended interventions | Low | - Comment: The critical co-interventions (e.g., routine care) were balanced across experimental and comparator groups. Also, there was no evidence of deviations from the intended interventions likely to impact the outcome. |
|  | Bias due to missing data | Low | - Comment: Data were complete. |
|  | Bias in measurement of outcomes | Low | - Comment: The methods of outcome assessment were comparable across intervention groups. Additionally, the outcome was unlikely to be influenced by knowledge of the intervention received by study participants because it was measured in the lab (i.e., Hb). Also, any error in estimating the outcome seems unrelated to intervention status. |
|  | Bias in selection of the reported result | Moderate | - Comment: A registered protocol is not declared. However, the outcome measurements and analyses are clearly defined and internally and externally consistent. Additionally, there is no indication of the selection of the reported results from multiple analyses. There is also no indication of the cohort selection or subgroups for analysis and reporting based on the results. |

| **Murtiyarini et al., 2021** | Bias due to confounding | Moderate | - Comment: Confounding expected, but all known important confounding domains (i.e., parity, gestational and maternal ages at recruitment) were appropriately measured and controlled. Additionally, the reliability and validity of the measurement of essential domains were sufficient. |
| --- | --- | --- | --- |
|  | Bias in selection of participants into the study | Low | - Comment: All participants who would have been eligible for the trial were included in the study, and for each participant start of follow-up and the beginning of intervention coincided. |
|  | Bias in classification of interventions | Low | - Comment: Intervention status is well defined, and intervention definition is based solely on information collected during the intervention. |
|  | Bias due to deviations from intended interventions | Low | - Comment: The critical co-interventions (e.g., routine care) were balanced across experimental and comparator groups. Also, there was no evidence of deviations from the intended interventions likely to impact the outcome. |
|  | Bias due to missing data | Low | - Comment: Data were complete. |
|  | Bias in measurement of outcomes | Low | - Comment: The methods of outcome assessment were comparable across intervention groups. Additionally, the outcome was unlikely to be influenced by knowledge of the intervention received by study participants because it was measured in the lab (i.e., Hb). Also, any error in estimating the outcome seems unrelated to intervention status. |
|  | Bias in selection of the reported result | Moderate | - Comment: A registered protocol is not declared. However, the outcome measurements and analyses are clearly defined and internally and externally consistent. Additionally, there is no indication of the selection of the reported results from multiple analyses. There is also no indication of the cohort selection or subgroups for analysis and reporting based on the results. |
| **Ramadhani & Akbar, 2021** | Bias due to confounding | Moderate | - Comment: Confounding expected but most known important confounding domains (i.e., parity, mother’s and infant’s ages, and infant’s sex) were appropriately measured and controlled. Additionally, the reliability and validity of the measurement of essential domains were sufficient. |
|  | Bias in selection of participants into the study | Low | - Comment: All participants who would have been eligible for the trial were included in the study, and for each participant start of follow-up and the beginning of intervention coincided. |
|  | Bias in classification of interventions | Serious | - Comment: Intervention status needs to be better defined. |
|  | Bias due to deviations from intended interventions | Low | - Comment: The critical co-interventions (e.g., routine care) were balanced across experimental and comparator groups. Also, there was no evidence of deviations from the intended interventions likely to impact the outcome. |
|  | Bias due to missing data | Low | - Comment: Data were complete. |
|  | Bias in measurement of outcomes | Low | - Comment: The methods of outcome assessment were comparable across intervention groups. Additionally, the outcome was unlikely to be influenced by knowledge of the intervention received by study participants because it was measured objectively. Moreover, any error in estimating the result seems unrelated to intervention status. |
|  | Bias in selection of the reported result | Moderate | - Comment: A registered protocol is not declared. However, the outcome measurements and analyses are clearly defined and internally and externally consistent. Additionally, there is no indication of the selection of the reported results from multiple analyses. There is also no indication of the cohort selection or subgroups for analysis and reporting based on the results. |
| **Triananinsi et al., 2021** | Bias due to confounding | Serious | - Comment: Some known essential domains (e.g., parity, gestational and maternal ages at recruitment) were not recorded or not controlled. Hence, there is potential for confounding the effect of the intervention. |
|  | Bias in selection of participants into the study | Low | - Comment: All participants who would have been eligible for the trial were included in the study, and for each participant start of follow-up and the beginning of intervention coincided. |
|  | Bias in classification of interventions | Low | - Comment: Intervention status is well defined, and intervention definition is based solely on information collected during the intervention. |
|  | Bias due to deviations from intended interventions | Low | - Comment: The critical co-interventions (e.g., routine care) were balanced across experimental and comparator groups. Also, there was no evidence of deviations from the intended interventions likely to impact the outcome. |
|  | Bias due to missing data | Low | - Comment: Data were complete. |
|  | Bias in measurement of outcomes | Low | - Comment: The methods of outcome assessment were comparable across intervention groups. Additionally, the outcome was unlikely to be influenced by knowledge of the intervention received by study participants because it was measured objectively. Moreover, any error in estimating the result seems unrelated to intervention status. |
|  | Bias in selection of the reported result | Moderate | - Comment: A registered protocol is not declared. However, the outcome measurements and analyses are clearly defined and internally and externally consistent. Additionally, there is no indication of the selection of the reported results from multiple analyses. There is also no indication of the cohort selection or subgroups for analysis and reporting based on the results. |
| **Zaher et al., 2021** | Bias due to confounding | Moderate | - Comment: Confounding expected, but all known important confounding domains (i.e., parity, gestational and maternal ages at recruitment) were appropriately measured and controlled. Additionally, the reliability and validity of the measurement of essential domains were sufficient. |
|  | Bias in selection of participants into the study | Moderate | - Comment: All participants who would have been eligible for the trial were included in the study, but the start of follow-up and the beginning of intervention do not coincide for all participants (i.e., it started with the control group, and then for the intervention group). |
|  | Bias in classification of interventions | Low | - Comment: Intervention status is well defined, and intervention definition is based solely on information collected during the intervention. |
|  | Bias due to deviations from intended interventions | Moderate | - Comment: The intervention group did not receive routine hospital care. Hence, an imbalance in co-intervention (i.e., standard care) between experimental intervention and comparator groups could represent a bias. |
|  | Bias due to missing data | Low | - Comment: Data were complete. |
|  | Bias in measurement of outcomes | Low | - Comment: The methods of outcome assessment were comparable across intervention groups. Additionally, the outcome was unlikely to be influenced by knowledge of the intervention received by study participants because it was measured objectively. Moreover, any error in estimating the result seems unrelated to intervention status. |
|  | Bias in selection of the reported result | Moderate | - Comment: A registered protocol is not declared. However, the outcome measurements and analyses are clearly defined and internally and externally consistent. Additionally, there is no indication of the selection of the reported results from multiple analyses. There is also no indication of the cohort selection or subgroups for analysis and reporting based on the results. |
| **Addini et al., 2020** | Bias due to confounding | Serious | - Comment: Some known essential domains (e.g., parity, gravidity, and gestational and maternal ages at recruitment) were not recorded or not controlled. Hence, there is potential for confounding the effect of the intervention. |
|  | Bias in selection of participants into the study | Low | - Comment: All participants who would have been eligible for the trial were included in the study, and for each participant start of follow-up and the beginning of intervention coincided. |
|  | Bias in classification of interventions | Serious | - Comment: Intervention status needs to be better defined. |
|  | Bias due to deviations from intended interventions | Low | - Comment: The critical co-interventions (e.g., routine care) were balanced across experimental and comparator groups. Also, there was no evidence of deviations from the intended interventions likely to impact the outcome. |
|  | Bias due to missing data | Low | - Comment: Data were complete. |
|  | Bias in measurement of outcomes | Low | - Comment: The methods of outcome assessment were comparable across intervention groups. Additionally, the outcome was unlikely to be influenced by knowledge of the intervention received by study participants because it was measured objectively. Moreover, any error in estimating the result seems unrelated to intervention status. |
|  | Bias in selection of the reported result | Moderate | - Comment: A registered protocol is not declared. However, the outcome measurements and analyses are clearly defined and internally and externally consistent. Additionally, there is no indication of the selection of the reported results from multiple analyses. There is also no indication of the cohort selection or subgroups for analysis and reporting based on the results. |
| **Ma’mum et al., 2020** | Bias due to confounding | Serious | - Comment: Some known essential domains (e.g., parity and gestational age at recruitment) were not recorded or not controlled. Hence, there is potential for confounding the effect of the intervention. |
|  | Bias in selection of participants into the study | Low | - Comment: All participants who would have been eligible for the trial were included in the study, and for each participant start of follow-up and the beginning of intervention coincided. |
|  | Bias in classification of interventions | Serious | - Comment: Intervention status needs to be better defined. |
|  | Bias due to deviations from intended interventions | Low | - Comment: The critical co-interventions (e.g., routine care) were balanced across experimental and comparator groups. Also, there was no evidence of deviations from the intended interventions likely to impact the outcome. |
|  | Bias due to missing data | Low | - Comment: Data were complete. |
|  | Bias in measurement of outcomes | Low | - Comment: The methods of outcome assessment were comparable across intervention groups. Additionally, the outcome was unlikely to be influenced by knowledge of the intervention received by study participants because it was measured in the lab (i.e., Hb). Also, any error in estimating the outcome seems unrelated to intervention status. |
|  | Bias in selection of the reported result | Moderate | - Comment: A registered protocol is not declared. However, the outcome measurements and analyses are clearly defined and internally and externally consistent. Additionally, there is no indication of the selection of the reported results from multiple analyses. There is also no indication of the cohort selection or subgroups for analysis and reporting based on the results. |

| **Pongoh et al., 2020** | Bias due to confounding | Serious | - Comment: Some known essential domains (e.g., gestational and maternal ages at recruitment) were not recorded or not controlled. Hence, there is potential for confounding the effect of the intervention. |
| --- | --- | --- | --- |
|  | Bias in selection of participants into the study | Low | - Comment: All participants who would have been eligible for the trial were included in the study, and for each participant start of follow-up and the beginning of intervention coincided. |
|  | Bias in classification of interventions | Low | - Comment: Intervention status is well defined, and intervention definition is based solely on information collected during the intervention. |
|  | Bias due to deviations from intended interventions | Low | - Comment: The critical co-interventions (e.g., routine care) were balanced across experimental and comparator groups. Also, there was no evidence of deviations from the intended interventions likely to impact the outcome. |
|  | Bias due to missing data | Low | - Comment: Data were complete. |
|  | Bias in measurement of outcomes | Low | - Comment: The methods of outcome assessment were comparable across intervention groups. Additionally, the outcome was unlikely to be influenced by knowledge of the intervention received by study participants because it was measured objectively. Moreover, any error in estimating the result seems unrelated to intervention status. |
|  | Bias in selection of the reported result | Moderate | - Comment: A registered protocol is not declared. However, the outcome measurements and analyses are clearly defined and internally and externally consistent. Additionally, there is no indication of the selection of the reported results from multiple analyses. There is also no indication of the cohort selection or subgroups for analysis and reporting based on the results. |
| **Prianti & Eryanti, 2020** | Bias due to confounding | Serious | - Comment: Some known important domains (i.e., parity, delivery type, mother’s and infant’s ages, infant’s sex, and infant’s birth weight) were not recorded or not controlled. Hence, there is potential for confounding the effect of the intervention. |
|  | Bias in selection of participants into the study | Low | - Comment: All participants who would have been eligible for the trial were included in the study, and for each participant start of follow-up and the beginning of intervention coincided. |
|  | Bias in classification of interventions | Serious | - Comment: Intervention status needs to be better defined. |
|  | Bias due to deviations from intended interventions | Low | - Comment: The critical co-interventions (e.g., routine care) were balanced across experimental and comparator groups. Also, there was no evidence of deviations from the intended interventions likely to impact the outcome. |
|  | Bias due to missing data | Low | - Comment: Data were complete. |
|  | Bias in measurement of outcomes | Low | - Comment: The methods of outcome assessment were comparable across intervention groups. Additionally, the outcome was unlikely to be influenced by knowledge of the intervention received by study participants because it was measured objectively. Moreover, any error in estimating the result seems unrelated to intervention status. |
|  | Bias in selection of the reported result | Moderate | - Comment: A registered protocol is not declared. However, the outcome measurements and analyses are clearly defined and internally and externally consistent. Additionally, there is no indication of the selection of the reported results from multiple analyses. There is also no indication of the cohort selection or subgroups for analysis and reporting based on the results. |
| **Sugita & Kuswati, 2020** | Bias due to confounding | Serious | - Comment: Some known essential domains (e.g., gestational and maternal ages at recruitment) were not recorded or not controlled. Hence, there is potential for confounding the effect of the intervention. |
|  | Bias in selection of participants into the study | Low | - Comment: All participants who would have been eligible for the trial were included in the study, and for each participant start of follow-up and the beginning of intervention coincided. |
|  | Bias in classification of interventions | Low | - Comment: Intervention status is well defined, and intervention definition is based solely on information collected during the intervention. |
|  | Bias due to deviations from intended interventions | Low | - Comment: The critical co-interventions (e.g., routine care) were balanced across experimental and comparator groups. Also, there was no evidence of deviations from the intended interventions likely to impact the outcome. |
|  | Bias due to missing data | Low | - Comment: Data were complete. |
|  | Bias in measurement of outcomes | Low | - Comment: The methods of outcome assessment were comparable across intervention groups. Additionally, the outcome was unlikely to be influenced by knowledge of the intervention received by study participants because it was measured in the lab (i.e., Hb). Also, any error in estimating the outcome seems unrelated to intervention status. |
|  | Bias in selection of the reported result | Moderate | - Comment: A registered protocol is not declared. However, the outcome measurements and analyses are clearly defined and internally and externally consistent. Additionally, there is no indication of the selection of the reported results from multiple analyses. There is also no indication of the cohort selection or subgroups for analysis and reporting based on the results. |
| **Yuviska & Yuliasari, 2020** | Bias due to confounding | Serious | - Comment: Some known essential domains (e.g., parity, gravidity, gestational and maternal ages at recruitment) were not recorded or not controlled. Hence, there is potential for confounding the effect of the intervention. |
|  | Bias in selection of participants into the study | Low | - Comment: All participants who would have been eligible for the trial were included in the study, and for each participant start of follow-up and the beginning of intervention coincided. |
|  | Bias in classification of interventions | Low | - Comment: Intervention status is well defined, and intervention definition is based solely on information collected during the intervention. |
|  | Bias due to deviations from intended interventions | Low | - Comment: The critical co-interventions (e.g., routine care) were balanced across experimental and comparator groups. Also, there was no evidence of deviations from the intended interventions likely to impact the outcome. |
|  | Bias due to missing data | Low | - Comment: Data were complete. |
|  | Bias in measurement of outcomes | Low | - Comment: The methods of outcome assessment were comparable across intervention groups. Additionally, the outcome was unlikely to be influenced by knowledge of the intervention received by study participants because it was measured in the lab (i.e., Hb). Also, any error in estimating the outcome seems unrelated to intervention status. |
|  | Bias in selection of the reported result | Moderate | - Comment: A registered protocol is not declared. However, the outcome measurements and analyses are clearly defined and internally and externally consistent. Additionally, there is no indication of the selection of the reported results from multiple analyses. There is also no indication of the cohort selection or subgroups for analysis and reporting based on the results. |
| **Aminah & Purwaningsih, 2019** | Bias due to confounding | Serious | - Comment: Some known important domains (i.e., parity, delivery type, mother’s and infant’s ages, infant’s sex, and infant’s birth weight) were not recorded or not controlled. Hence, there is potential for confounding the effect of the intervention. |
|  | Bias in selection of participants into the study | Low | - Comment: All participants who would have been eligible for the trial were included in the study, and for each participant start of follow-up and the beginning of intervention coincided. |
|  | Bias in classification of interventions | Low | - Comment: Intervention status is well defined, and intervention definition is based solely on information collected during the intervention. |
|  | Bias due to deviations from intended interventions | Low | - Comment: The critical co-interventions (e.g., routine care) were balanced across experimental and comparator groups. Also, there was no evidence of deviations from the intended interventions likely to impact the outcome. |
|  | Bias due to missing data | Low | - Comment: Data were complete. |
|  | Bias in measurement of outcomes | Low | - Comment: The methods of outcome assessment were comparable across intervention groups. Additionally, the outcome was unlikely to be influenced by knowledge of the intervention received by study participants because it was measured objectively. Moreover, any error in estimating the result seems unrelated to intervention status. |
|  | Bias in selection of the reported result | Moderate | - Comment: A registered protocol is not declared. However, the outcome measurements and analyses are clearly defined and internally and externally consistent. Additionally, there is no indication of the selection of the reported results from multiple analyses. There is also no indication of the cohort selection or subgroups for analysis and reporting based on the results. |
| **Astari & Dewi, 2019** | Bias due to confounding | Serious | - Comment: Some known essential domains (e.g., gravidity and maternal ages at recruitment) were not recorded or not controlled. Hence, there is potential for confounding the effect of the intervention. |
|  | Bias in selection of participants into the study | Low | - Comment: All participants who would have been eligible for the trial were included in the study, and for each participant start of follow-up and the beginning of intervention coincided. |
|  | Bias in classification of interventions | Low | - Comment: Intervention status is well defined, and intervention definition is based solely on information collected during the intervention. |
|  | Bias due to deviations from intended interventions | Low | - Comment: The critical co-interventions (e.g., routine care) were balanced across experimental and comparator groups. Also, there was no evidence of deviations from the intended interventions likely to impact the outcome. |
|  | Bias due to missing data | Low | - Comment: Data were complete. |
|  | Bias in measurement of outcomes | Low | - Comment: The methods of outcome assessment were comparable across intervention groups. Additionally, the outcome was unlikely to be influenced by knowledge of the intervention received by study participants because it was measured objectively. Moreover, any error in estimating the result seems unrelated to intervention status. |
|  | Bias in selection of the reported result | Moderate | - Comment: A registered protocol is not declared. However, the outcome measurements and analyses are clearly defined and internally and externally consistent. Additionally, there is no indication of the selection of the reported results from multiple analyses. There is also no indication of the cohort selection or subgroups for analysis and reporting based on the results. |
| **Kuswati & Handayani, 2019** | Bias due to confounding | Moderate | - Comment: Confounding expected, but all known important confounding domains (i.e., parity, gravidity, gestational and maternal ages at recruitment) were appropriately measured and controlled. Additionally, the reliability and validity of the measurement of essential domains were sufficient. |
|  | Bias in selection of participants into the study | Low | - Comment: All participants who would have been eligible for the trial were included in the study, and for each participant start of follow-up and the beginning of intervention coincided. |
|  | Bias in classification of interventions | Low | - Comment: Intervention status is well defined, and intervention definition is based solely on information collected during the intervention. |
|  | Bias due to deviations from intended interventions | Low | - Comment: The critical co-interventions (e.g., routine care) were balanced across experimental and comparator groups. Also, there was no evidence of deviations from the intended interventions likely to impact the outcome. |
|  | Bias due to missing data | Low | - Comment: Data were complete. |
|  | Bias in measurement of outcomes | Low | - Comment: The methods of outcome assessment were comparable across intervention groups. Additionally, the outcome was unlikely to be influenced by knowledge of the intervention received by study participants because it was measured objectively. Moreover, any error in estimating the result seems unrelated to intervention status. |
|  | Bias in selection of the reported result | Moderate | - Comment: A registered protocol is not declared. However, the outcome measurements and analyses are clearly defined and internally and externally consistent. Additionally, there is no indication of the selection of the reported results from multiple analyses. There is also no indication of the cohort selection or subgroups for analysis and reporting based on the results. |
| **Mutiah, 2019** | Bias due to confounding | Serious | - Comment: Some known essential domains (e.g., gravidity, gestational and maternal ages at recruitment) were not recorded or not controlled. Hence, there is potential for confounding the effect of the intervention. |
|  | Bias in selection of participants into the study | Low | - Comment: All participants who would have been eligible for the trial were included in the study, and for each participant start of follow-up and the beginning of intervention coincided. |
|  | Bias in classification of interventions | Serious | - Comment: Intervention status needs to be better defined. |
|  | Bias due to deviations from intended interventions | Low | - Comment: The critical co-interventions (e.g., routine care) were balanced across experimental and comparator groups. Also, there was no evidence of deviations from the intended interventions likely to impact the outcome. |
|  | Bias due to missing data | Low | - Comment: Data were complete. |
|  | Bias in measurement of outcomes | Low | - Comment: The methods of outcome assessment were comparable across intervention groups. Additionally, the outcome was unlikely to be influenced by knowledge of the intervention received by study participants because it was measured objectively. Moreover, any error in estimating the result seems unrelated to intervention status. |
|  | Bias in selection of the reported result | Moderate | - Comment: A registered protocol is not declared. However, the outcome measurements and analyses are clearly defined and internally and externally consistent. Additionally, there is no indication of the selection of the reported results from multiple analyses. There is also no indication of the cohort selection or subgroups for analysis and reporting based on the results. |
| **Astutti et al., 2018** | Bias due to confounding | Serious | - Comment: Some known essential domains (e.g., parity and maternal age at recruitment) were not recorded or not controlled. Hence, there is potential for confounding the effect of the intervention. |
|  | Bias in selection of participants into the study | Low | - Comment: All participants who would have been eligible for the trial were included in the study, and for each participant start of follow-up and the beginning of intervention coincided. |
|  | Bias in classification of interventions | Serious | - Comment: Intervention status needs to be better defined. |
|  | Bias due to deviations from intended interventions | Low | - Comment: The critical co-interventions (e.g., routine care) were balanced across experimental and comparator groups. Also, there was no evidence of deviations from the intended interventions likely to impact the outcome. |
|  | Bias due to missing data | Low | - Comment: Data were complete. |
|  | Bias in measurement of outcomes | Low | - Comment: The methods of outcome assessment were comparable across intervention groups. Additionally, the outcome was unlikely to be influenced by knowledge of the intervention received by study participants because it was measured objectively. Moreover, any error in estimating the result seems unrelated to intervention status. |
|  | Bias in selection of the reported result | Moderate | - Comment: A registered protocol is not declared. However, the outcome measurements and analyses are clearly defined and internally and externally consistent. Additionally, there is no indication of the selection of the reported results from multiple analyses. There is also no indication of the cohort selection or subgroups for analysis and reporting based on the results. |
| **Al-Dossari et al., 2017** | Bias due to confounding | Moderate | - Comment: Confounding expected, but all known important confounding domains (i.e., parity, gravidity, gestational and maternal ages at recruitment) were appropriately measured and controlled. Additionally, the reliability and validity of the measurement of essential domains were sufficient. |
|  | Bias in selection of participants into the study | Low | - Comment: All participants who would have been eligible for the trial were included in the study, and for each participant start of follow-up and the beginning of intervention coincided. |
|  | Bias in classification of interventions | Low | - Comment: Intervention status is well defined, and intervention definition is based solely on information collected during the intervention. |
|  | Bias due to deviations from intended interventions | Low | - Comment: The critical co-interventions (e.g., routine care) were balanced across experimental and comparator groups. Also, there was no evidence of deviations from the intended interventions likely to impact the outcome. |
|  | Bias due to missing data | Low | - Comment: Proportions of and reasons for missing participants were similar across intervention groups. |
|  | Bias in measurement of outcomes | Low | - Comment: The methods of outcome assessment were comparable across intervention groups. Additionally, the outcome was unlikely to be influenced by knowledge of the intervention received by study participants because it was measured objectively. Moreover, any error in estimating the result seems unrelated to intervention status. |
|  | Bias in selection of the reported result | Moderate | - Comment: A registered protocol is not declared. However, the outcome measurements and analyses are clearly defined and internally and externally consistent. Additionally, there is no indication of the selection of the reported results from multiple analyses. There is also no indication of the cohort selection or subgroups for analysis and reporting based on the results. |
| **Putriningtyas & Hidana, 2016** | Bias due to confounding | Moderate | - Comment: Confounding expected, but all known important confounding domains (i.e., gestational age at delivery, delivery type, mother’s and infant’s ages, infant’s sex, and infant’s birth weight) were appropriately measured and controlled. Additionally, the reliability and validity of the measurement of essential domains were sufficient. |
|  | Bias in selection of participants into the study | Low | - Comment: All participants who would have been eligible for the trial were included in the study, and for each participant start of follow-up and the beginning of intervention coincided. |
|  | Bias in classification of interventions | Low | - Comment: Intervention status is well defined, and intervention definition is based solely on information collected during the intervention. |
|  | Bias due to deviations from intended interventions | Low | - Comment: The critical co-interventions (e.g., routine care) were balanced across experimental and comparator groups. Also, there was no evidence of deviations from the intended interventions likely to impact the outcome. |
|  | Bias due to missing data | Low | - Comment: Proportions of and reasons for missing participants were similar across intervention groups. |
|  | Bias in measurement of outcomes | Low | - Comment: The methods of outcome assessment were comparable across intervention groups. Additionally, the outcome was unlikely to be influenced by knowledge of the intervention received by study participants because it was measured objectively. Moreover, any error in estimating the result seems unrelated to intervention status. |
|  | Bias in selection of the reported result | Moderate | - Comment: A registered protocol is not declared. However, the outcome measurements and analyses are clearly defined and internally and externally consistent. Additionally, there is no indication of the selection of the reported results from multiple analyses. There is also no indication of the cohort selection or subgroups for analysis and reporting based on the results. |
| **Rahayu et al., 2016** | Bias due to confounding | Serious | - Comment: Some known essential domains (e.g., parity, gravidity, and maternal age at recruitment) were not recorded or not controlled. Hence, there is potential for confounding the effect of the intervention. |
|  | Bias in selection of participants into the study | Low | - Comment: All participants who would have been eligible for the trial were included in the study, and for each participant start of follow-up and the beginning of intervention coincided. |
|  | Bias in classification of interventions | Low | - Comment: Intervention status is well defined, and intervention definition is based solely on information collected during the intervention. |
|  | Bias due to deviations from intended interventions | Low | - Comment: The critical co-interventions (e.g., routine care) were balanced across experimental and comparator groups. Also, there was no evidence of deviations from the intended interventions likely to impact the outcome. |
|  | Bias due to missing data | Low | - Comment: Data were complete. |
|  | Bias in measurement of outcomes | Low | - Comment: The methods of outcome assessment were comparable across intervention groups. Additionally, the outcome was unlikely to be influenced by knowledge of the intervention received by study participants because it was measured objectively. Moreover, any error in estimating the result seems unrelated to intervention status. |
|  | Bias in selection of the reported result | Moderate | - Comment: A registered protocol is not declared. However, the outcome measurements and analyses are clearly defined and internally and externally consistent. Additionally, there is no indication of the selection of the reported results from multiple analyses. There is also no indication of the cohort selection or subgroups for analysis and reporting based on the results. |
| **Suroso & Paryono, 2016** | Bias due to confounding | Serious | - Comment: Some known essential domains (e.g., gravidity, maternal age at recruitment) were not recorded or not controlled. Hence, there is potential for confounding the effect of the intervention. |
|  | Bias in selection of participants into the study | Low | - Comment: All participants who would have been eligible for the trial were included in the study, and for each participant start of follow-up and the beginning of intervention coincided. |
|  | Bias in classification of interventions | Serious | - Comment: Intervention status needs to be better defined. |
|  | Bias due to deviations from intended interventions | Low | - Comment: The critical co-interventions (e.g., routine care) were balanced across experimental and comparator groups. Also, there was no evidence of deviations from the intended interventions likely to impact the outcome. |
|  | Bias due to missing data | Low | - Comment: Data were complete. |
|  | Bias in measurement of outcomes | Low | - Comment: The methods of outcome assessment were comparable across intervention groups. Additionally, the outcome was unlikely to be influenced by knowledge of the intervention received by study participants because it was measured objectively. Moreover, any error in estimating the result seems unrelated to intervention status. |
|  | Bias in selection of the reported result | Moderate | - Comment: A registered protocol is not declared. However, the outcome measurements and analyses are clearly defined and internally and externally consistent. Additionally, there is no indication of the selection of the reported results from multiple analyses. There is also no indication of the cohort selection or subgroups for analysis and reporting based on the results. |
| **Jayanti, 2014** | Bias due to confounding | Serious | - Comment: Some known essential domains (e.g., parity, maternal and gestational ages at recruitment) were not recorded or not controlled. Hence, there is potential for confounding the effect of the intervention. |
|  | Bias in selection of participants into the study | Low | - Comment: All participants who would have been eligible for the trial were included in the study, and for each participant start of follow-up and the beginning of intervention coincided. |
|  | Bias in classification of interventions | Serious | - Comment: Intervention status needs to be better defined. |
|  | Bias due to deviations from intended interventions | Low | - Comment: The critical co-interventions (e.g., routine care) were balanced across experimental and comparator groups. Also, there was no evidence of deviations from the intended interventions likely to impact the outcome. |
|  | Bias due to missing data | Low | - Comment: Data were complete. |
|  | Bias in measurement of outcomes | Low | - Comment: The methods of outcome assessment were comparable across intervention groups. Additionally, the outcome was unlikely to be influenced by knowledge of the intervention received by study participants because it was measured objectively. Moreover, any error in estimating the result seems unrelated to intervention status. |
|  | Bias in selection of the reported result | Moderate | - Comment: A registered protocol is not declared. However, the outcome measurements and analyses are clearly defined and internally and externally consistent. Additionally, there is no indication of the selection of the reported results from multiple analyses. There is also no indication of the cohort selection or subgroups for analysis and reporting based on the results. |

| **Al-Kuran et al., 2011** | Bias due to confounding | Moderate | - Comment: Confounding expected, but all known important confounding domains (i.e., parity, gestational and maternal ages at recruitment) were appropriately measured and controlled. Additionally, the reliability and validity of the measurement of essential domains were sufficient. |
| --- | --- | --- | --- |
|  | Bias in selection of participants into the study | Low | - Comment: All participants who would have been eligible for the trial were included in the study, and for each participant start of follow-up and the beginning of intervention coincided. |
|  | Bias in classification of interventions | Low | - Comment: Intervention status is well defined, and intervention definition is based solely on information collected during the intervention. |
|  | Bias due to deviations from intended interventions | Low | - Comment: The critical co-interventions (e.g., routine care) were balanced across experimental and comparator groups. Also, there was no evidence of deviations from the intended interventions likely to impact the outcome. |
|  | Bias due to missing data | Low | - Comment: Data were complete. |
|  | Bias in measurement of outcomes | Low | - Comment: The methods of outcome assessment were comparable across intervention groups. Additionally, the outcome was unlikely to be influenced by knowledge of the intervention received by study participants because it was measured objectively. Moreover, any error in estimating the result seems unrelated to intervention status. |
|  | Bias in selection of the reported result | Moderate | - Comment: A registered protocol is not declared. However, the outcome measurements and analyses are clearly defined and internally and externally consistent. Additionally, there is no indication of the selection of the reported results from multiple analyses. There is also no indication of the cohort selection or subgroups for analysis and reporting based on the results. |

**Supplementary Table 9.** GRADE evidence proﬁle: the effects of oral consumption of dates in the peripartum period on childbirth and perinatal outcomes

| **Outcomes: intervention time**  **(number of studies)** | **Quality assessment** | | | | | **Summary of findings** | | | |
| --- | --- | --- | --- | --- | --- | --- | --- | --- | --- |
|  | **Risk of bias** | **Inconsistency** | **Indirectness** | **Imprecision** | **Publication bias** | **Number of participants** | | **Effect (95% CI)** | **Quality of evidence** |
|  |  |  |  |  |  | **Experimental group** | **Control group** |  |  |
| **Gestation length**: late pregnancy (3 RCTs, 1 non-RCT) | Serious^1^ | No serious^2^ | No serious^3^ | No serious^4^ | Undetected^5^ | 303 | 281 | WMD: 1.97 lower (3.24 lower to 0.69 lower)^6^ | ⊕⊕⊕⊖  Moderate^7^ |
| **Duration of labor’s latent phase**: late pregnancy (2 RCTs, 1 non-RCT) | No serious^8^ | No serious^2^ | No serious^3^ | No serious^4^ | Undetected^5^ | 216 | 192 | WMD: 213.66 lower (296.01 lower to 131.31 lower)^6^ | ⊕⊕⊕⊕  High^9^ |
| **Duration of labor’s active phase**: late pregnancy (3 RCTs, 1 non-RCT) | Serious^1^ | Serious^10^ | No serious^3^ | No serious^4^ | Undetected^5^ | 289 | 267 | WMD: 67.93 lower (121.61 lower to 14.24 lower)^6^ | ⊕⊕⊖⊖  Low^11^ |
| **Duration of the first labor stage**: late pregnancy (1 RCT, 5 non-RCTs) | Serious^1^ | Serious^10^ | No serious^3^ | No serious^4^ | Undetected^5^ | 161 | 161 | WMD: 54.96 lower (79.07 lower to 30.85 lower)^6^ | ⊕⊕⊖⊖  Low^11^ |
| **Duration of the second labor stage**: late pregnancy (3 RCTs, 2 non-RCTs) | Serious^1^ | Serious^10^ | No serious^3^ | No serious^4^ | Undetected^5^ | 323 | 299 | WMD: 15.05 lower (31.23 lower to 1.13 higher)^6^ | ⊕⊕⊖⊖  Low^11^ |
| **Duration of the third labor stage**: late pregnancy (3 RCTs, 2 non-RCTs) | Serious^1^ | No serious^2^ | No serious^3^ | No serious^4^ | Undetected^5^ | 323 | 299 | WMD: 1.21 lower (2.12 lower to 0.31 lower)^12^ | ⊕⊕⊕⊖  Moderate^7^ |
| **Duration of total labor**: labor (3 non-RCTs) | Serious^1^ | No serious^2^ | No serious^3^ | Serious^13^ | Undetected^5^ | 54 | 54 | WMD: 1.27 lower (1.91 lower to 0.62 lower)^12^ | ⊕⊕⊖⊖  Low^11^ |
| **Duration of labor’s active phase**: labor (3 RCTs) | Serious^1^ | Serious^10^ | No serious^3^ | No serious^4^ | Undetected^5^ | 130 | 130 | WMD: 88.38 lower (145.25 lower to 31.50 lower)^6^ | ⊕⊕⊖⊖  Low^11^ |
| **Duration of the first labor stage**: labor (1 RCT, 3 non-RCTs) | Serious^1^ | No serious^2^ | No serious^3^ | No serious^4^ | Undetected^5^ | 115 | 114 | WMD: 66.10 lower (96.58 lower to 35.61 lower)^6^ | ⊕⊕⊕⊖  Moderate^7^ |
| **Duration of the second labor stage**: labor (3 RCTs, 3 non-RCTs) | Serious^1^ | Serious^10^ | No serious^3^ | No serious^4^ | Undetected^5^ | 205 | 204 | WMD: 19.46 lower (31.14 lower to 7.79 lower)^6^ | ⊕⊕⊖⊖  Low^11^ |
| **Duration of the third labor stage**: labor (2 RCTs, 3 non-RCTs) | Serious^1^ | Serious^10^ | No serious^3^ | No serious^4^ | Undetected^5^ | 175 | 174 | WMD: 2.61 lower (6.10 lower to 0.88 higher)^6^ | ⊕⊕⊖⊖  Low^11^ |
| **Cervical dilatation two hours post-intervention**: labor (3 RCTs) | No serious^8^ | Serious^10^ | No serious^3^ | Serious^13^ | Undetected^5^ | 94 | 93 | WMD: 0.54 higher (0.11 higher to 0.97 higher)^14^ | ⊕⊕⊖⊖  Low^11^ |
| **Cervical dilatation upon admission**: late pregnancy (4 RCTs, 1 non-RCT) | Serious^1^ | Serious^10^ | No serious^3^ | No serious^4^ | Undetected^5^ | 373 | 351 | WMD: 1.15 higher (0.25 higher to 2.05 higher)^12^ | ⊕⊕⊖⊖  Low^11^ |
| **Bishop score**: late pregnancy (2 RCTs, 1 non-RCT) | Serious^1^ | No serious^2^ | No serious^3^ | No serious^4^ | Undetected^5^ | 187 | 189 | WMD: 2.47 higher (2.00 higher to 2.94 higher)^6^ | ⊕⊕⊕⊖  Moderate^7^ |
| **Frequency of spontaneous onset of labor**: late pregnancy (5 RCTs, 1 non-RCT) | Serious^1^ | Serious^10^ | No serious^3^ | No serious^4^ | Detected^15^ | 428 | 406 | RR: 1.32 higher (1.11 higher to 1.56 higher)^6^ | ⊕⊖⊖⊖  Very low^16^ |
| **Frequency of need for labor induction**: late pregnancy (5 RCTs, 2 non-RCTs) | Serious^1^ | No serious^2^ | No serious^3^ | No serious^4^ | Undetected^5^ | 406 | 407 | RR: 0.48 lower (0.39 lower to 0.60 lower)^17^ | ⊕⊕⊕⊖  Moderate^7^ |
| **Frequency of spontaneous vaginal delivery**: late pregnancy (4 RCTs, 1 non-RCT) | Serious^1^ | No serious^2^ | No serious^3^ | No serious^4^ | Undetected^5^ | 334 | 336 | RR: 1.09 higher (1.01 higher to 1.18 higher)^6^ | ⊕⊕⊕⊖  Moderate^7^ |
| **Frequency of need for instrumental vaginal delivery**: late pregnancy (4 RCTs) | Serious^1^ | Serious^10^ | No serious^3^ | No serious^4^ | Undetected^5^ | 304 | 306 | RR: 0.37 lower (0.09 lower to 1.46 higher)^17^ | ⊕⊕⊖⊖  Low^11^ |
| **Frequency of need for cesarean section delivery**: late pregnancy (4RCTs, 2 non-RCTs) | No serious^8^ | No serious^2^ | No serious^3^ | No serious^4^ | Undetected^5^ | 403 | 381 | RR: 0.75 lower (0.55 lower to 1.01 higher)^14^ | ⊕⊕⊕⊕  High^9^ |
| **Breast milk quantity**: postpartum (1 RCT, 2 non-RCTs) | Serious^1^ | Serious^10^ | No serious^3^ | Serious^13^ | Undetected^5^ | 55 | 53 | WMD: 29.81 higher (7.69 higher to 51.94 higher)^6^ | ⊕⊖⊖⊖  Very low^16^ |
| **Frequency of smoothness of breast milk production**: postpartum (3 non-RCTs) | Serious^1^ | Serious^10^ | No serious^3^ | Serious^13^ | Detected^8^ | 46 | 46 | RR: 2.04 higher (0.41 higher to 10.13 higher)^6^ | ⊕⊖⊖⊖  Very low^16^ |
| **First-day postpartum bleeding rate**: postpartum (4 RCTs) | Serious^1^ | Serious^10^ | No serious^3^ | No serious^4^ | Undetected^5^ | 168 | 172 | WMD: 30.91 lower (53.98 lower to 7.83 lower)^6^ | ⊕⊕⊖⊖  Low^11^ |
| **Maternal hemoglobin levels**: the third trimester of pregnancy (8 non-RCTs) | Serious^1^ | Serious^10^ | No serious^3^ | No serious^4^ | Undetected^5^ | 128 | 128 | WMD: 0.93 higher (0.55 higher to 1.32 higher)^12^ | ⊕⊕⊖⊖  Low^11^ |
| **Abbreviations:** CI, confidence interval; RCTs, randomized controlled trials; RR, risk ratio; WMD, weighted mean difference.  ^1^ Higher percentage of risk of bias domains across the studies was unclear or high.  ^2^ I-squared statistic (*I^2^*) < 50.0%.  ^3^ Studies were sufficiently directed regarding population, intervention protocol, comparator, and intended outcomes.  ^4^ The boundaries of the conﬁdence interval included the overall treatment effect, but the optimal information size (OIS) was met.  ^5^ Begg’s and Egger’s tests (*P* > 0.05).  ^6^ Very large magnitude of effect (1.30 ≤ effect size).  ^7^ We are moderately confident in the effect estimate: The true effect is likely to be close to the estimate of the effect, but there is a possibility that it is substantially different.  ^8^ Higher percentage of risk of bias domains across the studies was low.  ^9^ We are very confident that the true effect lies close to that of the effect estimate.  ^10^ I-squared statistic (*I^2^*) > 70.0%.  ^11^ Our confidence in the effect estimate is limited: The true effect may be substantially different from the estimate of the effect.  ^12^ Large magnitude of effect (0.80 ≤ effect size < 1.30).  ^13^ The boundaries of the conﬁdence interval included the overall treatment effect, and the optimal information size (OIS) was not met.  ^14^ Moderate magnitude of effect (0.50 ≤ effect size < 0.80).  ^15^ Egger’s test (*P* < 0.05).  ^16^ We have very little confidence in the effect estimate: The true effect is likely to be substantially different from the estimate of effect.  ^17^ Low magnitude of effect (effect size < 0.50). | | | | | | | | | |

1. <https://sci.isc.ac/advancedSearch.aspx> [↑](#footnote-ref-1)
2. <https://www.irct.ir/search/advanced> [↑](#footnote-ref-2)
